# First Synthesis of 3-Glycopyranosyl-1,2,4-Triazines and Some Cycloadditions Thereof

**DOI:** 10.3390/molecules27227801

**Published:** 2022-11-12

**Authors:** Éva Bokor, Attila Ferenczi, Mahir Hashimov, Éva Juhász-Tóth, Zsófia Götz, Alshimaa Ibrahim Zaki, László Somsák

**Affiliations:** Department of Organic Chemistry, University of Debrecen, P.O. Box 400, H-4002 Debrecen, Hungary

**Keywords:** *C*-glycosyl compound, 1,2,4-triazine, amidrazone, IEDDA, pyridine

## Abstract

*C*-glycopyranosyl derivatives of six-membered heterocycles are scarcely represented in the chemical literature and the title 3-glycopyranosyl-1,2,4-triazines are completely unknown. In this paper, the first synthesis of this compound class is accomplished by the cyclocondensation of *C*-glycosyl formamidrazones and 1,2-dicarbonyl derivatives. In addition, the synthesis of *C*-glycopyranosyl 1,2,4-triazin-5(4*H*)-ones was also carried out by the transformation of the above formamidrazones with α-keto-carboxylic esters. Inverse electron demand Diels–Alder reactions of 3-glycopyranosyl-1,2,4-triazines with a bicyclononyne derivative yielded the corresponding annulated 2-glycopyranosyl pyridines.

## 1. Introduction

Triazines in general and 1,2,4-triazines in particular are a significant class of six-membered heterocyclic compounds that are constituents of many bioactive molecules, among them marketed drugs [1,2,3].

*C*-glycosyl compounds are one of the most intensively explored types of glycomimetics, compounds that resemble natural glycans in their chemical structure or/and biological activity [4]. While *C*-glycosyl derivatives of five-membered heterocycles are widely known and also studied for their biological effects, those of six-membered heterocycles are barely represented in the literature [5]. Recognising this deficiency, we have started a program to synthesise mostly unknown *C*-glycosylated six-membered heterocycles. Thus far, we have published the syntheses of 2-glycopyranosyl pyrimidines [6,7,8] and 3-glycopyranosyl 1,2,4,5-tetrazines [9].

*C*-glycosyl 1,2,4-triazines are represented in the literature, to the best of our knowledge, by five compounds altogether: an *O*-perbenzoylated 6-(β-d-arabinofuranosyl)-3-amino-1,2,4-triazine, obtained from the corresponding *C*-glycosyl formaldehyde in a multistep one-pot transformation [10]; *O*-perbenzylated 5-(α- and β-d-ribofuranosyl)-3,6-bis(trifluoromethyl)-1,2,4-triazines [11] and their 2-deoxy-ribofuranosyl counterparts [12], prepared by cycloadditions of the corresponding *C*-glycosyl formimidates and 3,6-bis(trifluoromethyl)-1,2,4,5-tetrazine.

In this work, the synthesis of 3-glycopyranosyl-1,2,4-triazines is reported, which compound class is completely unknown in the chemical literature. In addition, some transformations of these triazines in inverse electron demand Diels–Alder (IEDDA) reactions are also described.

## 2. Results and Discussion

For the construction of 3-substituted 1,2,4-triazines, the cyclocondensation of carboxamidrazones with 1,2-dicarbonyl derivatives is one of the most common methods [1,13,14]. Thus, for the synthesis of 3-(β-d-glucopyranosyl)-1,2,4-triazines, such transformations were envisaged starting from *O*-perbenzoylated *C*-β-d-glucopyranosyl formamidrazone **1** [15], prepared earlier in our laboratory to get *C*-glucosyl 1,2,4-triazoles [15] and -triazolones [16]. The ring-closures of **1** with α-keto aldehydes in dry EtOH under heating (Table 1, *i*) provided a set of 5-alkyl- and -aryl-substituted 1,2,4-triazines **2b-f** (Entries 2–4,7,8) in high yields. The cyclisations of **1** with glyoxal and benzil were also carried out, resulting in unsubstituted and 5,6-diphenyl-1,2,4-triazines **2a** and **2g**, respectively, in good yields (Entries 1 and 9).

To test the potential applicability of other starting reagents, the use of methyl ketones or alkynes for the in situ generation of 1,2-dioxo compounds under oxidative conditions [17] was also tried. Thus, one-pot reactions involving the oxidation of acetophenone or phenylacetylene to phenylglyoxal by SeO_2_ or NIS, followed by ring-closure with amidrazone **1**, were carried out (Table 1, *ii* and *iii*) to result in the expected 5-phenyl-1,2,4-triazine **2d** in good yields (Table 1, Entries 5 and 6). A comparison of the yields obtained in the direct (*i*) and one-pot reactions (*ii* and *iii*) showed, however, the superior effectiveness of the former procedure (Entry 4 vs. Entries 5 and 6).

In addition, *O*-debenzoylation of the newly synthesised 3-glycosyl 1,2,4-triazines was also performed under Zemplén conditions to give the unprotected derivatives **3a-g** in good yields (Table 1).

In order to extend the scope of the 3-glycopyranosyl-1,2,4-triazines, the synthesis of peracylated glucosamine derivatives was also investigated. *C*-(2-deoxy-2-phthalimido-3,4,6-tri-*O*-acetyl-β-d-glucopyranosyl)formamidrazone (**5**) was prepared first as a precursor by the reaction of the corresponding iminoester **4** [18] with hydrazine hydrate (Table 2). Heating of **5** with 1,2-dicarbonyl derivatives in EtOH furnished the expected heterocycles **6a–e** in moderate to good yields (Table 2).

The regioselectivity in the formation of **2b–f** and **6b–d** is based on the reactivity pattern of the functional groups involved in the two-step cyclocondensation process. Thus, the condensation between the aldehyde group of higher electrophilicity in the corresponding 1,2-dioxo compound and the hydrazine part of higher nucleophilicity in the amidrazone, as the first step, can be followed by an intramolecular cyclisation of the resulting hydrazone, involving the remaining keto group and the amide-type NH_2_, which leads to 3,5-disubstituted 1,2,4-triazines [13,14].

**Figure 1:**
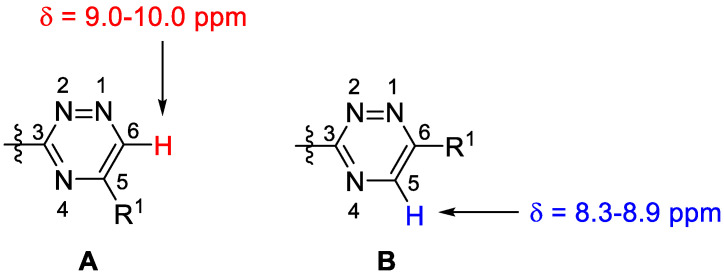
Differentiation of 3,5- and 3,6-disubstituted 1,2,4-triazine isomers by the chemical shift ranges of the H-6/H-5 signals.

The position of the R^1^ substituent in **2b–f** and **6b–d** was also corroborated by ^1^H NMR. According to the literature data, the H-6 resonance of 5-substituted 1,2,4-triazines appears in the range of 9.0–10.0 ppm (Figure 1, **A**), while the corresponding H-5 signal for the isomeric 6-substituted derivatives is found below 9.0 ppm (**B**) [14]. This characteristic singlet for **2b–f** and **6b–d** appeared above 9.0 ppm in each case, providing evidence for the formation of the 5-substituted regioisomers.

*C*-glycopyranosyl formamidrazones **1** and **5** were also used for the preparation of *C*-glycosyl 1,2,4-triazin-5(4*H*)-ones (Table 3). In the reaction of **1** with ethyl glyoxalate or methyl pyruvate in boiling EtOH, a simple condensation took place, providing the corresponding *N*^1^-alkoxycarbonylalkylidene amidrazones **7a,b** in moderate yields. Carrying out the reaction in boiling toluene triggered the desired intramolecular cyclisation of intermediates **7a,b**, accompanied, however, by a 1,2-elimination of benzoic acid from the sugar moiety, yielding glycal derivatives **8a,b**. Similar concomitant elimination was described earlier in the synthesis of a 3-glycosyl-1*H-*1,2,4-triazol-5(4*H*)-one constructed by the thermal ring-closure of *N*^1^-ethoxycarbonyl-*C*-(2,3,4,6-tetra-*O*-benzoyl-β-d-glucopyranosyl)formamidrazone [16]. Other analogous eliminations were reported during the syntheses of *C*-glycopyranosyl heterocycles (e.g., 1,2,4-oxadiazoles and -thiadiazoles, benzimidazoles, perimidines) from the corresponding *O*-peracylated precursors [5].

The cyclisations of amidrazone **5** with the same α-keto esters in boiling toluene were also carried out, producing the expected *C*-glucosaminyl heterocycles **9a,b** in acceptable yields (Table 3).

To demonstrate the synthetic utility of the prepared *C*-glycosyl 1,2,4-triazines, some IEDDA reactions with ((1*R*,8*S*,9*r*)-bicyclo[6.1.0]non-4-yn-9-yl)methanol (**10**, BCN) [19] were performed (Table 4). The [4+2] cycloadditions carried out with triazines **2a,b,d** and **3d** were accomplished in CH_2_Cl_2_ or MeOH at room temperature, producing diastereomeric mixtures of the expected annulated pyridine derivatives **11a,b,d** and **12d** in high yields, respectively.

To get further 2-glucopyranosyl pyridines, the transformations of 1,2,4-triazine **2a** with norbornadiene and 1-pyrrolidino-1-cyclopentene were also attempted. The desired heterocycles could not be obtained even at elevated temperatures (e.g., in boiling *m*-xylene) as no significant conversion of the starting material could be observed, while a slow decomposition of the starting material began after a prolonged reaction time (1 day). This may be due to the lack of an electron-withdrawing substituent on the triazine ring, which could activate this heterocycle towards IEDDA reactions.

## 3. Experimental

### 3.1. General Methods

Melting points were measured on a Kofler hot stage, and the values are uncorrected. Optical rotations were obtained at ambient temperature using a P-2000 polarimeter (Jasco, Easton, MD, USA). The ^1^H and ^13^C NMR spectra of the prepared compounds were recorded with DRX360 (360/90 MHz for ^1^H/^13^C) or DRX400 (400/100 MHz for ^1^H/^13^C) spectrometers (Bruker, Karlsruhe, Germany). Chemical shifts are referenced to Me_4_Si (^1^H-NMR) or to the residual solvent signals (^13^C-NMR). For HRMS measurements, a Bruker maXis II (ESI-HRMS) spectrometer was used, and the data were determined in positive ionisation mode. DC Kieselgel 60 F_254_ plates (Sigma-Aldrich, Saint Louis, MO, USA) were used for TLC analysis, and the spots on the plates were visualised under UV light and developed by gentle heating. Column chromatographic purification was performed by using Kieselgel 60 silica gel (Molar Chemicals, Halásztelek, Hungary, particle size 0.063–0.2 mm). Anhydrous EtOH was purchased from VWR Chemicals. Anhydrous CHCl_3_, toluene and MeOH were obtained by atmospheric distillation from P_4_O_10_ (CHCl_3_ and toluene) or over Mg turnings and iodine (MeOH). *C*-(2,3,4,6-Tetra-*O*-benzoyl-β-d-glucopyranosyl)formamidrazone **1** [15] and ethyl *C*-(2-deoxy-2-phthalimido-3,4,6-tri-*O*-acetyl-β-d-glucopyranosyl)formimidate **4** [18] were synthesised according to our earlier reported methods. ((1*R*,8*S*,9*r*)-Bicyclo [6.1.0]non-4-yn-9-yl)methanol **10** [19] was obtained following a literature procedure.

### 3.2. General Procedure 1 for the Synthesis of O-Peracylated 3-Glycopyranosyl-1,2,4-triazines **2** and **6**

*C*-glycopyranosyl formamidrazone (**1** or **5**) and the appropriate 1,2-dicarbonyl derivative (1.0–1.2 equiv.) were suspended in anhydrous EtOH (3 mL/100 mg substrate), and the mixture was stirred at reflux temperature until the TLC (1:1 EtOAc-hexane) showed completion of the reaction. The solvent was then evaporated under reduced pressure, and the residue was purified by column chromatography.

### 3.3. General Procedure 2 for the O-Debenzoylation of Compounds **2** by the Zemplén Method to obtain 1,2,4-Triazines **3**

To a solution of the corresponding *O*-perbenzoylated 3-(β-d-glucopyranosyl)-1,2,4-triazine (**2**) in dry MeOH (5 mL/100 mg substrate), a catalytic amount of NaOMe in dry MeOH (~1M solution) was added. The reaction mixture was allowed to stand at room temperature, and the transformation was judged by TLC (1:1 EtOAc-hexane and 7:3 CHCl_3_-MeOH). After complete conversion, the reaction mixture was neutralised with a cation exchange resin Amberlyst 15 (H^+^ form). The resin was then filtered off, and MeOH was removed under reduced pressure. The resulting crude product was purified by column chromatography.

### 3.4. General Procedure 3 for the Synthesis of O-Peracylated 3-Glycopyranosyl-1,2,4-triazin-5(4H)-ones **8** and **9**

A solution of the *C*-glycopyranosyl formamidrazone (**1** or **5**) and the corresponding α-ketoester (1 equiv.) in anhydrous toluene (3 mL/100 mg substrate) was refluxed, and the transformation was monitored by TLC (2:1 EtOAc-hexane). After completion of the reaction, the solvent was removed in vacuo, and the residue was purified by column chromatography.

### 3.5. General Procedure 4 for the Preparation of 1-(β-d-Glucopyranosyl)-6,6a,7,7a,8,9-Hexahydro-5H-Cyclopropa[5,6]Cycloocta[1,2-c]Pyridin-7-yl)Methanols **11** and **12**

To a solution of the corresponding 3-glucopyranosyl-1,2,4-triazine (**2** or **3**) in CH_2_Cl_2_ or MeOH (3 mL/100 mg triazine), ((1*R*,8*S*,9*r*)-bicyclo[6.1.0]non-4-yn-9-yl)methanol (**10**, 2 equiv.) was added, and the mixture was stirred at room temperature. When the TLC (1:1 EtOAc-hexane for **11** or 4:1 CHCl_3_-MeOH for **12**) showed complete disappearance of the triazine, the solvent was removed in vacuo. The residue was purified by column chromatography.

### 3.6. Synthesis and Characterisation of the New Compounds

*3-(2,3,4,6-Tetra-O-benzoyl-β-d-glucopyranosyl)-1,2,4-triazine* (**2a**)

Prepared from amidrazone **1** (0.80 g, 1.25 mmol) and an aq. 40 wt.% solution of glyoxal (144 µL, 1.25 mmol) according to general procedure 1. Reaction time: 3 h. Purified by column chromatography (1:2 EtOAc-hexane) to yield 0.75 g (90%) of pale yellow amorphous solids. R_f_ = 0.48 (1:1 EtOAc-hexane); [α]_D_ = −40 (c 0.20, CHCl_3_). ^1^H NMR (360 MHz, CDCl_3_) δ (ppm): 9.17, 8.70 (2 × 1H, 2 d, *J* = 2.1 Hz in each, H-5, H-6), 8.00, 7.95, 7.85, 7.75 (4 × 2H, 4 d, *J* = 7.92 Hz in each, Ar), 7.53-7.26 (12H, m, Ar), 6.18 (1H, pt, *J* = 9.5, 9.5 Hz, H-2′ or H-3′ or H-4′), 6.08 (1H, pt, *J* = 9.7, 9.6 Hz, H-2′ or H-3′ or H-4′), 5.90 (1H, pt, *J* = 9.5, 9.1 Hz, H-2′ or H-3′ or H-4′), 5.39 (1H, d, *J* = 9.7 Hz, H-1′), 4.69 (1H, dd, *J* = 12.1, < 1 Hz, H-6′a), 4.58 (1H, dd, *J* = 12.1, 5.3 Hz, H-6′b), 4.48-4.44 (1H, m, H-5′); ^13^C NMR (90 MHz, CDCl_3_) δ (ppm): 166.1, 165.8, 165.2, 164.8, 164.3 (4 × C=O, C-3), 149.5, 149.4 (C-5, C-6), 133.4, 133.3, 133.2, 133.0, 129.8-129.6, 129.4, 128.7, 128.6, 128.5, 128.4-128.2 (Ar), 80.1, 76.9, 74.1, 71.6, 69.5 (C-1′ − C-5′), 63.4 (C-6′). ESI-HRMS positive mode (m/z): calcd for C_37_H_30_N_3_O_9_^+^ [M+H]^+^ 660.1977; C_37_H_29_N_3_NaO_9_^+^ [M+Na]^+^ 682.1796. Found: [M+H]^+^ 660.1972; [M+Na]^+^ 682.1785.

*3-(2,3,4,6-Tetra-O-benzoyl-β-d-glucopyranosyl)-5-methyl-1,2,4-triazine* (**2b**)

Prepared from amidrazone **1** (0.10 g, 0.16 mmol) and methyl glyoxal (24 µL, 0.16 mmol) according to general procedure 1. Reaction time: 3 h. Purified by column chromatography (2:3 EtOAc-hexane) to yield 89 mg (84%) of pale yellow amorphous solids. R_f_ = 0.38 (1:1 EtOAc-hexane); [α]_D_ = −12 (c 0.20, CHCl_3_). ^1^H NMR (360 MHz, CDCl_3_) δ (ppm): 9.03 (1H, s, H-6), 8.01, 7.94, 7.85, 7.75 (4 × 2H, 4 dd, *J* = 7.2, 1.2 Hz in each, Ar), 7.54-7.28 (12H, m, Ar), 6.14 (1H, pt, *J* = 9.5, 9.1 Hz, H-2′ or H-3′ or H-4′), 6.11 (1H, pt, *J* = 9.7, 9.5 Hz, H-2′ or H-3′ or H-4′), 5.90 (1H, pt, *J* = 9.6, 9.6 Hz, H-2′ or H-3′ or H-4′), 5.31 (1H, d, *J* = 9.3 Hz, H-1′), 4.67 (1H, dd, *J* = 12.3, 3.0 Hz, H-6′a), 4.56 (1H, dd, *J* = 12.3, 5.2 Hz, H-6′b), 4.42 (1H, ddd, *J* = 9.7, 5.2, 3.0 Hz, H-5′), 2.56 (3H, s, CH_3_); ^13^C NMR (90 MHz, CDCl_3_) δ (ppm): 166.1, 165.8, 165.2, 164.7, 163.2 (4 × C=O, C-3), 160.6 (C-5), 149.9 (C-6), 133.4, 133.2, 133.1, 133.0, 129.8-129.7, 129.5, 128.8, 128.7, 128.6, 128.4-128.2 (Ar), 80.1, 76.9, 74.3, 71.4, 69.5 (C-1′ − C-5′), 63.4 (C-6′), 21.8 (CH_3_). ESI-HRMS positive mode (m/z): calcd for C_38_H_32_N_3_O_9_^+^ [M+H]^+^ 674.2133; C_38_H_31_N_3_NaO_9_^+^ [M+Na]^+^ 696.1953. Found: [M+H]^+^ 674.2134; [M+Na]^+^ 696.1950.

*3-(2,3,4,6-Tetra-O-benzoyl-β-d-glucopyranosyl)-5-tert-butyl-1,2,4-triazine* (**2c**)

Prepared from amidrazone **1** (0.30 g, 0.47 mmol) and 3,3-dimethyl-2-oxobutanal (62 mg, 0.47 mmol) according to general procedure 1. Reaction time: 7 h. Purified by column chromatography (3:7 EtOAc-hexane) to yield 0.28 g (83%) of pale yellow amorphous solids. R_f_ = 0.26 (3:7 EtOAc-hexane); [α]_D_ = −11 (c 0.20, CHCl_3_). ^1^H NMR (360 MHz, CDCl_3_) δ (ppm): 9.23 (1H, s, H-6), 8.02, 7.95, 7.86, 7.73 (4 × 2H, 4 dd, *J* = 7.1, 1.2 Hz in each, Ar), 7.54-7.24 (12H, m, Ar), 6.25 (1H, pt, *J* = 9.7, 9.7 Hz, H-2′ or H-3′ or H-4′), 6.13 (1H, pt, *J* = 9.6, 9.5 Hz, H-2′ or H-3′ or H-4′), 5.90 (1H, pt, *J* = 9.7, 9.6 Hz, H-2′ or H-3′ or H-4′), 5.35 (1H, d, *J* = 9.7 Hz, H-1′), 4.69 (1H, dd, *J* = 12.2, 2.9 Hz, H-6′a), 4.53 (1H, dd, *J* = 12.2, 5.0 Hz, H-6′b), 4.44 (1H, ddd, *J* = 9.7, 5.0, 2.9 Hz, H-5′), 1.31 (9H, s, C(CH_3_)_3_); ^13^C NMR (90 MHz, CDCl_3_) δ (ppm): 170.0, 166.1, 165.9, 165.2, 164.5 (4 × C=O, C-3), 162.5 (C-5), 146.8 (C-6), 133.4, 133.1, 133.1, 133.0, 130.0-129.5, 129.5, 128.9, 128.8, 128.4-128.2 (Ar), 80.1, 76.9, 74.4, 71.0, 69.5 (C-1′ − C-5′), 63.2 (C-6′), 36.8 (*C*(CH_3_)_3_), 28.6 (C(*C*H_3_)_3_). ESI-HRMS positive mode (m/z): calcd for C_41_H_38_N_3_O_9_^+^ [M+H]^+^ 716.2603; C_41_H_37_N_3_NaO_9_^+^ [M+Na]^+^ 738.2422. Found: [M+H]^+^ 716.2602; [M+Na]^+^ 738.2419.

*3-(2,3,4,6-Tetra-O-benzoyl-β-d-glucopyranosyl)-5-phenyl-1,2,4-triazine* (**2d**).

**Method A:** Prepared from amidrazone **1** (1.0 g, 1.57 mmol) and phenyl glyoxal monohydrate (0.29 g, 1.88 mmol) according to general procedure 1. Reaction time: 4 h. Purified by column chromatography (1:2 EtOAc-hexane) to yield 0.95 g (83%) of pale yellow amorphous solids. 

**Method B:** To a solution of acetophenone (19 µL, 0.16 mmol, 1 equiv.) in DMSO (2 mL), SeO_2_ (21 mg, 0.19 mmol, 1.2 equiv.) was added. The mixture was heated at 110 °C until the TLC (1:9 EtOAc-hexane) showed the complete transformation of acetophenone (4 h). Formamidrazine **1** (0.10 g, 0.16 mmol) was then added to the mixture, and the heating was continued. When the TLC (1:1 EtOAc-hexane) showed the completion of the reaction (2 h), the mixture was diluted with EtOAc (50 mL) and extracted with water (20 mL). The separated aqueous phase was washed two times with EtOAc (2 × 50 mL). The combined organic phase was dried over MgSO_4_, filtered and the solvent was removed under reduced pressure. The residue was purified by column chromatography (1:2 EtOAc-hexane). Yield: 57 mg (50%).

**Method C:** A solution of phenylacetylene (35 µL, 0.32 mmol, 2 equiv.), NIS (43 mg, 0.19 mmol, 1.2 equiv.) and TsOH (3.4 mg, 0.02 mmol, 0.1 equiv.) in DMSO (2 mL) was heated at 110 °C until the TLC (1:9 EtOAc-hexane) indicated the complete conversion of phenylacetylene (4 h). Formamidrazine **1** (0.10 g, 0.16 mmol) was then added to the mixture, and the heating was continued. After completion of the reaction monitored by TLC (2 h), the same steps as described in method **B** were carried out. Yield: 66 mg (60%). R_f_ = 0.55 (1:1 EtOAc-hexane); [α]_D_ = −68 (c 0.20, CHCl_3_). ^1^H NMR (360 MHz, CDCl_3_) δ (ppm): 9.58 (1H, s, H-6), 8.17, 8.02, 7.96, 7.87, 7.75 (5 × 2H, 5 d, *J* = 7.2 Hz in each, Ar), 7.59-7.21 (15H, m, Ar), 6.33, 6.18, 5.94 (3 × 1H, 3 pt, *J* = 9.7, 9.7 Hz in each, H-2′, H-3′, H-4′), 5.43 (1H, d, *J* = 9.7 Hz, H-1′), 4.72 (1H, dd, *J* = 12.2, 2.8 Hz, H-6′a), 4.55 (1H, dd, *J* = 12.2, 5.1 Hz, H-6′b), 4.49 (1H, ddd, *J* = 9.7, 5.1, 2.8 Hz, H-5′); ^13^C NMR (90 MHz, CDCl_3_) δ (ppm): 166.1, 165.8, 165.2, 164.7, 163.4 (4 × C=O, C-3), 156.0 (C-5), 145.9 (C-6), 133.4, 133.1, 133.0, 132.8, 132.7, 129.8–129.3, 128.8, 128.7, 128.6, 128.5, 128.4–127.9 (Ar), 80.1, 76.9, 74.4, 71.1, 69.5 (C-1′ − C-5′), 63.2 (C-6′). ESI-HRMS positive mode (m/z): calcd for C_43_H_33_N_3_NaO_9_^+^ [M+Na]^+^ 758.2109. Found: 758.2107.

*3-(2,3,4,6-Tetra-O-benzoyl-β-d-glucopyranosyl)-5-(p-methoxyphenyl)-1,2,4-triazine* (**2e**)

Prepared from amidrazone **1** (0.10 g, 0.16 mmol) and *p*-methoxyphenyl glyoxal monohydrate (0.034 g, 0.19 mmol) according to general procedure 1. Reaction time: 4 h. Purification by column chromatography (1:2 EtOAc-hexane), followed by the crystallisation of the resulting syrup from a mixture of Et_2_O (3 mL) and hexane (2 mL), which gave 116 mg (97%) pale yellow crystals. R_f_ = 0.45 (1:1 EtOAc-hexane); mp = 150–151 °C; [α]_D_ = −98 (c 0.53, CHCl_3_). ^1^H NMR (400 MHz, CDCl_3_) δ (ppm): 9.50 (1H, s, H-6), 8.17 (2H, d, *J* = 8.9 Hz, Ar), 8.02, 7.96, 7.87, 7.74 (4 × 2H, 4 dd, *J* = 7.3, 1.2 Hz in each, Ar), 7.54–7.23 (12H, m, Ar), 7.01 (2H, d, *J* = 8.9 Hz, Ar), 6.33, 6.15, 5.92 (3 × 1H, 3 pt, *J* = 9.7, 9.7 Hz in each, H-2′, H-3′, H-4′), 5.37 (1H, d, *J* = 9.7 Hz, H-1′), 4.70 (1H, dd, *J* = 12.3, 2.7 Hz, H-6′a), 4.54 (1H, dd, *J* = 12.3, 5.1 Hz, H-6′b), 4.46 (1H, ddd, *J* = 9.7, 5.1, 2.7 Hz, H-5′), 3.89 (3H, s, OCH_3_); ^13^C NMR (90 MHz, CDCl_3_) δ (ppm): 166.1, 165.9, 165.2, 164.8, 163.6, 163.1 (4 × C=O, C-3, Ar-C_q_), 155.5 (C-5), 145.3 (C-6), 133.4, 133.1, 133.0, 129.8–129.6, 128.9, 128.8, 128.8, 128.4–128.2 (Ar), 125.0 (Ar-C_q_), 114.8 (Ar-CH), 80.1, 76.9, 74.5, 70.9, 69.5 (C-1′ − C-5′), 63.3 (C-6′), 55.5 (OCH_3_). ESI-HRMS positive mode (m/z): calcd for C_44_H_36_N_3_O_10_^+^ [M+H]^+^ 766.2395; C_44_H_35_N_3_NaO_10_^+^ [M+Na]^+^ 788.2215. Found: [M+H]^+^ 766.2390; [M+Na]^+^ 788.2208.

*3-(2,3,4,6-Tetra-O-benzoyl-β-d-glucopyranosyl)-5-(p-chlorophenyl)-1,2,4-triazine* (**2f**)

Prepared from amidrazone **1** (0.10 g, 0.16 mmol) and *p*-chlorophenyl glyoxal monohydrate (0.035 g, 0.19 mmol) according to general procedure 1. Reaction time: 4 h. Purification by column chromatography (1:2 EtOAc-hexane) gave 109 mg (90%) of pale yellow amorphous solids. R_f_ = 0.60 (1:1 EtOAc-hexane); [α]_D_ = −82 (c 0.50, CHCl_3_). ^1^H NMR (400 MHz, CDCl_3_) δ (ppm): 9.57 (1H, s, H-6), 8.13 (2H, d, *J* = 8.4 Hz, Ar), 8.02, 7.96, 7.86, 7.74 (4 × 2H, 4 d, *J* = 7.5 Hz in each, Ar), 7.56-7.24 (14H, m, Ar), 6.28, 6.16, 5.92 (3 × 1H, 3 pt, *J* = 9.6, 9.6 Hz in each, H-2′, H-3′, H-4′), 5.39 (1H, d, *J* = 9.6 Hz, H-1′), 4.72 (1H, dd, *J* = 12.2, 2.1 Hz, H-6′a), 4.54 (1H, dd, *J* = 12.2, 4.9 Hz, H-6′b), 4.47 (1H, ddd, *J* = 9.6, 4.9, 2.1 Hz, H-5′); ^13^C NMR (90 MHz, CDCl_3_) δ (ppm): 166.1, 165.9, 165.2, 164.8, 163.5 (4 × C=O, C-3), 154.9 (C-5), 145.6 (C-6), 139.4 (Ar-C_q_), 133.5, 133.3, 133.2, 133.1 (Ar), 131.3 (Ar-C_q_), 129.9-128.3 (Ar), 80.1, 77.0, 74.4, 71.1, 69.5 (C-1′ − C-5′), 63.2 (C-6′). ESI-HRMS positive mode (m/z): calcd for C_43_H_33_ClN_3_O_9_^+^ [M+H]^+^ 770.1900; C_43_H_32_ClN_3_NaO_9_^+^ [M+Na]^+^ 792.1719. Found: [M+H]^+^ 770.1900; [M+Na]^+^ 792.1718.

*3-(2,3,4,6-Tetra-O-benzoyl-β-d-glucopyranosyl)-5,6-diphenyl-1,2,4-triazine* (**2g**)

Prepared from amidrazone **1** (1.0 g, 1.57 mmol) and benzil (0.33 g, 1.57 mmol) according to general procedure 1. Reaction time: 7 h. Purified by column chromatography (1:1 EtOAc-hexane) to yield 0.67 g (53%) of pale yellow solids. R_f_ = 0.71 (3:7 EtOAc-hexane); mp = 175–179 °C; [α]_D_ = −26 (c 0.20, CHCl_3_); ^1^H NMR (360 MHz, CDCl_3_) δ (ppm): 8.02, 7.95, 7.87, 7.76 (4 × 2H, 4 d, *J* = 7.4 Hz in each, Ar), 7.55–7.24 (22H, m, Ar), 6.36 (1H, pt, *J* = 9.7, 9.7 Hz, H-2′ or H-3′ or H-4′), 6.15 (1H, pt, *J* = 9.6, 9.5 Hz, H-2′ or H-3′ or H-4′), 5.91 (1H, pt, *J* = 9.7, 9.7 Hz, H-2′ or H-3′ or H-4′), 5.45 (1H, d, *J* = 9.7 Hz, H-1′), 4.69 (1H, dd, *J* = 12.3, 2.6 Hz, H-6′a), 4.54 (1H, dd, *J* = 12.3, 5.1 Hz, H-6′b), 4.48 (1H, ddd, *J* = 9.5, 5.1, 2.6 Hz, H-5′); ^13^C NMR (90 MHz, CDCl_3_) δ (ppm): 166.1, 165.9, 165.2, 164.7 (4 × C=O), 161.2, 157.3, 156.5 (C-3, C-5, C-6), 135.0–128.2 (Ar), 79.9, 77.0, 74.6, 71.1, 69.5 (C-1′ − C-5′), 63.3 (C-6′). ESI-HRMS positive mode (m/z): calcd for C_49_H_38_N_3_O_9_^+^ [M+H]^+^ 812.2603. Found: 812.2602.

*3-(β-d-Glucopyranosyl)-1,2,4-triazine* (**3a**)

Prepared from triazine **2a** (0.25 g, 0.38 mmol) according to general procedure 2. Reaction time: 3 h. Purified by column chromatography (4:1 CHCl_3_-MeOH) to yield 58 mg (63%) of pale yellow amorphous solids. R_f_ = 0.32 (7:3 CHCl_3_-MeOH); [α]_D_ = −138 (c 0.22, DMSO). ^1^H NMR (360 MHz, CD_3_OD) δ (ppm): 9.30, 8.84 (2 × 1H, 2 d, *J* = 2.5 Hz in each, H-5, H-6), 4.61 (1H, d, *J* = 9.7 Hz, H-1′), 3.84 (1H, pt, *J* = 9.5, 9.1 Hz, H-2′ or H-3′ or H-4′), 3.83 (1H, dd, *J* = 12.1, 2.0 Hz, H-6′a), 3.68 (1H, dd, *J* = 12.1, 5.0 Hz, H-6′b), 3.56 (1H, pt, *J* = 9.1, 8.9 Hz, H-2′ or H-3′ or H-4′), 3.48-3.46 (2H, m, H-2′ or H-3′ or H-4′, H-5′); ^13^C NMR (90 MHz, CD_3_OD) δ (ppm): 167.6 (C-3), 151.7, 150.9 (C-5, C-6), 83.1, 82.8, 79.4, 74.3, 71.4 (C-1′ − C-5′), 62.8 (C-6′). ESI-HRMS positive mode (m/z): calcd for C_9_H_14_N_3_O_5_^+^ [M+H]^+^ 244.0928; C_9_H_13_N_3_NaO_5_^+^ [M+Na]^+^ 266.0747. Found: [M+H]^+^ 244.0930; [M+Na]^+^ 266.0746.

*3-(β-d-Glucopyranosyl)-5-methyl-1,2,4-triazine* (**3b**)

Prepared from triazine **2b** (0.20 g, 0.3 mmol) according to general procedure 2. Reaction time: 5 h. Purified by column chromatography (4:1 CHCl_3_-MeOH) to yield 44 mg (58%) pale yellow syrup. R_f_ = 0.30 (4:1 CHCl_3_-MeOH); [α]_D_ = +57 (c 0.20, MeOH). ^1^H NMR (360 MHz, CD_3_OD) δ (ppm): 9.26 (1H, s, H-6), 4.58 (1H, d, *J* = 9.7 Hz, H-1′), 3.88 (1H, dd, *J* = 11.9, 2.2 Hz, H-6′a), 3.87 (1H, pt, *J* = 9.4, 9.2 Hz, H-2′ or H-3′ or H-4′), 3.72 (1H, dd, *J* = 11.9, 3.3 Hz, H-6′b), 3.61-3.50 (3H, m, H-2′ and/or H-3′ and/or H-4′, H-5′), 2.63 (3H, s, CH_3_); ^13^C NMR (90 MHz, CD_3_OD) δ (ppm): 166.5, 163.0 (C-3, C-5), 151.2 (C-6), 83.0, 82.8, 79.4, 74.3, 71.4 (C-1′ − C-5′), 62.9 (C-6′), 21.9 (CH_3_). ESI-HRMS positive mode (m/z): calcd for C_10_H_15_N_3_NaO_5_^+^ [M+Na]^+^ 280.0904. Found: 280.0903.

*3-(β-d-Glucopyranosyl)-5-tert-butyl-1,2,4-triazine* (**3c**)

Prepared from triazine **2c** (0.20 g, 0.28 mmol) according to general procedure 2. Reaction time: 5 h. Purified by column chromatography (9:1 CHCl_3_-MeOH) to yield 73 mg (88%) of pale yellow solids. R_f_ = 0.46 (4:1 CHCl_3_-MeOH); mp = 196–200 °C; [α]_D_ = +17 (c 0.20, MeOH). ^1^H NMR (360 MHz, CD_3_OD) δ (ppm): 9.46 (1H, s, H-6), 4.59 (1H, d, *J* = 9.7 Hz, H-1′), 3.98 (1H, pt, *J* = 9.4, 9.2 Hz, H-2′ or H-3′ or H-4′), 3.90 (1H, dd, *J* = 12.0, 1.5 Hz, H-6′a), 3.70 (1H, dd, *J* = 12.0, 5.0 Hz, H-6′b), 3.59 (1H, pt, *J* = 9.0, 8.9 Hz, H-2′ or H-3′ or H-4′), 3.54–3.46 (2H, m, H-2′ or H-3′ or H-4′, H-5′), 1.43 (9H, s, C(CH_3_)_3_); ^13^C NMR (90 MHz, CD_3_OD) δ (ppm): 172.0, 166.2 (C-3, C-5), 148.1 (C-6), 83.4, 82.9, 79.5, 74.2, 71.7 (C-1′ − C-5′), 63.0 (C-6′), 38.0 (*C*(CH_3_)_3_), 29.1 (C(*C*H_3_)_3_). ESI-HRMS positive mode (m/z): calcd for C_13_H_22_N_3_O_5_^+^ [M+H]^+^ 300.1554; C_13_H_21_N_3_NaO_5_^+^ [M+Na]^+^ 322.1373. Found: [M+H]^+^ 300.1550; [M+Na]^+^ 322.1369.

*3-(β-d-Glucopyranosyl)-5-phenyl-1,2,4-triazine* (**3d**)

Prepared from triazine **2d** (0.20 g, 0.27 mmol) according to general procedure 2. Reaction time: 4 h. Purified by column chromatography (9:1 CHCl_3_-MeOH) to yield 62 mg (72%) of pale yellow solids. R_f_ = 0.43 (4:1 CHCl_3_-MeOH); mp = 236–240 °C; [α]_D_ = +44 (c 0.20, DMSO). ^1^H NMR (360 MHz, DMSO-d_6_) δ (ppm): 10.06 (1H, s, H-6), 8.36 (2H, d, *J* = 8.3 Hz, Ar), 7.71–7.62 (3H, m, Ar), 5.11–5.02, 5.57–4.55 (4H, OH), 4.52 (1H, d, *J* = 9.9 Hz, H-1′), 3.98–3.92, 3.75–3.71, 3.48–3.37, 3.27–3.20 (6H, m, H-2′, H-3′, H-4′, H-5′, H-6′a,b); ^13^C NMR (90 MHz, DMSO-d_6_) δ (ppm): 165.2 (C-3), 154.8 (C-5), 146.1 (C-6), 133.0, 132.6, 129.3 (2), 127.8 (2) (Ar), 82.1, 82.0, 77.9, 72.0, 70.4 (C-1′ − C-5′), 61.2 (C-6′). ESI-HRMS positive mode (m/z): calcd for C_15_H_18_N_3_O_5_^+^ [M+H]^+^ 320.1241; C_15_H_17_N_3_NaO_5_^+^ [M+Na]^+^ 342.1060. Found: [M+H]^+^ 320.1239; [M+Na]^+^ 342.1054.

*3-(β-d-Glucopyranosyl)-5-(p-methoxyphenyl)-1,2,4-triazine* (**3e**)

Prepared from triazine **2e** (96 mg, 0.13 mmol) according to general procedure 2. Reaction time: 2 h. Purified by column chromatography (8:1 CHCl_3_-MeOH) to yield 32 mg (73%) of pale yellow amorphous solids. R_f_ = 0.50 (7:3 CHCl_3_-MeOH); [α]_D_ = +24 (c 0.49, DMSO). ^1^H NMR (400 MHz, CD_3_OD) δ (ppm): 9.75 (1H, s, H-6), 8.33, 7.12 (2 × 2H, 2 d, *J* = 8.8 Hz in each, Ar), 4.62 (1H, d, *J* = 9.7 Hz, H-1′), 4.02 (1H, pt, *J* = 9.3, 9.2 Hz, H-2′ or H-3′ or H-4′), 3.92-3.90 (4H, m, H-6′a, OCH_3_), 3.74 (1H, dd, *J* = 11.5, 2.9 Hz, H-6′b), 3.62 (1H, pt, *J* = 9.0, 9.0 Hz, H-2′ or H-3′ or H-4′), 3.55-3.53 (2H, m, H-2′ or H-3′ or H-4′, H-5′); ^13^C NMR (90 MHz, CD_3_OD) δ (ppm): 166.8, 165.4 (C-3, Ar-C_q_), 157.4 (C-5), 146.5 (C-6), 131.1 (2), 126.6, 116.0 (2) (Ar), 83.4, 82.9, 79.5, 74.3, 71.6 (C-1′ − C-5′), 62.9 (C-6′), 56.2 (OCH_3_). ESI-HRMS positive mode (m/z): calcd for C_16_H_20_N_3_O_6_^+^ [M+H]^+^ 350.1347; C_16_H_19_N_3_NaO_6_^+^ [M+Na]^+^ 372.1166. Found: [M+H]^+^ 350.1340; [M+Na]^+^ 372.1159.

*3-(β-d-Glucopyranosyl)-5-(p-chlorophenyl)-1,2,4-triazine* (**3f**)

Prepared from triazine **2f** (79 mg, 0.10 mmol) according to general procedure 2. Reaction time: 2 h. Purified by column chromatography (8:1 CHCl_3_-MeOH) to yield 26 mg (72%) of pale yellow amorphous solids. R_f_ = 0.47 (7:3 CHCl_3_-MeOH); [α]_D_ = +30 (c 0.53, DMSO). ^1^H NMR (400 MHz, CD_3_OD) δ (ppm): 9.87 (1H, s, H-6), 8.36, 7.62 (2 × 2H, 2 d, *J* = 8.6 Hz in each, Ar), 4.67 (1H, d, *J* = 9.7 Hz, H-1′), 4.02 (1H, pt, *J* = 9.3, 9.3 Hz, H-2′ or H-3′ or H-4′), 3.90 (1H, dd, *J* = 12.3, < 1 Hz, H-6′a), 3.73 (1H, dd, *J* = 12.3, 4.9 Hz, H-6′b), 3.62 (1H, pt, *J* = 9.0, 9.0 Hz, H-2′ or H-3′ or H-4′), 3.54-3.53 (2H, m, H-2′ or H-3′ or H-4′, H-5′); ^13^C NMR (90 MHz, CD_3_OD) δ (ppm): ^13^C NMR (90 MHz, CD_3_OD) δ (ppm): 167.0 (C-3), 156.8 (C-5), 147.0 (C-6), 140.4, 133.3, 130.8 (4) (Ar), 83.4, 82.9, 79.5, 74.3, 71.6 (C-1′ − C-5′), 62.9 (C-6′). ESI-HRMS positive mode (m/z): calcd for C_15_H_17_ClN_3_O_5_^+^ [M+H]^+^ 354.0851; C_15_H_16_ClN_3_NaO_5_^+^ [M+Na]^+^ 376.0671. Found: [M+H]^+^ 354.0851; [M+Na]^+^ 376.0668.

*3-(β-d-Glucopyranosyl)-5,6-diphenyl-1,2,4-triazine* (**3g**)

Prepared from triazine **2g** (0.15 g, 0.18 mmol) according to general procedure 2. Reaction time: 4 h. Purified by column chromatography (9:1 CHCl_3_-MeOH) to yield 61 mg (84%) pale yellow oil. R_f_ = 0.32 (9:1 CHCl_3_-MeOH); [α]_D_ = +42 (c 0.44, MeOH). ^1^H NMR (360 MHz, CD_3_OD) δ (ppm): 7.60-7.34 (10H, m, Ar), 4.75 (1H, d, *J* = 9.7 Hz, H-1′), 4.05 (1H, pt, *J* = 9.5, 9.1 Hz, H-2′ or H-3′ or H-4′), 3.92 (1H, dd, *J* = 12.3, < 1 Hz, H-6′a), 3.75 (1H, dd, *J* = 12.3, 4.5 Hz, H-6′b), 3.65 (1H, pt, *J* = 9.1, 9.0 Hz, H-2′ or H-3′ or H-4′), 3.57-3.55 (2H, m, H-2′ or H-3′ or H-4′, H-5′); ^13^C NMR (90 MHz, CD_3_OD) δ (ppm): ^13^C NMR (90 MHz, CD_3_OD) δ (ppm): 165.0 (C-3), 158.8, 158.7 (C-5, C-6), 136.8, 136.7, 132.0, 131.2 (2), 131.0, 130.7 (2), 129.8 (2), 129.6 (2) (Ar), 83.0, 82.9, 79.5, 74.3, 71.5 (C-1′ − C-5′), 62.9 (C-6′). ESI-HRMS positive mode (m/z): calcd for C_21_H_22_N_3_O_5_^+^ [M+H]^+^ 396.1554; C_21_H_21_N_3_NaO_5_^+^ [M+Na]^+^ 418.1373. Found: [M+H]^+^ 396.1555; [M+Na]^+^ 418.1371.

*C-(2-Deoxy-2-phthalimido-3,4,6-tri-O-acetyl-β-d-glucopyranosyl)formamidrazone* (**5**)

Ethyl *C*-(2-deoxy-2-phthalimido-3,4,6-tri-*O*-acetyl-β-d-glucopyranosyl)formimidate (**4**, 1.56 g, 3.17 mmol) was dissolved in anhydrous EtOH (30 mL), and hydrazine monohydrate (154 µL, 3.17 mmol) was added. The reaction mixture was stirred at room temperature, and the transformation of **4** was monitored by TLC (3:2 EtOAc-hexane). After the completion of the reaction (4.5 h), the precipitated product was filtered off and washed with EtOH to give 0.77 g of white solids. The mother liquor was evaporated under reduced pressure to give an oil, which was triturated with diethyl ether to give an additional 0.55 g of white solids. The combined yield of the product: 1.31 g (87%). R_f_ = 0.32 (3:2 EtOAc-hexane); [α]_D_ = −13 (c 0.49, MeOH). ^1^H NMR (360 MHz, CDCl_3_) δ (ppm): 7.86-7.74 (4H, m, Ar), 5.98 (1H, pt, *J* = 9.8, 9.7 Hz, H-2 or H-3 or H-4), 5.19 (1H, pt, *J* = 9.7, 9.6 Hz, H-2 or H-3 or H-4), 4.81 (1H, d, *J* = 9.9 Hz, H-1), 4.58 (2H, br s, NH_2_), 4.50 (1H, pt, *J* = 9.9, 9.6 Hz, H-2 or H-3 or H-4), 4.35 (1H, dd, *J* = 12.3, 4.6 Hz, H-6a), 4.17 (1H, dd, *J* = 12.5, 2.4 Hz, H-6b), 3.98 (1H, ddd, *J* = 9.9, 4.6, 2.4 Hz H-5′), 3.61-3.27 (2H, br signal, NH_2_), 2.11, 2.05, 1.88 (3 × 3H, 3 s, CH_3_); ^13^C NMR (90 MHz, CDCl_3_) δ (ppm): 170.6, 169.9, 169.5 (CH_3_-*C*=O), 167.8, 167.4 (Phth-*C*=O), 148.2 (C=N), 134.2, 134.0, 131.6, 131.0, 123.7, 123.3 (Ar), 75.6, 74.2, 70.9, 68.8 (C-1, C-3 − C-5), 62.1 (C-6), 52.7 (C-2), 20.7, 20.5, 20.4 (3 × CH_3_). ESI-HRMS positive mode (m/z): calcd for C_21_H_25_N_4_O_9_^+^ [M+H]^+^ 477.1616. Found: 477.1613.

*3-(2′-Deoxy-2′-phthalimido-3′,4′,6′-tri-O-acetyl-β-d-glucopyranosyl)-1,2,4-triazine* (**6a**)

Prepared from amidrazone **5** (0.10 g, 0.21 mmol) and an aq. 40 wt.% solution of glyoxal (23.8 µL, 0.21 mmol) according to general procedure 1. Purified by column chromatography (1:1 EtOAc-hexane) to yield 58 mg (58%) of pale yellow syrup. R_f_ = 0.36 (3:2 EtOAc-hexane); [α]_D_ = −52 (c 0.23, CHCl_3_). ^1^H NMR (360 MHz, CDCl_3_) δ (ppm): 9.16, 8.67 (2 × 1H, 2 d, *J* = 2.4 Hz in each, H-5, H-6), 7.80-7.69 (4H, m, Ar), 6.09 (1H, dd, *J* = 10.3, 9.2 Hz, H-2′ or H-3′ or H-4′), 5.96 (1H, d, *J* = 10.5 Hz, H-1′), 5.36 (1H, dd, *J* = 9.9, 9.3 Hz, H-2′ or H-3′ or H-4′), 5.00 (1H, pt, *J* = 10.5, 10.5 Hz, H-2′ or H-3′ or H-4′), 4.38 (1H, dd, *J* = 12.4, 5.1 Hz, H-6′a), 4.23 (1H, dd, *J* = 12.5, 2.0 Hz, H-6′b), 4.17 (1H, ddd, *J* = 10.3, 5.1, 2.0 Hz, H-5′), 2.10, 2.08, 1.88 (3 × 3H, 3 s, CH_3_); ^13^C NMR (90 MHz, CDCl_3_) δ (ppm): 170.6, 170.1, 169.4 (CH_3_-*C*=O), 167.6, 166.9 (Phth-*C*=O), 164.4 (C-3), 149.4 (2) (C-5, C-6), 134.3, 131.3, 130.8, 123.6 (Ar), 76.8, 76.5, 71.3, 68.8 (C-1′, C-3′ − C-5′), 62.2 (C-6′), 53.2 (C-2′), 20.7, 20.6, 20.4 (3 × CH_3_).). ESI-HRMS positive mode (m/z): calcd for C_23_H_23_N_4_O_9_^+^ [M+H]^+^ 499.1460; C_23_H_22_N_4_NaO_9_^+^ [M+Na]^+^ 521.1279. Found: [M+H]^+^ 499.1455; [M+Na]^+^ 521.1279.

*3-(2′-Deoxy-2′-phthalimido-3′,4′,6′-tri-O-acetyl-β-d-glucopyranosyl)-5-methyl-1,2,4-triazine* (**6b**)

Prepared from amidrazone **5** (0.10 g, 0.21 mmol) and an aq. 40 wt.% solution of methylglyoxal (32 µL, 0.21 mmol) according to general procedure 1. Purified by column chromatography (2:1 EtOAc-hexane) to yield 67 mg (62%) of pale yellow syrup. R_f_ = 0.41 (2:1 EtOAc-hexane); [α]_D_ = −16 (c 0.25, CHCl_3_). ^1^H NMR (360 MHz, CDCl_3_) δ (ppm): 9.00 (1H, s, H-6), 7.86-7.69 (4H, m, Ar), 6.07 (1H, dd, *J* = 10.3, 9.3 Hz, H-2′ or H-3′ or H-4′), 5.88 (1H, d, *J* = 10.5 Hz, H-1′), 5.37 (1H, pt, *J* = 9.9, 9.4 Hz, H-2′ or H-3′ or H-4′), 5.05 (1H, pt, *J* = 10.5, 10.5 Hz, H-2′ or H-3′ or H-4′), 4.38 (1H, dd, *J* = 12.5, 5.0 Hz, H-6′a), 4.23 (1H, dd, *J* = 12.5, 2.1 Hz, H-6′b), 4.15 (1H, ddd, *J* = 10.2, 5.0, 2.1 Hz, H-5′), 2.52 (3H, s, CH_3_), 2.10, 2.08, 1.88 (3 × 3H, 3 s, CH_3_); ^13^C NMR (90 MHz, CDCl_3_) δ (ppm): 170.7, 170.2, 169.5 (CH_3_-*C*=O), 167.8, 166.4 (Phth-*C*=O), 163.2. 160.5 (C-3, C-5), 149.9 (C-6), 134.2, 131.4, 131.0, 123.6 (Ar), 76.8, 76.5, 71.4, 68.8 (C-1′, C-3′ − C-5′), 62.2 (C-6′), 53.0 (C-2′), 21.7, 20.7, 20.6, 20.4 (4 × CH_3_). ESI-HRMS positive mode (m/z): calcd for C_24_H_25_N_4_O_9_^+^ [M+H]^+^ 513.1616; C_24_H_24_N_4_NaO_9_^+^ [M+Na]^+^ 535.1435. Found: [M+H]^+^ 513.1612; [M+Na]^+^ 535.1429.

*3-(2′-Deoxy-2′-phthalimido-3′,4′,6′-tri-O-acetyl-β-d-glucopyranosyl)-5-tert-butyl-1,2,4-triazine* (**6c**)

Prepared from amidrazone **5** (0.10 g, 0.21 mmol) and 3,3-dimethyl-2-oxobutanal (0.028 g, 0.21 mmol) according to general procedure 1. Purified by column chromatography (4:5 EtOAc-hexane) to yield 78 mg (67%) of white syrup. R_f_ = 0.51 (4:5 EtOAc-hexane); [α]_D_ = +14 (c 0.28, CHCl_3_). ^1^H NMR (360 MHz, CDCl_3_) δ (ppm): 9.21 (1H, s, H-6), 7.81-7.68 (4H, m, Ar), 6.05 (1H, pt, *J* = 10.3, 9.3 Hz, H-2′ or H-3′ or H-4′), 5.93 (1H, d, *J* = 10.5 Hz, H-1′), 5.37 (1H, pt, *J* = 10.0, 9.3 Hz, H-2′ or H-3′ or H-4′), 5.11 (1H, pt, *J* = 10.5, 10.5 Hz, H-2′ or H-3′ or H-4′), 4.35 (1H, dd, *J* = 12.4, 4.9 Hz, H-6′a), 4.25 (1H, dd, *J* = 12.4, 1.8 Hz, H-6′b), 4.15 (1H, ddd, *J* = 10.0, 4.9, 1.8 Hz, H-5′), 2.10, 2.08, 1.88 (3 × 3H, 3 s, CH_3_), 1.28 (9H, s, C(CH_3_)_3_); ^13^C NMR (90 MHz, CDCl_3_) δ (ppm): 170.6, 170.1, 169.7, 169.5 (3 × CH_3_-*C*=O, C-5), 167.5, 166.5 (2 × Phth-*C*=O), 162.6 (C-3), 146.8 (C-6), 134.2, 131.4, 130.9, 123.4 (Ar), 76.9, 76.5, 71.5, 68.9 (C-1′, C-3′ − C-5′), 62.2 (C-6′), 52.9 (C-2′), 36.7 (*C*(CH_3_)_3_), 28.5 (C(*C*H_3_)_3_), 20.7, 20.6, 20.4 (3 × CH_3_). ESI-HRMS positive mode (m/z): calcd for C_27_H_31_N_4_O_9_^+^ [M+H]^+^ 555.2086; C_27_H_30_N_4_NaO_9_^+^ [M+Na]^+^ 577.1905. Found: [M+H]^+^ 555.2088; [M+Na]^+^ 577.1904.

*3-(2′-Deoxy-2′-phthalimido-3′,4′,6′-tri-O-acetyl-β-d-glucopyranosyl)-5-phenyl-1,2,4-triazine* (**6d**)

Prepared from amidrazone **5** (0.10 g, 0.21 mmol) and phenylglyoxal monohydrate (0.032 g, 0.21 mmol) according to general procedure 1. Purified by column chromatography (1:1 EtOAc-hexane) to yield 88 mg (73%) of yellow syrup. R_f_ = 0.41 (3:2 EtOAc-hexane); [α]_D_ = −86 (c 0.22, CHCl_3_). ^1^H NMR (360 MHz, CDCl_3_) δ (ppm): 9.57 (1H, s, H-6), 8.17–7.52 (9H, m, Ar), 6.10 (1H, pt, *J* = 9.8, 9.7 Hz, H-2′ or H-3′ or H-4′), 6.01 (1H, d, *J* = 10.5 Hz, H-1′), 5.40 (1H, pt, *J* = 9.7, 9.5 Hz, H-2′ or H-3′ or H-4′), 5.24 (1H, pt, *J* = 10.5, 10.5 Hz, H-2′ or H-3′ or H-4′), 4.38 (1H, dd, *J* = 12.3, 4.8 Hz, H-6′a), 4.26-4.17 (2H, m, H-5′, H-6′b), 2.09, 1.90 (9H, 2 s, CH_3_). ^13^C NMR (90 MHz, CDCl_3_) δ (ppm): 170.7, 170.2, 169.5 (CH_3_-*C*=O), 167.8, 167.7 (Phth-*C*=O), 163.4, 155.8 (C-3, C-5), 145.9 (C-6), 134.2, 132.8, 132.6, 129.3, 127.8, 123.5 (Ar), 76.9, 76.6, 71.6, 68.8 (C-1′, C-3′ − C-5′), 62.2 (C-6′), 52.8 (C-2′), 20.7, 20.6, 20.4 (3 × CH_3_). ESI-HRMS positive mode (m/z): calcd for C_29_H_27_N_4_O_9_^+^ [M+H]^+^ 575.1773; C_29_H_26_N_4_NaO_9_^+^ [M+Na]^+^ 597.1592. Found: [M+H]^+^ 575.1777; [M+Na]^+^ 597.1593.

*3-(2′-Deoxy-2′-phthalimido-3′,4′,6′-tri-O-acetyl-β-d-glucopyranosyl)-5,6-diphenyl-1,2,4-triazine* (**6e**)

Prepared from amidrazone **5** (0.10 g, 0.21 mmol) and benzil (0.042 g, 0.21 mmol) according to general procedure 1. Purified by column chromatography (4:5 EtOAc-hexane) to yield 84 mg (65%) of pale yellow syrup. R_f_ = 0.51 (4:5 EtOAc-hexane); [α]_D_ = −60 (c 0.20, CHCl_3_). ^1^H NMR (360 MHz, CDCl_3_) δ (ppm): 7.81-7.27 (14H, m, Ar), 6.10 (1H, dd, *J* = 10.3, 9.3 Hz, H-2′ or H-3′ or H-4′), 6.05 (1H, d, *J* = 10.6 Hz, H-1′), 5.40 (1H, pt, *J* = 10.0, 9.3 Hz, H-2′ or H-3′ or H-4′), 5.30 (1H, pt, *J* = 10.5, 10.5 Hz, H-2′ or H-3′ or H-4′), 4.39 (1H, dd, *J* = 12.6, 5.1 Hz, H-6′a), 4.25 (1H, dd, *J* = 12.6, < 1 Hz, H-6′a), 4.21-4.19 (1H, m, H-5′), 2.08, 1.90 (9H, 2 s, CH_3_). ^13^C NMR (90 MHz, CDCl_3_) δ (ppm): 170.6, 170.1, 169.4 (CH_3_-*C*=O), 167.8, 166.8 (Phth-*C*=O), 161.1, 157.1, 156.2 (C-3, C-5, C-6), 134.8-123.4 (Ar), 76.6, 76.5, 71.6, 68.8 (C-1′, C-3′ − C-5′), 62.2 (C-6′), 52.7 (C-2′), 20.7, 20.6, 20.4 (3 × CH_3_). ESI-HRMS positive mode (m/z): calcd for C_35_H_31_N_4_O_9_^+^ [M+H]^+^ 651.2086; C_35_H_30_N_4_NaO_9_^+^ [M+Na]^+^ 673.1905. Found: [M+H]^+^ 651.2085; [M+Na]^+^ 673.1902.

*N^1^-Ethoxycarbonylmethylidene-C-(2′,3′,4′,6′-tetra-O-benzoyl-β-d-glucopyranosyl)formamidrazone* (**7a**)

A solution of amidrazone **1** (0.10 g, 0.16 mmol) and ethyl glyoxalate (31 µL, 0.16 mmol, an 50% solution in toluene) was boiled in dry EtOH (3 mL) until the TLC (1:1 and 7:3 EtOAc-hexane) showed the complete conversion of **1**. After the completion of the reaction (1 h), the solvent was removed under reduced pressure. After column chromatographic purification (2:3 EtOAc-hexane), 44 mg (39%) of the title compound was obtained as a pale yellow syrup. R_f_ = 0.53 (1:1 EtOAc-hexane); [α]_D_ = +23 (c 0.27, CHCl_3_). ^1^H NMR (360 MHz, CDCl_3_) δ (ppm): 8.06-7.83, 7.59-7.26, 7.07 (21H, m, Ar, C*H*=N), 6.02 (1H, pt, *J* = 9.6, 9.6 Hz, H-2′ or H-3′ or H-4′), 5.76 (1H, pt, *J* = 9.8, 9.7 Hz, H-2′ or H-3′ or H-4′), 5.67 (1H, pt, *J* = 9.7, 9.7 Hz, H-2′ or H-3′ or H-4′), 4.69 (1H, dd, *J* = 12.4, 2.7 Hz, H-6′a), 4.54 (1H, dd, *J* = 12.4, 5.2 Hz, H-6′b), 4.51 (1H, d, *J* = 9.7 Hz, H-1′), 4.35-4.25 (1H, m, H-5′), 4.23 (2H, q, *J* = 7.1 Hz, CH_2_), 1.28 (3H, t, *J* = 7.1 Hz, CH_3_); ^13^C NMR (90 MHz, CDCl_3_) δ (ppm): 166.2, 165.6, 165.3, 165.2, 163.7, 160.9 (5 × C=O, C=N), 146.1 (CH=N), 133.6, 133.3, 133.2, 129.9-128.3 (Ar), 76.4, 76.3, 73.4, 70.5, 69.1 (C-1′ − C-5′), 62.8, 61.2 (C-6′, CH_2_), 14.1 (CH_3_).

*N^1^-Methoxycarbonylethylidene-C-(2′,3′,4′,6′-tetra-O-benzoyl-β-d-glucopyranosyl)formamidrazone* (**7b**)

A solution of amidrazone **1** (0.10 g, 0.16 mmol) and methyl pyruvate (14 µL, 0.16 mmol) was boiled in dry EtOH (3 mL) until the TLC (1:1 and 7:3 EtOAc-hexane) showed the complete conversion of **1**. After the completion of the reaction (1.5 h), the solvent was removed under reduced pressure. After column chromatographic purification (2:3 EtOAc-hexane), 67 mg (59%) of the title compound was obtained as a pale yellow syrup. R_f_ = 0.53 (1:1 EtOAc-hexane). ^1^H NMR (360 MHz, CDCl_3_) δ (ppm): 8.05-7.83, 7.58-7.25, 7.07 (20H, m, Ar), 6.03 (1H, pt, *J* = 9.6, 9.6 Hz, H-2′ or H-3′ or H-4′), 5.94-5.83 (2H, broad signal, NH), 5.75 (1H, pt, *J* = 9.8, 9.7 Hz, H-2′ or H-3′ or H-4′), 5.73 (1H, pt, *J* = 9.6, 9.6 Hz, H-2′ or H-3′ or H-4′), 4.68 (1H, dd, *J* = 12.3, < 1 Hz, H-6′a), 4.55 (1H, dd, *J* = 12.4, 5.4 Hz, H-6′b), 4.55 (1H, d, *J* = 9.7 Hz, H-1′), 4.27 (1H, ddd, *J* = 9.7, 5.4, 2.3 Hz, H-5′), 3.75 (3H, s, OCH_3_), 1.60 (3H, s, CH_3_); ^13^C NMR (90 MHz, CDCl_3_) δ (ppm): 166.1, 165.7, 165.6, 165.2, 165.0 (5 × C=O), 158.3, 153.7 (2 × C=N), 133.6, 133.5, 133.4, 133.2, 129.8-128.3 (Ar), 76.8, 76.4, 73.6, 70.5, 69.2 (C-1′ − C-5′), 62.9 (C-6′), 52.3 (OCH_3_), 13.0 (CH_3_).

*3-(3′,4′,6′-Tri-O-benzoyl-2′-deoxy-d-arabino-hex-1′-enopyranosyl)-1,2,4-triazin-5(4H)-one* (**8a**)

Prepared from amidrazone **1** (0.10 g, 0.16 mmol) and ethyl glyoxalate (31 µL, 0.16 mmol, an 50% solution in toluene) according to general procedure 3. Reaction time: 3 d. Purified by column chromatography (7:3 EtOAc-hexane) to yield 50 mg (57%) of pale yellow amorphous solids. R_f_ = 0.24 (7:3 EtOAc-hexane); [α]_D_ = −44 (c 0.26, CHCl_3_). ^1^H NMR (400 MHz, CDCl_3_) δ (ppm): 14.05 (1H, br s, NH), 7.97-7.93, 7.67-7.64, 7.53-7.49 (16H, m, Ar, H-6), 6.60 (1H, d, *J* = 3.5 Hz, H-2′), 6.00 (1H, dd, *J* = 5.6, 3.5 Hz, H-3′), 5.87 (1H, pt, *J* = 7.6, 5.6 Hz, H-4′), 5.15 (1H, ddd, *J* = 7.6, 4.6, 3.3 Hz, H-5′), 4.82 (1H, dd, *J* = 12.4, 4.6 Hz, H-6′a), 4.74 (1H, dd, *J* = 12.4, 3.3 Hz, H-6′b); ^13^C NMR (90 MHz, CDCl_3_) δ (ppm): 165.3, 164.9, 164.2 (3 × C=O), 161.8, 152.6, 144.6 (C-3, C-5, C-6), 145.4 (C-1′), 133.8, 133.6, 133.4, 129.3-128.6 (Ar), 104.0 (C-2′), 74.8, 67.8, 66.8 (C-3′ − C-5′), 61.2 (C-6′). ESI-HRMS positive mode (m/z): calcd for C_30_H_23_N_3_NaO_8_^+^ [M+Na]^+^ 576.1377. Found: 576.1377.

*3-(3′,4′,6′-Tri-O-benzoyl-2′-deoxy-d-arabino-hex-1′-enopyranosyl)-6-methyl-1,2,4-triazin-5(4H)-one* (**8b**)

Prepared from amidrazone **1** (0.10 g, 0.16 mmol) and methyl pyruvate (14 µL, 0.16 mmol) according to general procedure 3. Reaction time: 6 h. Purified by column chromatography (7:3 EtOAc-hexane) to yield 67 mg (75%) of pale yellow amorphous solids. R_f_ = 0.42 (7:3 EtOAc-hexane); [α]_D_ = −29 (c 0.23, CHCl_3_). ^1^H NMR (400 MHz, CDCl_3_) δ (ppm): 13.82 (1H, br s, NH), 7.96-7.95, 7.68-7.64, 7.53-7.49 (15H, m, Ar), 6.37 (1H, broad signal, H-2′), 5.98 (1H, broad signal, H-3′), 5.87 (1H, pt, *J* = 7.0, 6.0 Hz, H-4′), 5.14 (1H, broad signal, H-5′), 4.82 (1H, dd, *J* = 12.3, 4.6 Hz, H-6′a), 4.73 (1H, dd, *J* = 12.3, 2.5 Hz, H-6′b), 2.18 (3H, s, CH_3_); ^13^C NMR (90 MHz, DMSO-d_6_) δ (ppm): 165.2, 164.9, 164.4 (3 × C=O), 162.3, 153.2, 152.4 (C-3, C-5, C-6), 145.4 (C-1′), 133.7, 133.6, 133.3, 129.3-128.6 (Ar), 103.5 (C-2′), 74.7, 67.7, 66.8 (C-3′ − C-5′), 61.2 (C-6′), 17.1 (CH_3_). ESI-HRMS positive mode (m/z): calcd for C_31_H_26_N_3_O_8_^+^ [M+H]^+^ 568.1714; C_31_H_25_N_3_NaO_8_^+^ [M+Na]^+^ 590.1534. Found: [M+H]^+^ 568.1711; [M+Na]^+^ 590.1531.

*3-(2′-Deoxy-2′-phthalimido-3′,4′,6′-tri-O-acetyl-β-d-glucopyranosyl)-1,2,4-triazin-5(4H)-one* (**9a**)

Prepared from amidrazone **5** (0.10 g, 0.21 mmol) and ethyl glyoxylate (43 µL, 0.21 mmol) according to general procedure 3. Reaction time: 2 d. Purified by column chromatography (3:2 EtOAc-hexane) to yield 55 mg (51%) of pale yellow syrup. R_f_ = 0.25 (5:2 EtOAc-hexane); [α]_D_ = +17 (c 0.21, CHCl_3_). ^1^H NMR (360 MHz, CDCl_3_) δ (ppm): 11.73 (1H, s, NH), 7.90–7.72 (5H, m, Ar, H-6), 6.07 (1H, pt, *J* = 9.8, 9.8 Hz, H-2′ or H-3′ or H-4′), 5.35 (1H, d, *J* = 9.4 Hz, H-1′), 5.23 (1H, pt, *J* = 9.8, 9.6 Hz, H-2′ or H-3′ or H-4′), 4.54 (1H, pt, *J* = 10.5, 10.5 Hz, H-2′ or H-3′ or H-4′), 4.40 (1H, dd, *J* = 12.6, 5.3 Hz, H-6′a), 4.26 (1H, dd, *J* = 12.6, 1.7 Hz, H-6′b), 4.12 (1H, ddd, *J* = 10.0, 5.3, 1.7 Hz, H-5′), 2.13, 2.09, 1.90 (3 × 3H, 3 s, CH_3_); ^13^C NMR (90 MHz, CDCl_3_) δ (ppm): 171.1, 169.8, 169.6 (CH_3_-*C*=O), 167.8 (2 × Phth-*C*=O), 161.2, 159.2 (C-3, C-5), 144.3 (C-6), 134.5, 131.3, 123.9 (Ar), 76.2, 71.4, 70.5, 68.5 (C-1′, C-3′ − C-5′), 62.1 (C-6′), 52.5 (C-2′), 20.8, 20.6, 20.4 (3 × CH_3_). ESI-HRMS positive mode (m/z): calcd for C_23_H_23_N_4_O_10_^+^ [M+H]^+^ 515.1409; C_23_H_22_N_4_NaO_10_^+^ [M+Na]^+^ 537.1228. Found: [M+H]^+^ 515.1409; [M+Na]^+^ 537.1226.

*3-(2′-Deoxy-2′-phthalimido-3′,4′,6′-tri-O-acetyl-β-d-glucopyranosyl)-6-methyl-1,2,4-triazin-5(4H)-one* (**9b**)

Prepared from amidrazone **5** (0.10 g, 0.21 mmol) and methyl pyruvate (23 µL, 0.21 mmol) according to general procedure 3. Reaction time: 2 d. Purified by column chromatography (3:2 EtOAc-hexane) to yield 56 mg (50%) of pale yellow syrup. R_f_ = 0.25 (5:2 EtOAc-hexane); [α]_D_ = +45 (c 0.21, CHCl_3_). ^1^H NMR (360 MHz, CDCl_3_) δ (ppm): 11.39 (1H, s, NH), 7.86–7.72 (4H, m, Ar), 6.06 (1H, pt, *J* = 9.8, 9.6 Hz, H-2′ or H-3′ or H-4′), 5.31 (1H, d, *J* = 10.5 Hz, H-1′), 5.23 (1H, pt, *J* = 9.8, 9.5 Hz, H-2′ or H-3′ or H-4′), 4.54 (1H, pt, *J* = 10.5, 9.8 Hz, H-2′ or H-3′ or H-4′), 4.39 (1H, dd, *J* = 12.1, 5.0 Hz, H-6′a), 4.27 (1H, dd, *J* = 12.3, < 1 Hz, H-6′b), 4.16-4-07 (1H, m, H-5′), 2.20 (3H, s, CH_3_), 2.14, 2.09, 1.89 (3 × 3H, 3 s, CH_3_); ^13^C NMR (90 MHz, CDCl_3_) δ (ppm): 171.0, 169.9, 169.6 (CH_3_-*C*=O), 167.9, 167.7 (2 × Phth-*C*=O), 161.9, 158.8, 153.8 (C-3, C-5, C-6), 134.4 (2), 131.4, 131.3, 123.8 (2) (Ar), 76.1, 71.3, 70.5, 68.5 (C-1′, C-3′ − C-5′), 62.1 (C-6′), 52.6 (C-2′), 20.8, 20.6, 20.4 (3 × CH_3_), 17.4 (CH_3_). ESI-HRMS positive mode (m/z): calcd for C_24_H_25_N_4_O_10_^+^ [M+H]^+^ 529.1565; C_24_H_24_N_4_NaO_10_^+^ [M+Na]^+^ 551.1385. Found: [M+H]^+^ 529.1569; [M+Na]^+^ 551.1386.

*((6aS,7R,7aR)-1-(2′,3′,4′,6′-Tetra-O-benzoyl-β-d-glucopyranosyl)-6,6a,7,7a,8,9-hexahydro-5H-cyclopropa[5,6]cyclookta[1,2-c]pyridin-7-yl)methanol and ((6aR,7S,7aS)-1-(2′,3′,4′,6′-tetra-O-benzoyl-β-d-glucopyranosyl)-6,6a,7,7a,8,9-hexahydro-5H-cyclopropa[5,6]cyclookta[1,2-c]pyridin-7-yl)methanol* (**11a**)

Prepared from triazine **2a** (0.10 g, 0.15 mmol) and ((1*R*,8*S*,9*r*)-bicyclo[6.1.0]non-4-yn-9-yl)methanol (**10**, 46 mg, 0.30 mmol) in CH_2_Cl_2_ according to general procedure 4. Reaction time: 2 d. Purified by column chromatography (1:1 EtOAc-hexane) to give 115 mg (97%) of colourless syrup. Diastereomeric ratio: 5:4. R_f_ = 0.15 (1:1 EtOAc-hexane). ^1^H NMR (360 MHz, CDCl_3_) δ (ppm): 8.29, 8.26 (2 × 1H, 2 d, *J* = 4.8 Hz in each, 2 × H-3), 8.02–7.21 (2 × 20H, m, Ar), 6.94, 6.92 (2 × 1H, 2 d, *J* = 4.8 Hz in each, 2 × H-4), 6.44 (2H, pt, *J* = 9.6, 9.5 Hz, 2 × (H-2′ or H-3′ or H-4′)), 6.11 (2H, pt, *J* = 9.5, 9.5 Hz, 2 × (H-2′ or H-3′ or H-4′)), 5.88, 5.87 (2 × 1H, 2 pt, *J* = 9.7, 9.6 Hz in each, 2 × (H-2′ or H-3′ or H-4′)), 5.27, 5.25 (2 × 1H, 2 d, *J* = 9.7 Hz in each, 2 × H-1′), 4.81, 4.71 (2 × 1H, 2 dd, *J* = 12.2, 2.1 Hz in each, 2 × H-6′a), 4.51, 4.46 (2 × 1H, 2 dd, *J* = 12.2, 5.1 Hz in each, 2 × H-6′b), 4.44–4.37 (2 × 1H, m, 2 × H-5′), 3.36–3.31, 3.18–2.87, 2.75–2.45, 2.38–2.33, 1.47–1.26, 0.66–0.52, 0.36–0.20 (2 × 14H, m, aliphatics, OH); ^13^C NMR (90 MHz, CDCl_3_) δ (ppm): 166.2, 166.1, 166.0, 165.9, 165.2, 165.2, 164.5, 164.4 (2 × 4 × C=O), 152.4, 152.3, 151.2, 151.1 (2 × C-1, 2 × C-4a), 146.4 (2 × C-3), 137.6, 137.4 (2 × C-9a), 133.3-132.7, 130.0-128.0 (Ar), 125.9, 125.8 (2 × C-4), 79.0, 78.3, 76.8, 76.8, 75.1, 75.1, 71.3, 71.2, 69.9, 69.8 (2 × (C-1′ − C-5′)), 66.1, 66.1 (2 × C-6′), 63.5, 63.2 (2 × CH_2_OH), 33.3, 33.2, 30.0, 29.7, 29.2, 28.5, 28.3, 28.0, 25.8, 25.4, 21.4, 20.8, 20.4, 19.9 (2 × (C-5, C-6, C-6a, C-7, C-7a, C-8, C-9)). ESI-HRMS positive mode (m/z): calcd for C_47_H_44_NO_10_^+^ [M+H]^+^: 782.2960; C_47_H_43_NNaO_10_^+^ [M+Na]^+^: 804.2779. Found: [M+H]^+^: 782.2959; [M+Na]^+^: 804.2779.

*((6aS,7R,7aR)-3-Methyl-1-(2′,3′,4′,6′-tetra-O-benzoyl-β-d-glucopyranosyl)-6,6a,7,7a,8,9-hexahydro-5H-cyclopropa[5,6]cyclookta[1,2-c]pyridin-7-yl)methanol and ((6aR,7S,7aS)-3-methyl-1-(2′,3′,4′,6′-tetra-O-benzoyl-β-d-glucopyranosyl)-6,6a,7,7a,8,9-hexahydro-5H-cyclopropa[5,6]cyclookta[1,2-c]pyridin-7-yl)methanol* (**11b**)

Prepared from triazine **2a** (0.05 g, 0.074 mmol) and ((1*R*,8*S*,9*r*)-bicyclo[6.1.0]non-4-yn-9-yl)methanol (**10**, 22 mg, 0.148 mmol) in CH_2_Cl_2_ according to general procedure 4. Reaction time: 6 d. Purified by column chromatography (4:5 EtOAc-hexane) to give 24 mg (41%) of colourless amorphous solids. Diastereomeric ratio: 5:4. R_f_ = 0.28 (1:1 EtOAc-hexane). ^1^H NMR (360 MHz, CDCl_3_) δ (ppm): 8.05-7.23 (2 × 20H, m, Ar), 6.77, 6.76 (2 × 1H, 2 s, 2 × H-4), 6.47, 6.43 (2 × 1H, 2 pt, *J* = 9.7, 9.6 Hz in each, 2 × (H-2′ or H-3′ or H-4′)), 6.07 (2H, pt, *J* = 9.6, 9.6 Hz, 2 × (H-2′ or H-3′ or H-4′)), 5.86, 5.84 (2 × 1H, 2 pt, *J* = 9.7, 9.6 Hz in each, 2 × (H-2′ or H-3′ or H-4′)), 5.17, 5.16 (2 × 1H, 2 d, *J* = 9.7 Hz in each, 2 × H-1′), 4.88, 4.70 (2 × 1H, 2 dd, *J* = 12.1, 2.8 Hz in each, 2 × H-6′a), 4.49 (1H, dd, *J* = 12.1, 5.3 Hz, H-6′b), 4.42-4.33 (3H, m, H-6′b, 2 × H-5′), 3.50-2.32 (m, aliphatics), 2.30, 2.26 (2 × 3H, 2 s, 2 × CH_3_), 1.40-0.31 (m, aliphatics, OH); ^13^C NMR (90 MHz, CDCl_3_) δ (ppm): 166.3, 166.2, 166.1, 166.0, 165.3, 165.3, 164.7, 164.6 (2 × 4 × C=O), 154.8, 154.6, 152.4, 152.3, 150.4, 150.3 (2 × C-1, 2 × C-4a, 2 × C-3), 134.4, 134.3 (2 × C-9a), 133.3-132.6, 130.1-128.0 (Ar), 125.4, 125.3 (2 × C-4), 79.6, 78.8, 76.9, 76.9, 75.3, 75.2, 71.1, 70.8, 70.0, 69.8 (2 × (C-1′ − C-5′)), 66.3, 65.7 (2 × C-6′), 63.6, 63.0 (2 × CH_2_OH), 33.1, 33.0, 30.0, 29.9, 28.8, 28.2, 28.1, 26.8, 25.5, 25.3, 23.5, 23.4, 21.6, 20.8, 20.6, 20.4 (2 × (C-5, C-6, C-6a, C-7, C-7a, C-8, C-9), 2 × CH_3_). ESI-HRMS positive mode (m/z): calcd for C_48_H_45_NNaO_10_^+^ [M+Na]^+^: 818.2936. Found: 818.2935.

*((6aS,7R,7aR)-3-Phenyl-1-(2′,3′,4′,6′-tetra-O-benzoyl-β-d-glucopyranosyl)-6,6a,7,7a,8,9-hexahydro-5H-cyclopropa[5,6]cyclookta[1,2-c]pyridin-7-yl)methanol and ((6aR,7S,7aS)-3-phenyl-1-(2′,3′,4′,6′-tetra-O-benzoyl-β-d-glucopyranosyl)-6,6a,7,7a,8,9-hexahydro-5H-cyclopropa[5,6]cyclookta[1,2-c]pyridin-7-yl)methanol* (**11d**)

Prepared from triazine **2d** (0.05 g, 0.068 mmol) and ((1*R*,8*S*,9*r*)-bicyclo[6.1.0]non-4-yn-9-yl)methanol (**10**, 20 mg, 0.136 mmol) in CH_2_Cl_2_ according to general procedure 4. Reaction time: 6 d. Purified by column chromatography (1:1 EtOAc-hexane) to give 43 mg (73%) of colourless amorphous solids. Diastereomeric ratio: 5:4. R_f_ = 0.28 (1:1 EtOAc-hexane). ^1^H NMR (360 MHz, CDCl_3_) δ (ppm): 8.04-7.15 (2 × 26H, m, Ar, 2 × H-4), 6.69, 6.65 (2 × 1H, 2 pt, *J* = 9.7, 9.7 Hz in each, 2 × (H-2′ or H-3′ or H-4′)), 6.12 (2H, pt, *J* = 9.6, 9.5 Hz, 2 × (H-2′ or H-3′ or H-4′)), 5.89, 5.86 (2 × 1H, 2 pt, *J* = 9.8, 9.6 Hz in each, 2 × (H-2′ or H-3′ or H-4′)), 5.31, 5.29 (2 × 1H, 2 d, *J* = 9.8 Hz in each, 2 × H-1′), 4.90, 4.67 (2 × 1H, 2 dd, *J* = 12.1, 2.9 Hz in each, 2 × H-6′a), 4.50 (1H, dd, *J* = 12.1, 5.3 Hz, H-6′b), 4.42–4.36 (3 × 1H, m, H-6′b, 2 × H-5′), 3.43–3.38, 3.20–2.52, 2.46–2.34, 1.52–1.36, 0.74–0.34 (2 × 13H, m, aliphatics); ^13^C NMR (90 MHz, CDCl_3_) δ (ppm): 166.3, 166.3, 166.2, 166.1, 165.3, 165.2, 164.8, 164.6 (2 × 4 × C=O), 154.0, 153.6, 153.0, 153.0, 150.9, 150.8 (2 × C-1, 2 × C-3, 2 × C-4a), 138.9, 138.7, 136.4, 136.1 (2 × C-9a, 2 × Ar-C_q_), 133.4-132.5, 129.9-126.8 (Ar), 122.6, 122.3 (2 × C-4), 79.0, 78.4, 76.9, 76.9, 75.6, 75.4, 70.8, 70.5, 69.9, 69.6 (2 × (C-1′ − C-5′)), 66.2, 66.2 (2 × C-6′), 63.7, 62.9 (2 × CH_2_OH), 33.5, 30.0, 29.9, 29.1, 28.8, 28.2, 28.0, 25.6, 25.4, 20.5, 20.5, 20.4, 20.3 (2 × (C-5, C-6, C-6a, C-7, C-7a, C-8, C-9)). ESI-HRMS positive mode (m/z): calcd for C_53_H_48_NO_10_^+^ [M+H]^+^: 858.3273; C_53_H_47_NNaO_10_^+^ [M+Na]^+^: 880.3092. Found: [M+H]^+^: 858.3272; [M+Na]^+^: 880.3093.

*((6aS,7R,7aR)-1-(β-d-glucopyranosyl)-3-phenyl-6,6a,7,7a,8,9-hexahydro-5H-cyclopropa[5,6]cyclookta[1,2-c]pyridin-7-yl)methanol and ((6aR,7S,7aS)-1-(β-d-glucopyranosyl)-3-phenyl-6,6a,7,7a,8,9-hexahydro-5H-cyclopropa[5,6]cyclookta[1,2-c]pyridin-7-yl)methanol* (**12d**)

Prepared from triazine **3d** (20 mg, 0.063 mmol) and ((1*R*,8*S*,9*r*)-bicyclo[6.1.0]non-4-yn-9-yl)methanol (**10**, 19 mg, 0.126 mmol) in MeOH according to general procedure 4. Reaction time: 5 d. Purified by column chromatography (8:1 CHCl_3_-MeOH) to give 26 mg (94%) of pale yellow amorphous solids. Diastereomeric ratio: 1:1. R_f_ = 0.21 (8:1 CHCl_3_-MeOH). ^1^H NMR (360 MHz, CD_3_OD) δ (ppm): 8.02–8.01 (2 × 2H, Ph), 7.58 (2H, s, 2 × H-4), 7.47–7.36 (2 × 3H, m, Ph), 4.65, 4.63 (2 × 1H, 2 d, *J* = 9.3 Hz in each, H-1′), 4.34, 4.33 (2 × 1H, 2 pt, *J* = 9.1, 9.1 Hz in each, 2 × (H-2′ or H-3′ or H-4′)), 3.87–3.51 (2 × 4H, m, 2 × (H-2′ and/or H-3′ and/or H-4′, H-6′a, H6′b), 4.46–3.30 (2H, m, 2 × H-5′), 3.30–3.22, 3.14–3.05, 2.99–2.89, 2.65–2.44, 1.51–1.39, 0.72–0.61 (2 × 13H, m, aliphatics); ^13^C NMR (90 MHz, CD_3_OD) δ (ppm): 155.5, 155.3, 155.3, 155.2, 155.2, 155.1 (2 × C-1, 2 × C-3, 2 × C-4a), 140.8, 140.7, 138.5, 138.5 (2 × C-9a, 2 × Ph-C_q_), 129.8, 129.7, 129.6, 129.6, 128.0 (Ph), 123.3, 123.2 (2 × C-4), 82.4, 82.4, 79.7, 79.7, 79.6, 73.8, 73.7, 71.6, 71.5 (2 × (C-1′ − C-5′)), 66.6, 66.5 (2 × C-6′), 62.9, 62.8 (2 × CH_2_OH), 35.0, 34.8, 31.1, 31.0, 30.3, 30.1, 29.9, 29.8, 26.6, 26.5, 23.0, 22.9, 22.6, 22.5 (2 × (C-5, C-6, C-6a, C-7, C-7a, C-8, C-9)). ESI-HRMS positive mode (m/z): calcd for C_25_H_31_NNaO_6_^+^ [M+Na]^+^: 464.2044. Found: 464.2042 (Supplementary Materials).

## 4. Conclusions

The reactions of *C*-glycopyranosyl formamidrazones with 1,2-dielectrophiles (α-keto-aldehydes and esters as well as 1,2-diketones) represent a simple method for the synthesis of hitherto unknown 3-glycopyranosyl-1,2,4-triazines and -1,2,4-triazin-5(4*H*)-ones. The *C*-glycosyl 1,2,4-triazines can be transformed into the corresponding 2-glycopyranosyl pyridines in strain-promoted inverse electron demand Diels–Alder reactions. These new compounds may be interesting for yet unknown applications to be explored in the future.

## Figures and Tables

**Table 1:** Synthesis of 3-(β-d-glucopyranosyl)-1,2,4-triazines.

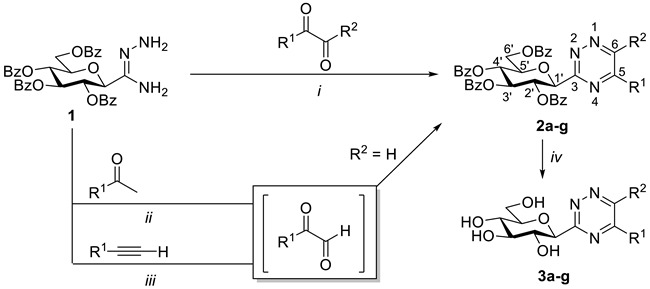 (*i*) dry EtOH, reflux; (*ii*) SeO_2_, DMSO, 110 °C; (*iii*) NIS, cat. TsOH, DMSO, 110 °C; (*iv*) NaOMe/MeOH, r.t.							
				**Conditions and Yields (%)**			
**Entry**		**R^1^**	**R^2^**		**2**		**3**
1	**a**	H	H	*i*	90	*iv*	63
2	**b**	CH_3_	H	*i*	84	*iv*	58
3	**c**	(CH_3_)_3_	H	*i*	83	*iv*	88
4	**d**	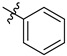	H	*i*	83	*iv*	72
5				*ii*	50		
6				*iii*	60		
7	**e**	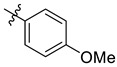	H	*i*	97	*iv*	73
8	**f**	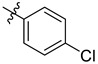	H	*i*	90	*iv*	72
9	**g**	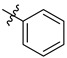	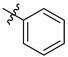	*i*	53	*iv*	84

**Table 2:** Synthesis of 3-(2′-deoxy-2′-phthalimido-3′,4′,6′-tri-*O*-acetyl-β-d-glucopyranosyl)-1,2,4-triazines.

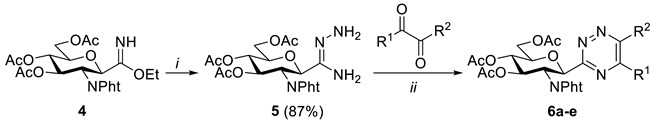 (*i*) NH_2_NH_2_^.^H_2_O, EtOH, r.t.; (*ii*) dry EtOH, reflux			
	**R^1^**	**R^2^**	**Yield of 6 (%)**
**a**	H	H	58
**b**	CH_3_	H	62
**c**	(CH_3_)_3_	H	67
**d**	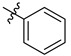	H	73
**e**	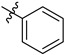	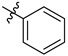	65

**Table 3:** Synthesis of 3-glycopyranosyl-1,2,4-triazin-5(4*H*)-ones.

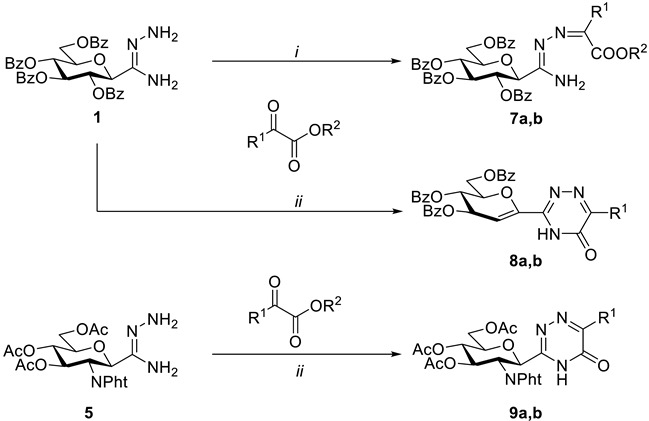 (*i*) dry EtOH, reflux; (*ii*) dry toluene, reflux			
**Product**	**R^1^**	**R^2^**	**Yield (%)**
**7a**	H	CH_2_CH_3_	39
**7b**	CH_3_	CH_3_	59
**8a**	H	-	57
**8b**	CH_3_	-	75
**9a**	H	-	51
**9b**	CH_3_	-	50

**Table 4:** IEDDA reactions of some 3-glucopyranosyl-1,2,4-triazines with a bicyclononyne derivative.

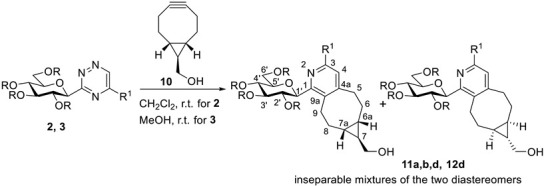						
**Triazine**	**R**	**R^1^**	**Reaction Time**	**Product**	**Yield (%)**	**Diastereomeric Ratio**
**2a**	Bz	H	2 d	**11a**	97	5:4
**2b**	Bz	CH_3_	6 d	**11b**	41	5:4
**2d**	Bz	Ph	6 d	**11d**	73	5:4
**3d**	H	Ph	5 d	**12d**	94	1:1

## Data Availability

Not applicable.

## Supplementary Materials

The following supporting information can be downloaded at: https://www.mdpi.com/article/10.3390/molecules27227801/s1, Copies of the ^1^H and ^13^C NMR spectra of the synthesised compounds.
