# Supplementary material for: First Synthesis of 3-Glycopyranosyl-1,2,4-Triazines and Some Cycloadditions Thereof
Source: Molecules. 2022 Nov 12;27(22):7801. doi: 10.3390/molecules27227801 (PMC9692545; doi:10.3390/molecules27227801)
Supplement: Supplementary file 1 [file molecules-27-07801-s001.zip › molecules-2028178-supplementary.pdf]

# SUPPORTING INFORMATION

## First synthesis of 3-glycopyranosyl-1,2,4-triazines and some cycloadditions thereof

Éva Bokor,* Attila Ferenczi, Mahir Hashimov, Éva Juhász-Tóth, Zsófia Götz, Alshimaa Ibrahim Zaki, László Somsák*

*University of Debrecen, Department of Organic Chemistry, Debrecen, Hungary*

Copies of NMR spectra of the synthesized compounds.

* Corresponding authors: É. Bokor - bokor.eva@science.unideb.hu ;
L. Somsák - somsak.laszlo@science.unideb.hu

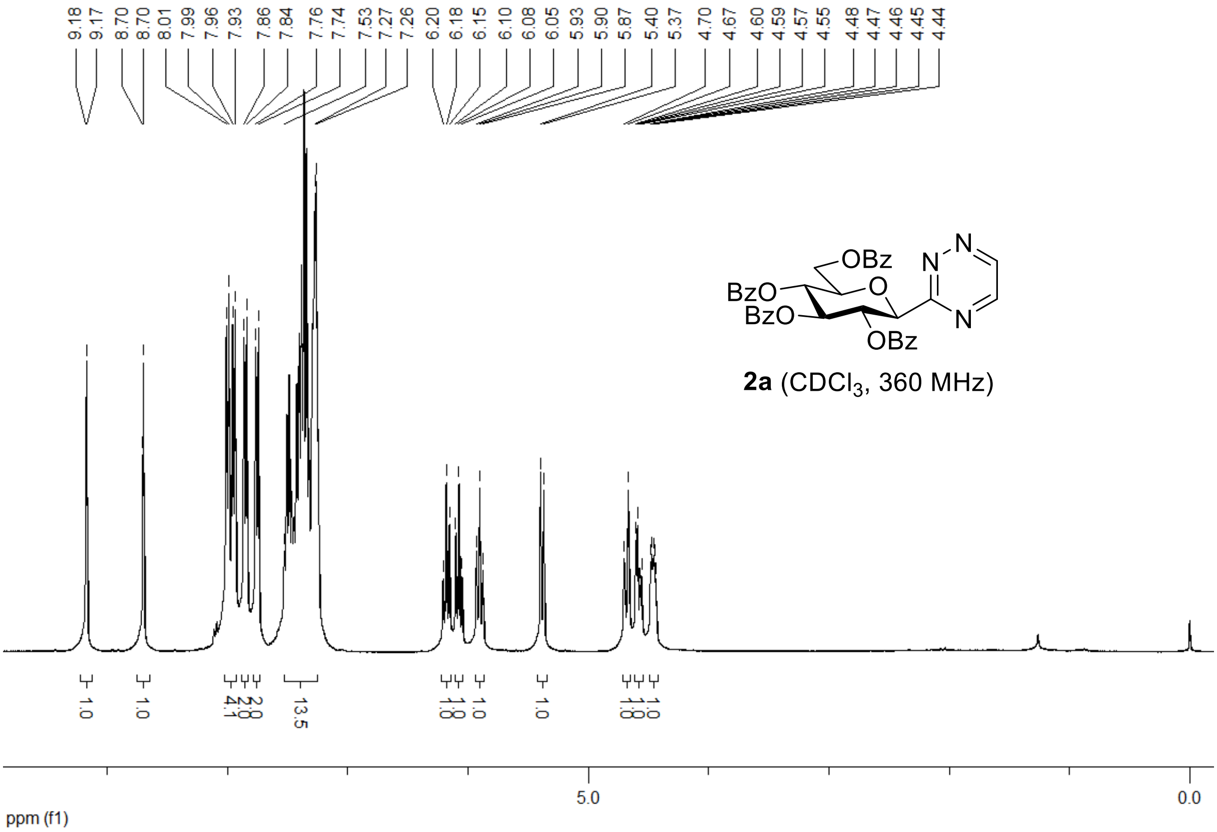


**2a** ($CDCl_3$, 360 MHz)

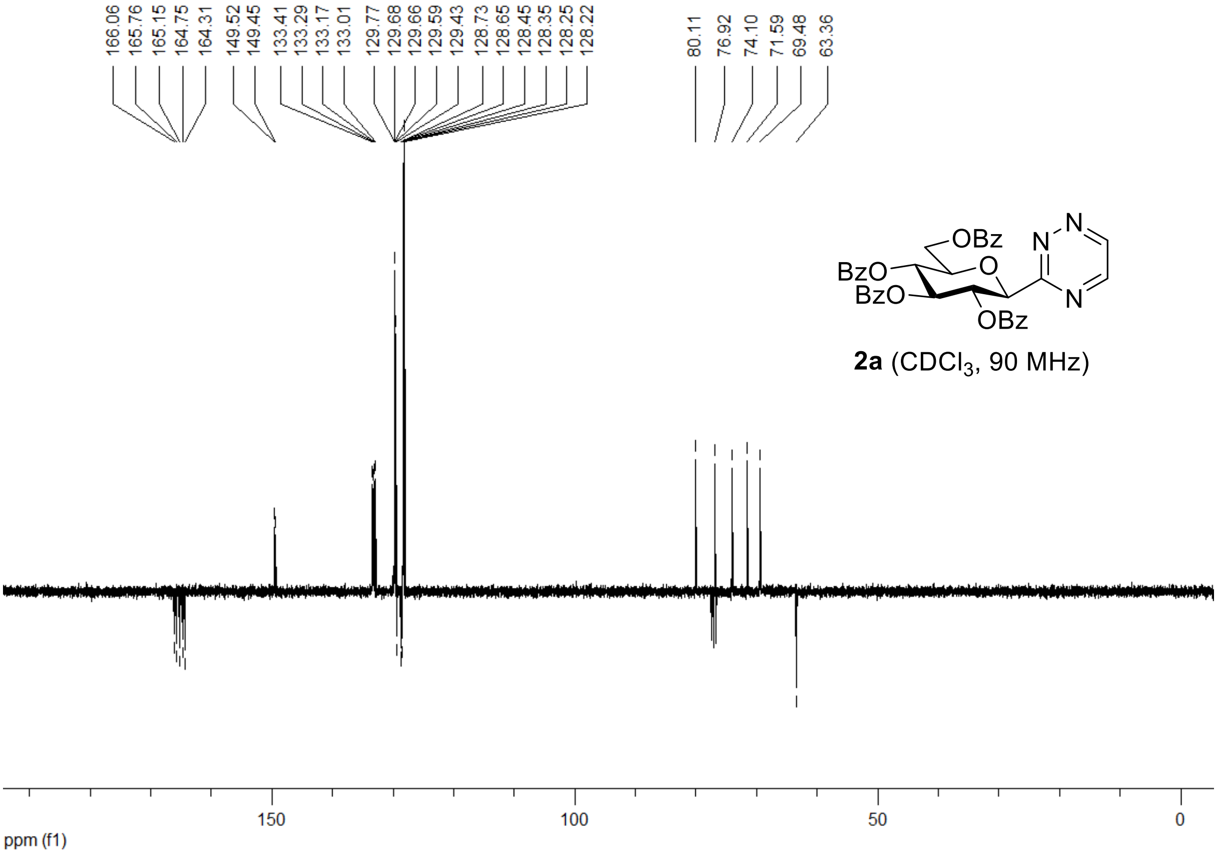


**2a** ($CDCl_3$, 90 MHz)

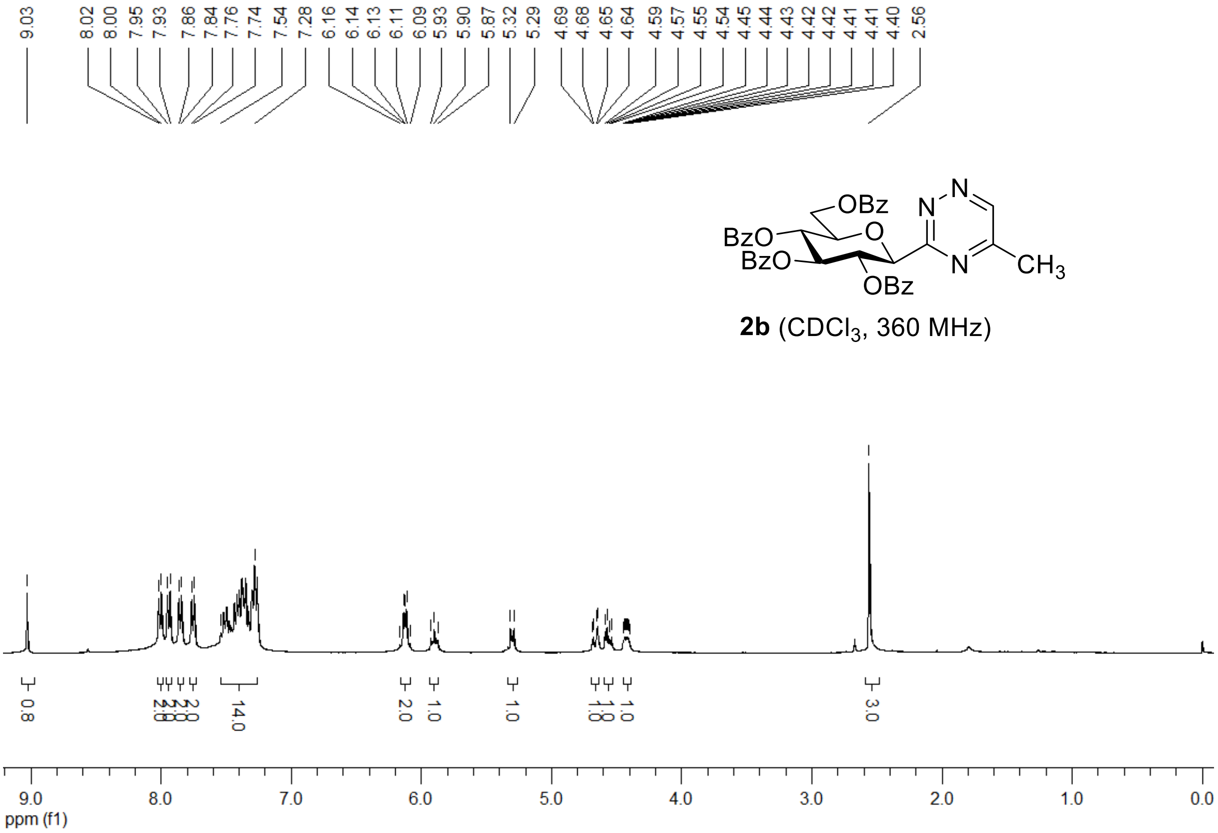


**2b** ($CDCl_3$, 360 MHz)

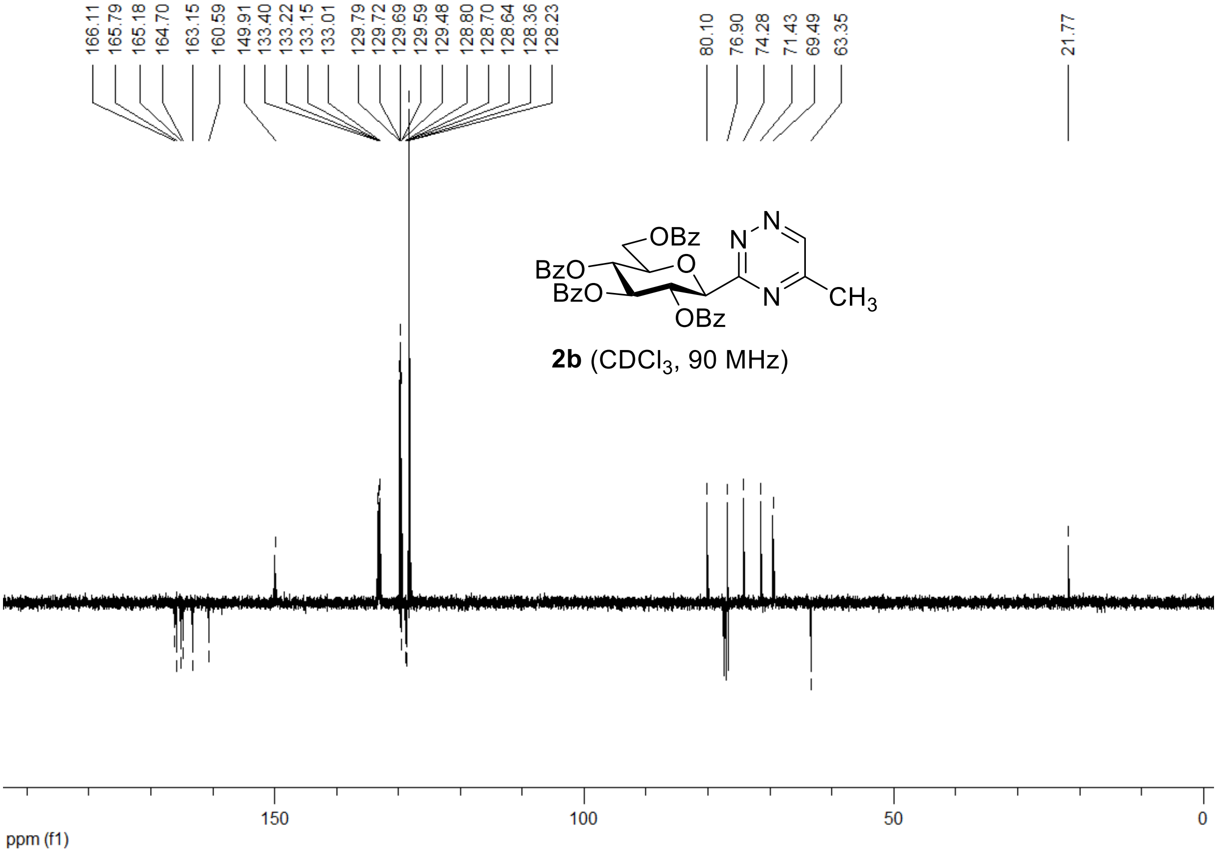


**2b** ($CDCl_3$, 90 MHz)

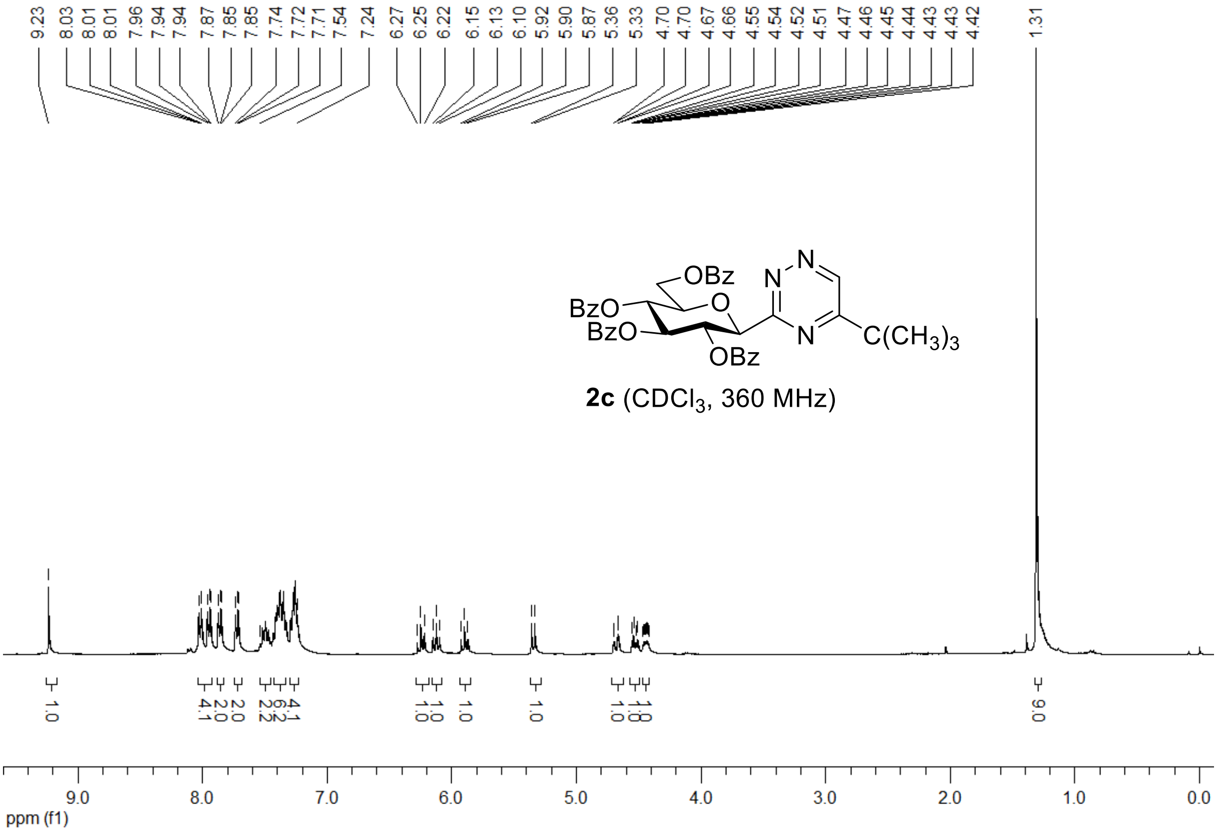
9.23 8.03 8.01 8.01 7.96 7.94 7.94 7.87 7.85 7.85 7.74 7.72 7.71 7.54 7.24 6.27 6.25 6.22 6.15 6.13 6.10 5.92 5.90 5.87 5.36 5.33 4.70 4.70 4.67 4.66 4.55 4.54 4.52 4.51 4.47 4.46 4.45 4.44 4.43 4.43 4.42 1.31
OBz
BzO
BzO
O
OBz
N
N
N
C(CH3)3
2c (CDCl3, 360 MHz)
1.0 4.1 2.0 2.0 2.2 6.2 4.1 1.0 1.0 1.0 1.0 1.0 1.0 1.8 9.0
9.0 8.0 7.0 6.0 5.0 4.0 3.0 2.0 1.0 0.0
ppm (f1)

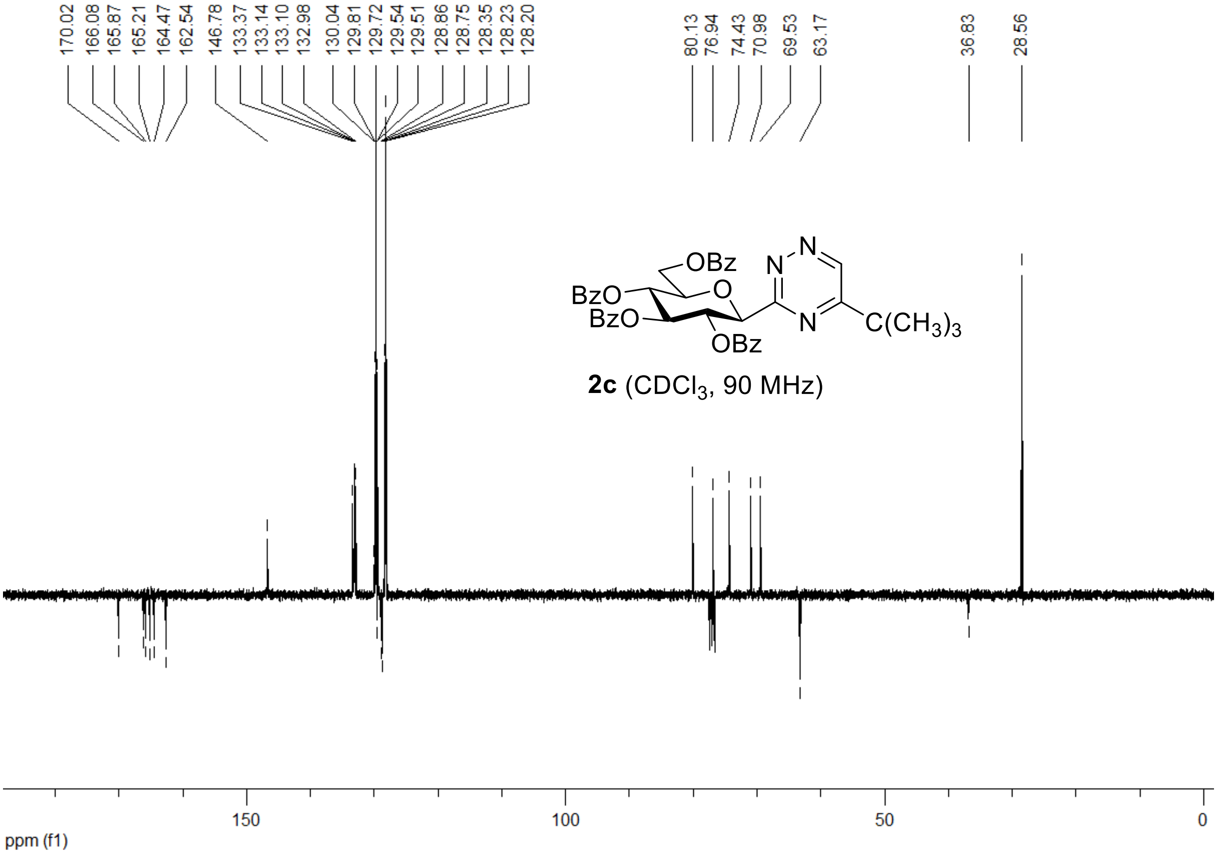
170.02 166.08 165.87 165.21 164.47 162.54 146.78 133.37 133.14 133.10 132.98 130.04 129.81 129.72 129.54 129.51 128.86 128.75 128.35 128.23 128.20 80.13 76.94 74.43 70.98 69.53 63.17 36.83 28.56
OBz
BzO
BzO
O
OBz
N
N
N
C(CH3)3
2c (CDCl3, 90 MHz)
150 100 50 0
ppm (f1)

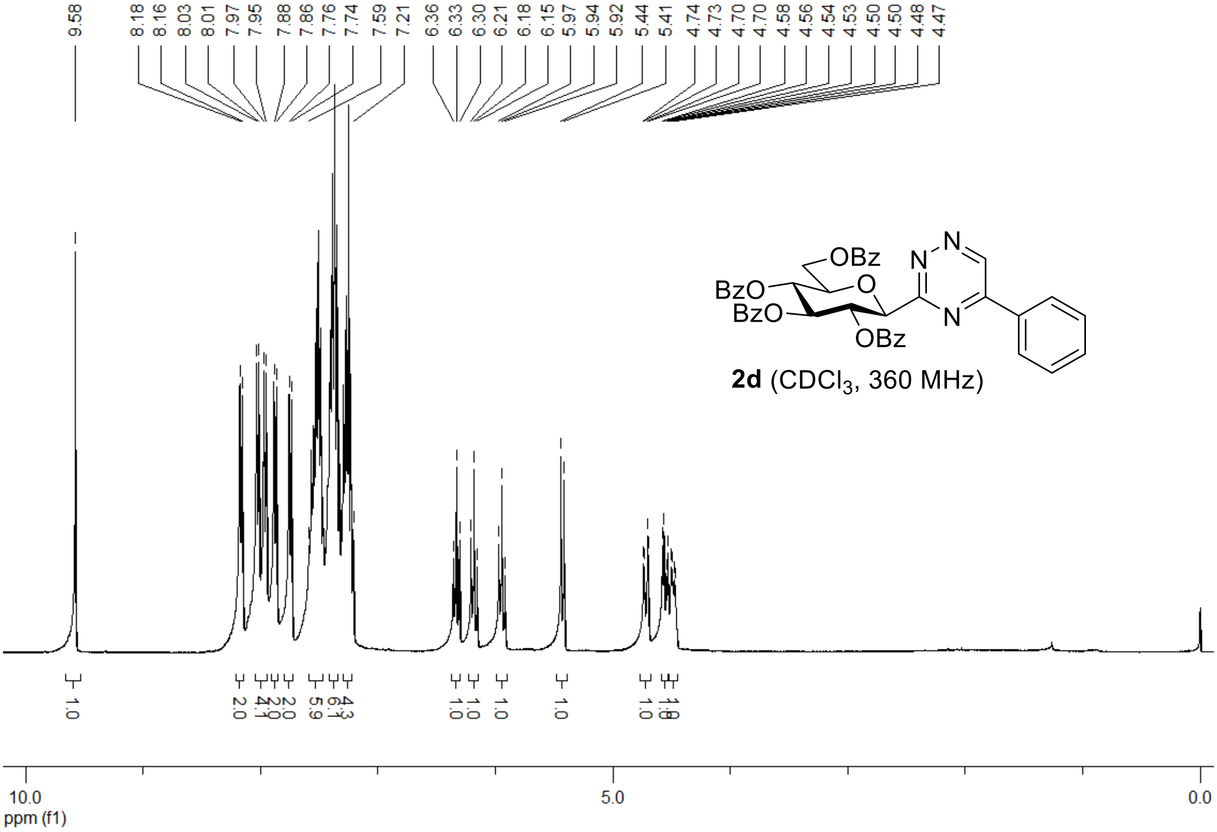
9.58
8.18 8.16 8.03 8.01 7.97 7.95 7.88 7.86 7.76 7.74 7.59 7.21
6.36 6.33 6.30 6.21 6.18 6.15 5.97 5.94 5.92 5.44 5.41 4.74 4.73 4.70 4.70 4.58 4.56 4.54 4.53 4.50 4.50 4.48 4.47
BzO
BzO
OBz
OBz
O
N
N
N
2d (CDCl3, 360 MHz)
1.0 2.0 4.1 2.0 2.0 5.9 6.1 4.3 1.0 1.0 1.0 1.0 1.0
10.0
5.0
0.0
ppm (f1)

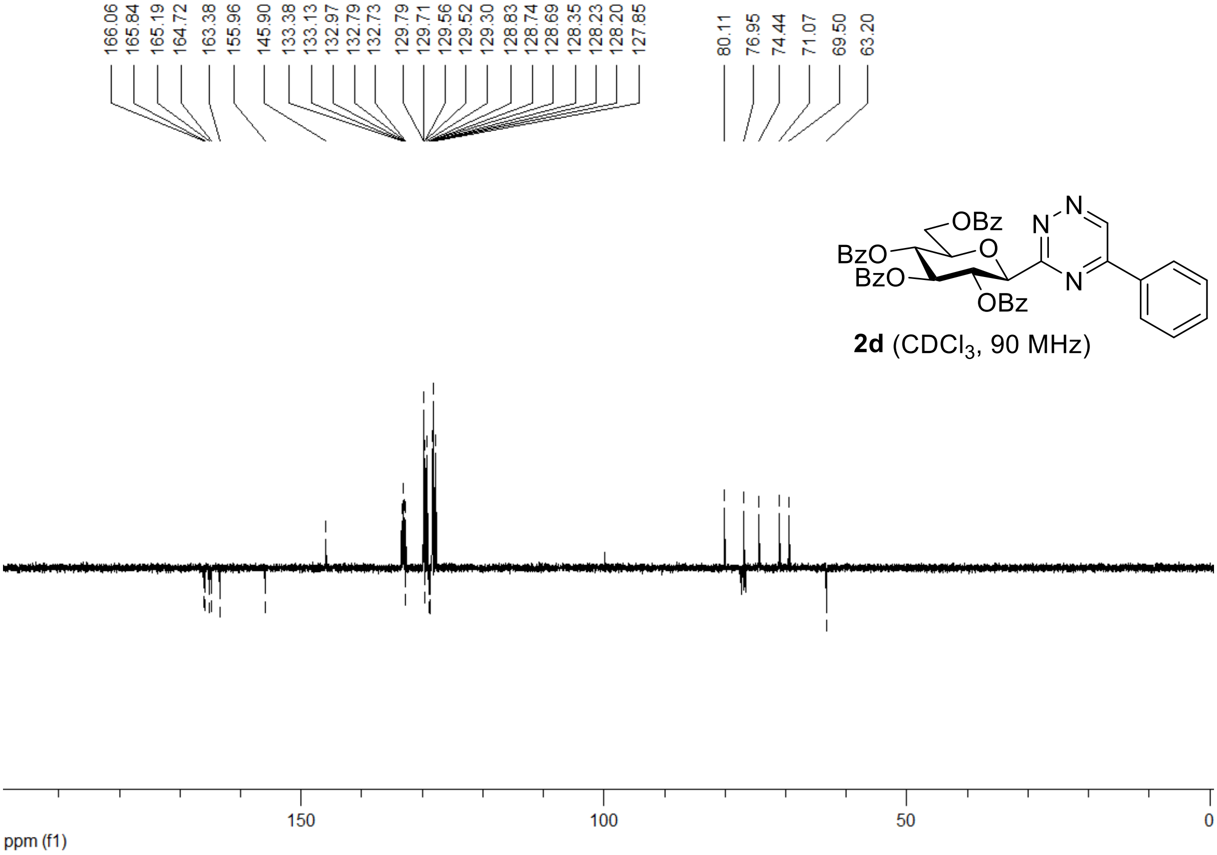
166.06 165.84 165.19 164.72 163.38 155.96 145.90 133.38 133.13 132.97 132.79 132.73 129.79 129.71 129.56 129.52 129.30 128.83 128.74 128.69 128.35 128.23 128.20 127.85
80.11 76.95 74.44 71.07 69.50 63.20
BzO
BzO
OBz
OBz
O
N
N
N
2d (CDCl3, 90 MHz)
150
100
50
0
ppm (f1)

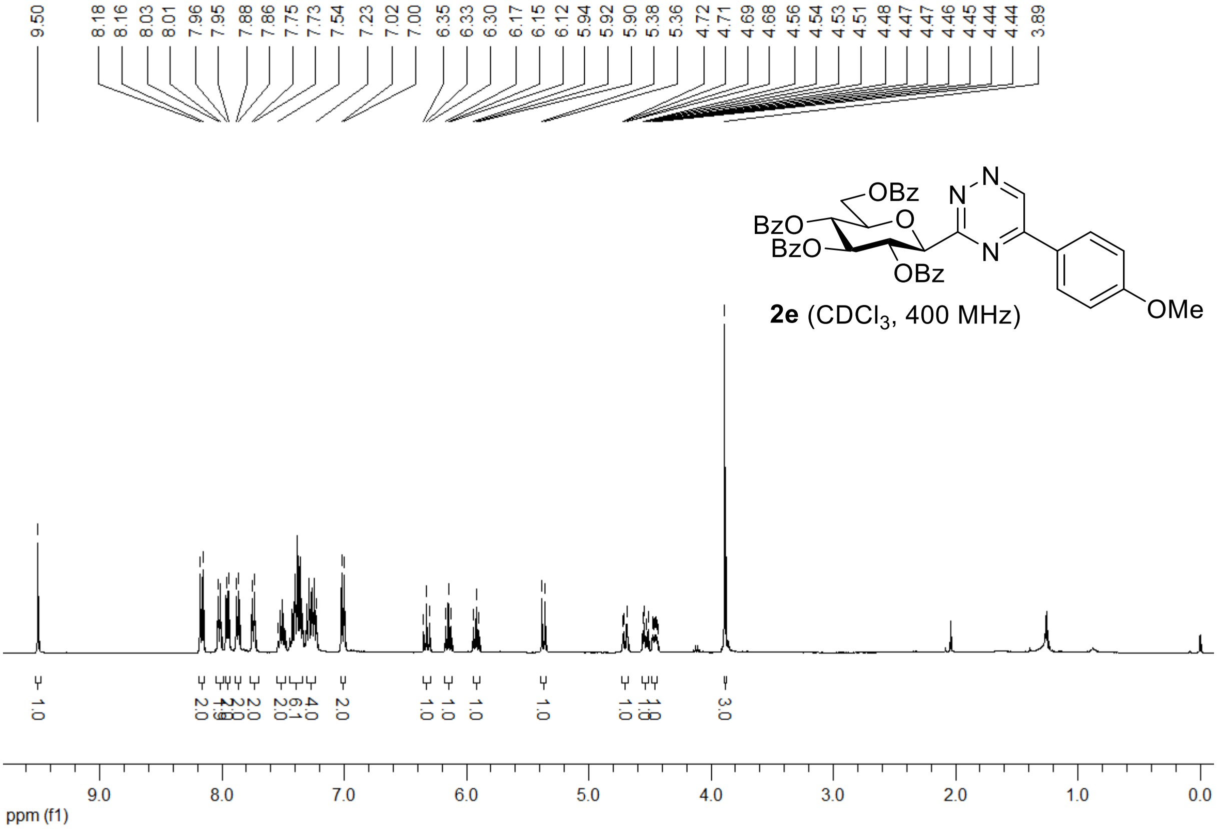
9.50 8.18 8.16 8.03 8.01 7.96 7.95 7.88 7.86 7.75 7.73 7.54 7.23 7.02 7.00 6.35 6.33 6.30 6.17 6.15 6.12 5.94 5.92 5.90 5.38 5.36 4.72 4.71 4.69 4.68 4.56 4.54 4.53 4.51 4.48 4.47 4.46 4.45 4.44 4.44 3.89
OBz
BzO
BzO
O
OBz
N
N
N
OMe
2e (CDCl3, 400 MHz)
ppm (f1)

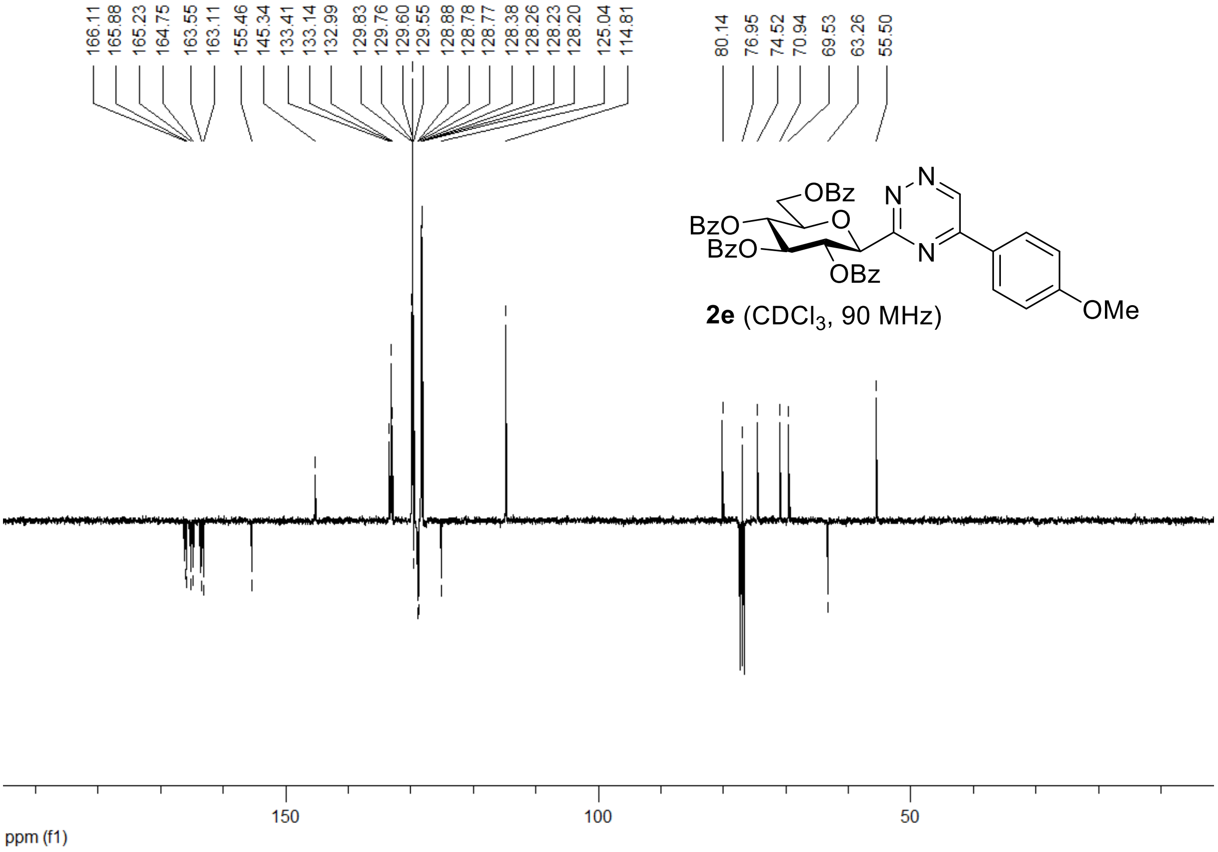
166.11 165.88 165.23 164.75 163.55 163.11 155.46 145.34 133.41 133.14 132.99 129.83 129.76 129.60 129.55 128.88 128.78 128.77 128.38 128.26 128.23 128.20 125.04 114.81
80.14 76.95 74.52 70.94 69.53 63.26 55.50
OBz
BzO
BzO
O
OBz
N
N
N
OMe
2e (CDCl3, 90 MHz)
150
100
50
ppm (f1)

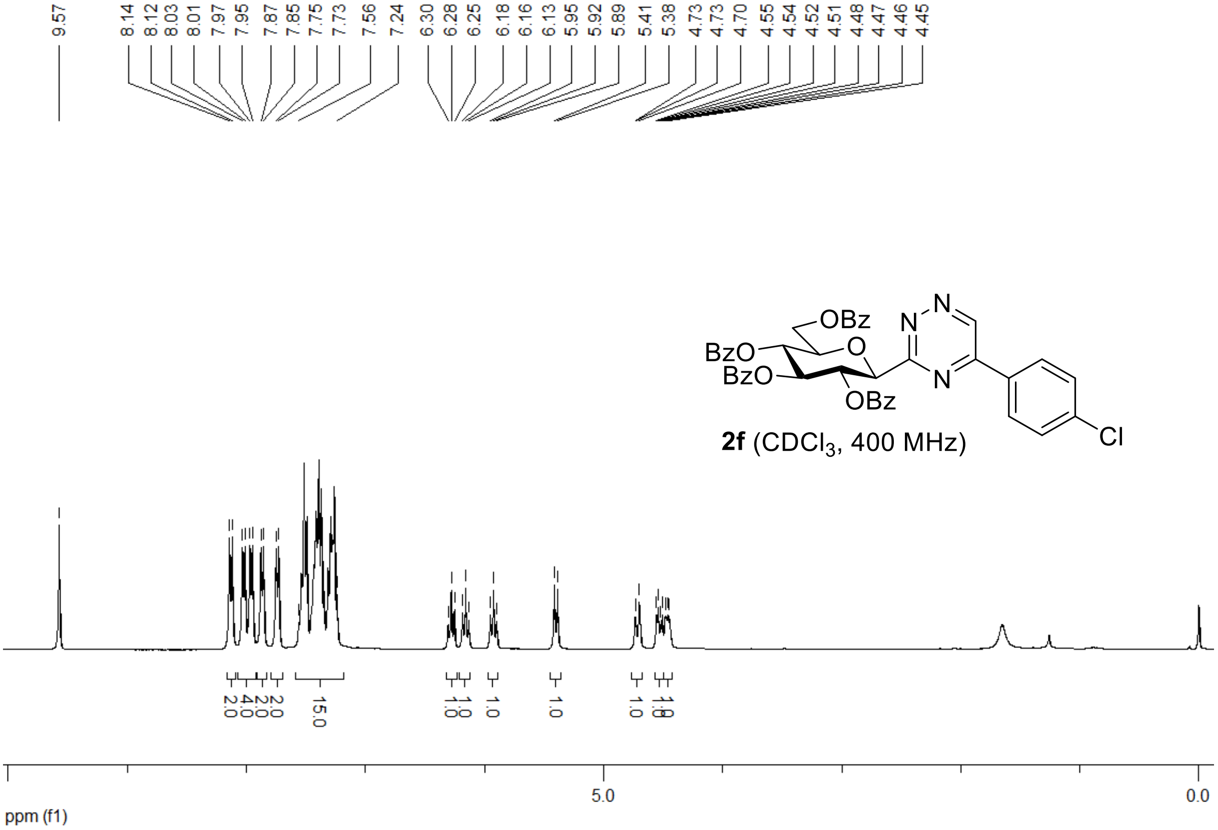
9.57
8.14 8.12 8.03 8.01 7.97 7.95 7.87 7.85 7.75 7.73 7.56 7.24
6.30 6.28 6.25 6.18 6.16 6.13 5.95 5.92 5.89 5.41 5.38 4.73 4.73 4.70 4.55 4.54 4.52 4.51 4.48 4.47 4.46 4.45
OBz
BzO
BzO
O
OBz
N
N
N
Cl
2f (CDCl3, 400 MHz)
2.0 4.0 2.0 2.0 15.0 1.0 1.0 1.0 1.0 1.0 1.0 1.0
5.0
0.0
ppm (f1)

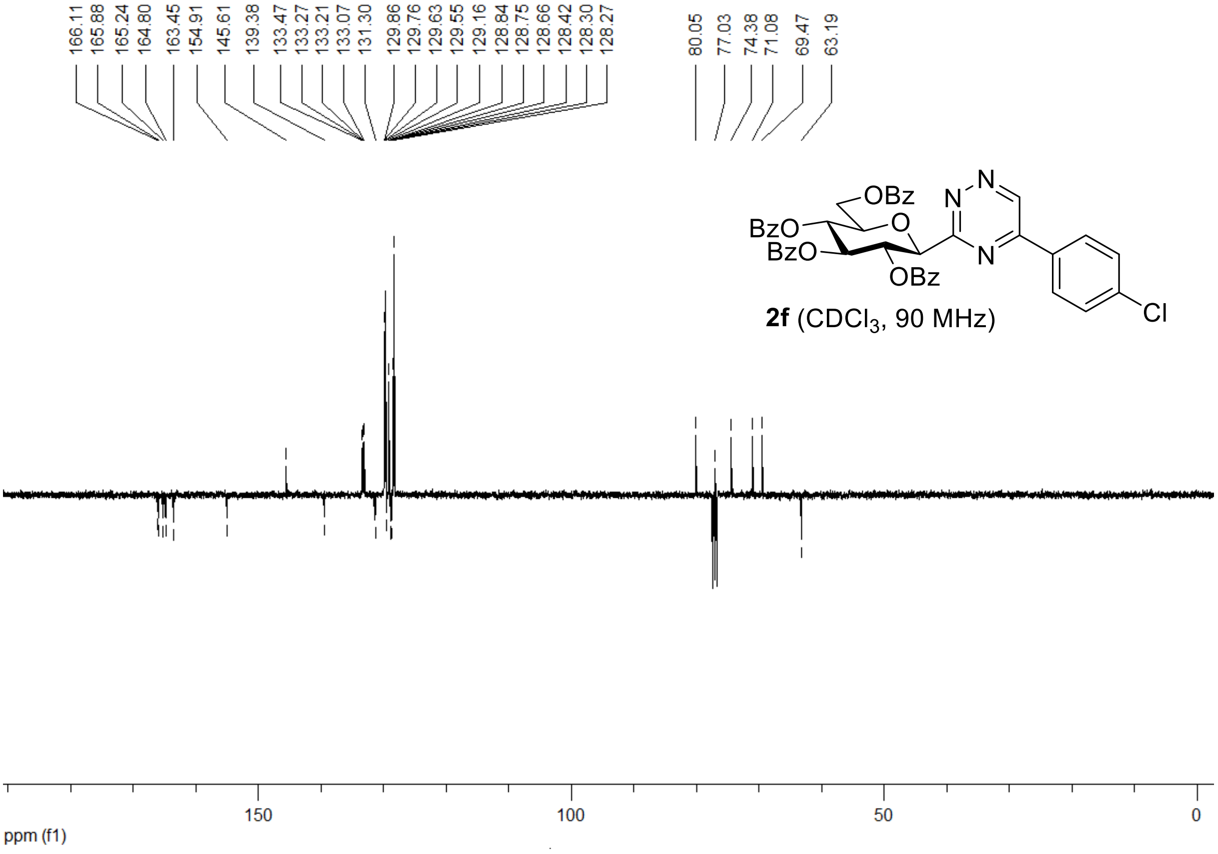
166.11 165.88 165.24 164.80 163.45 154.91 145.61 139.38 133.47 133.27 133.21 133.07 131.30 129.86 129.76 129.63 129.55 129.16 128.84 128.75 128.66 128.42 128.30 128.27
80.05 77.03 74.38 71.08 69.47 63.19
OBz
BzO
BzO
O
OBz
N
N
N
Cl
2f (CDCl3, 90 MHz)
150
100
50
0
ppm (f1)

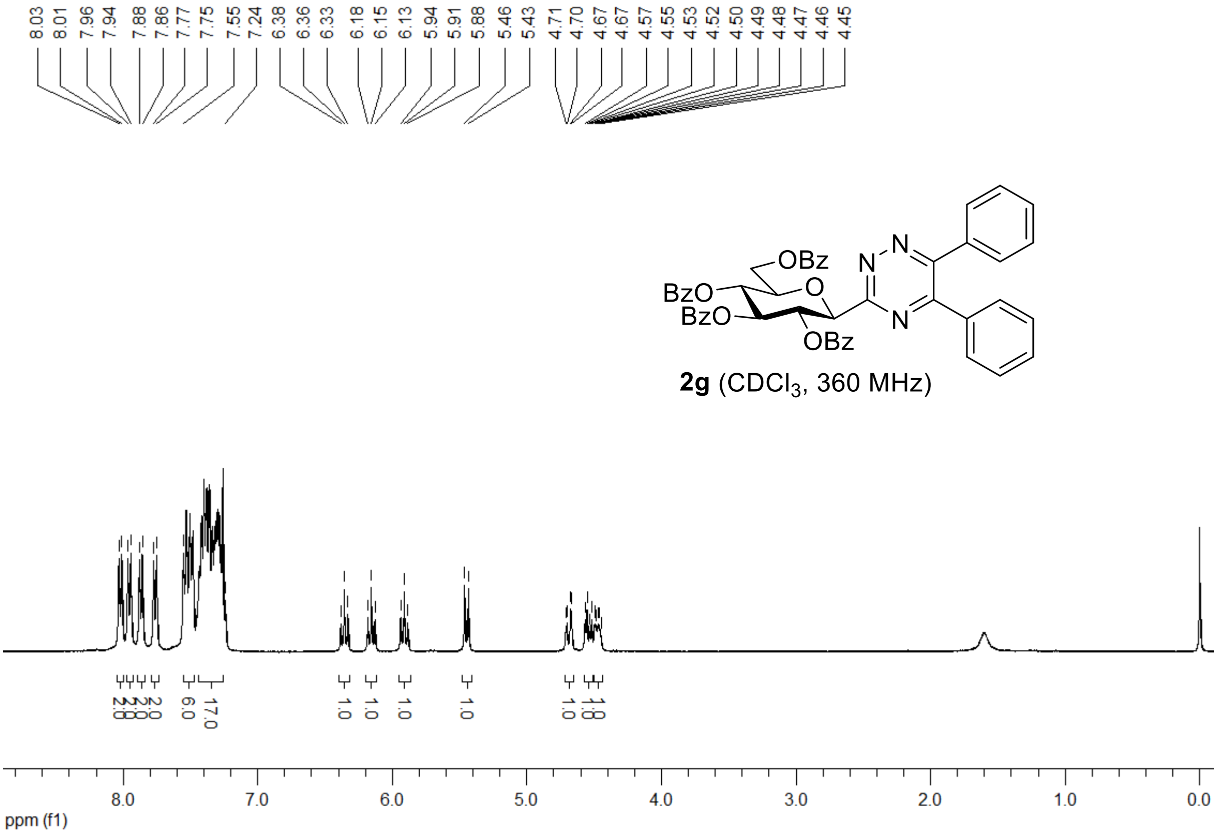
8.03 8.01 7.96 7.94 7.88 7.86 7.77 7.75 7.55 7.24 6.38 6.36 6.33 6.18 6.15 6.13 5.94 5.91 5.88 5.46 5.43 4.71 4.70 4.67 4.67 4.57 4.55 4.53 4.52 4.50 4.49 4.48 4.47 4.46 4.45
BzO
BzO
OBz
O
OBz
N
N
N
2g (CDCl3, 360 MHz)
2.8 2.8 2.0 6.0 17.0 1.0 1.0 1.0 1.0 1.0 1.8
8.0 7.0 6.0 5.0 4.0 3.0 2.0 1.0 0.0
ppm (f1)

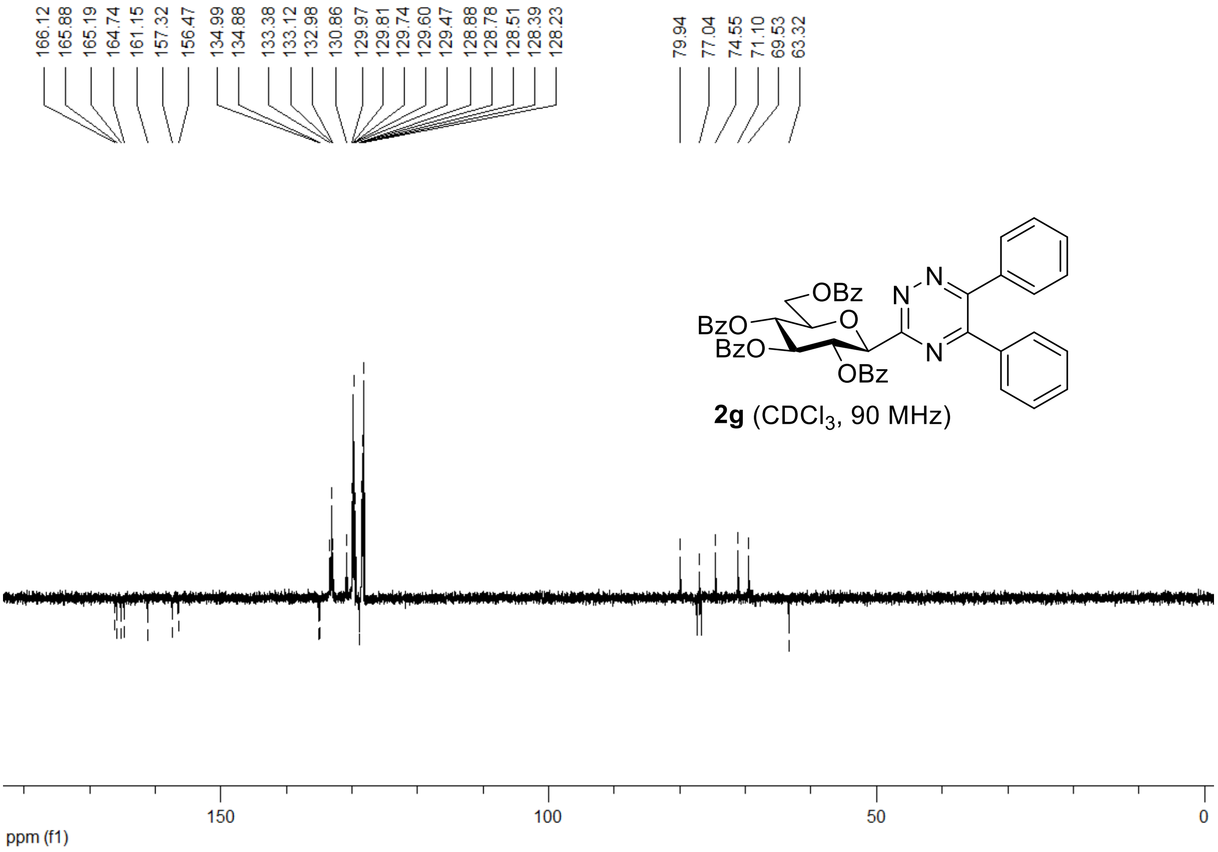
166.12 165.88 165.19 164.74 161.15 157.32 156.47 134.99 134.88 133.38 133.12 132.98 130.86 129.97 129.81 129.74 129.60 129.47 128.88 128.78 128.51 128.39 128.23
79.94 77.04 74.55 71.10 69.53 63.32
BzO
BzO
OBz
O
OBz
N
N
N
2g (CDCl3, 90 MHz)
150 100 50 0
ppm (f1)

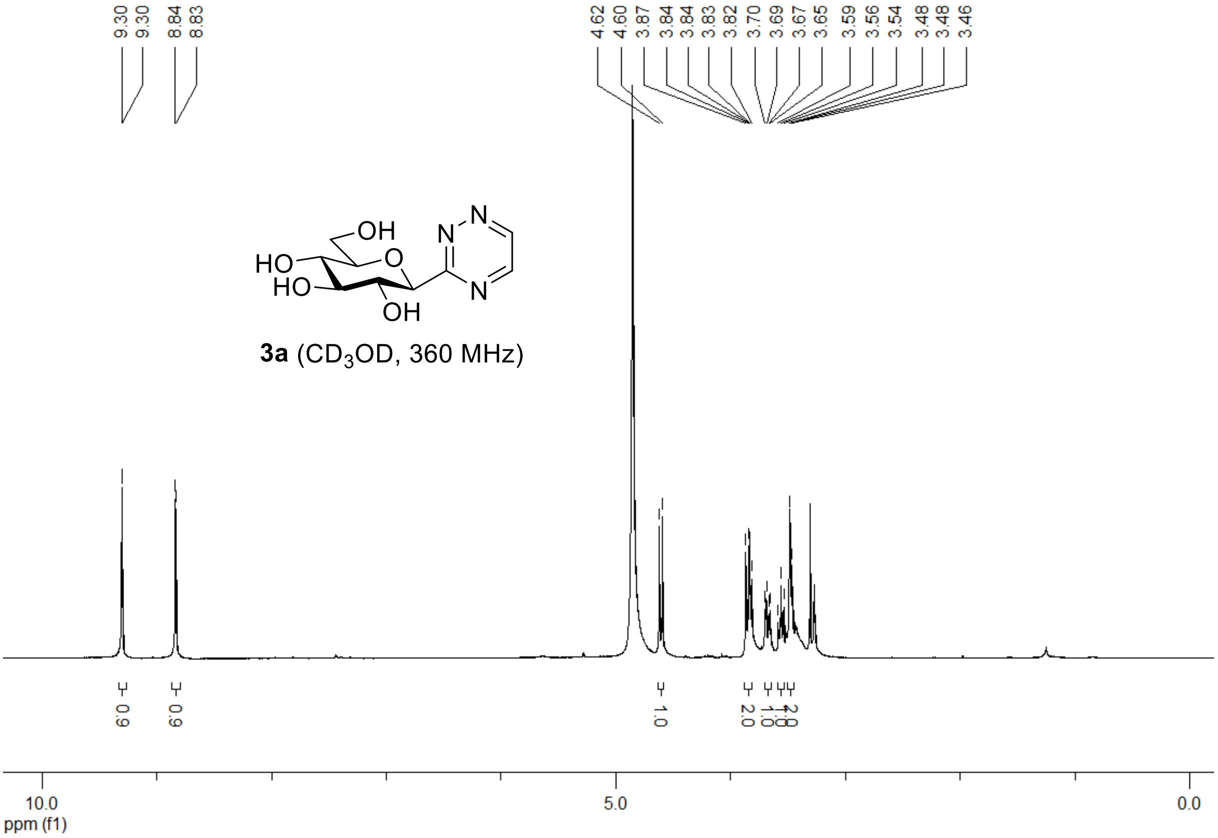
9.30
9.30
8.84
8.83
4.62
4.60
3.87
3.84
3.84
3.83
3.82
3.70
3.69
3.67
3.65
3.59
3.56
3.54
3.48
3.48
3.46
HO
HO
OH
O
OH
N
N
N
3a (CD3OD, 360 MHz)
0.9
0.9
1.0
2.0
1.0
2.0
10.0
5.0
0.0
ppm (f1)

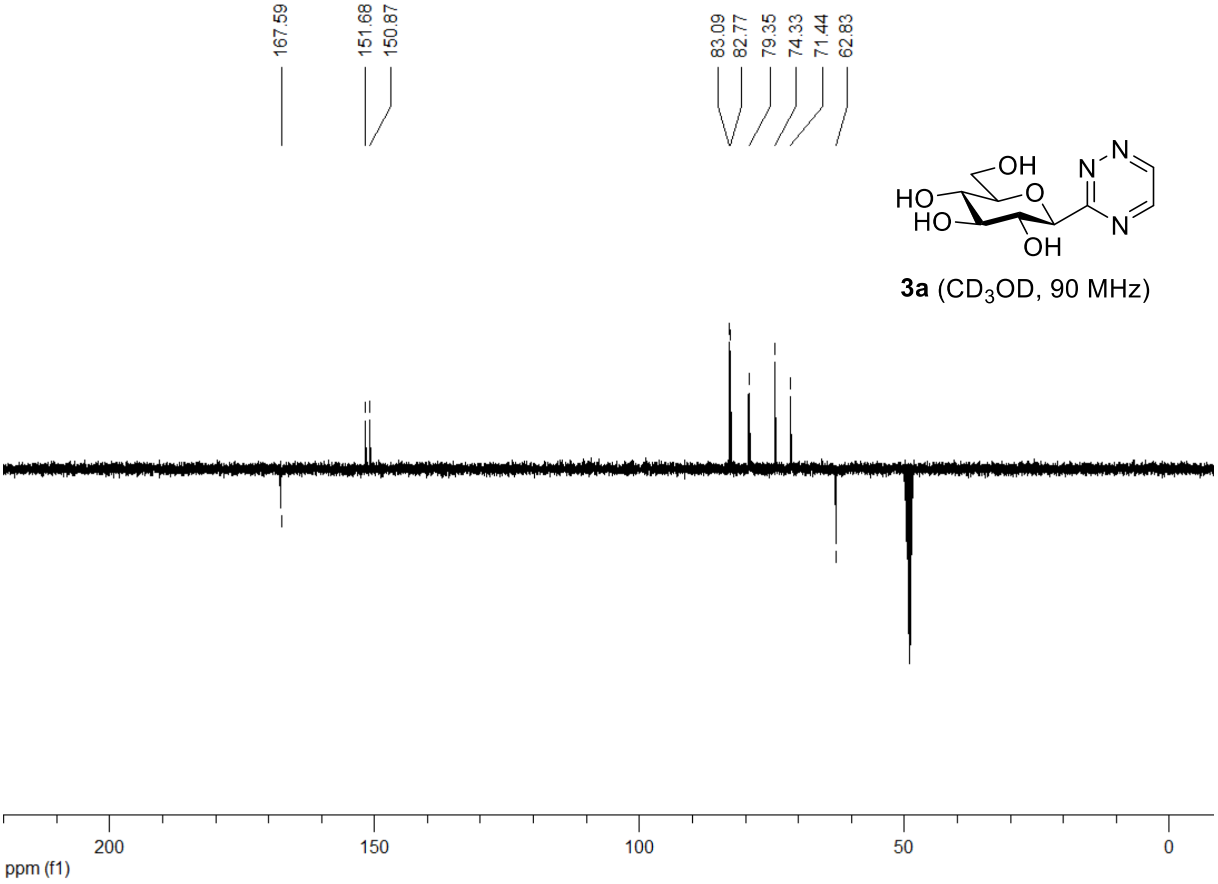
167.59
151.68
150.87
83.09
82.77
79.35
74.33
71.44
62.83
HO
HO
OH
O
OH
N
N
N
3a (CD3OD, 90 MHz)
200
150
100
50
0
ppm (f1)

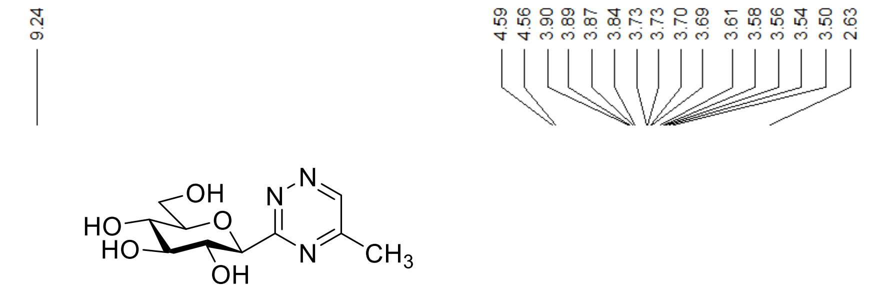


**3b** ($CD_3OD$, 360 MHz)

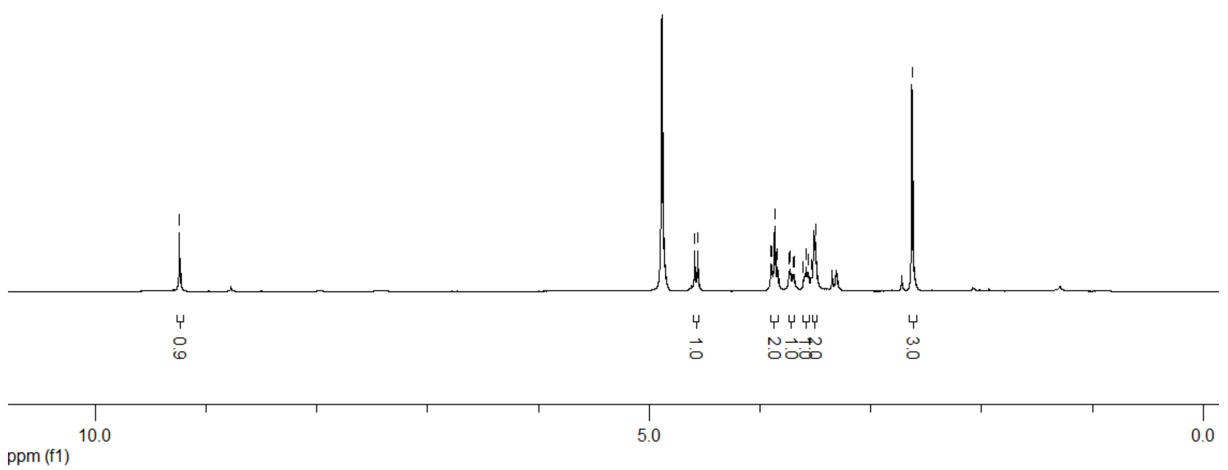


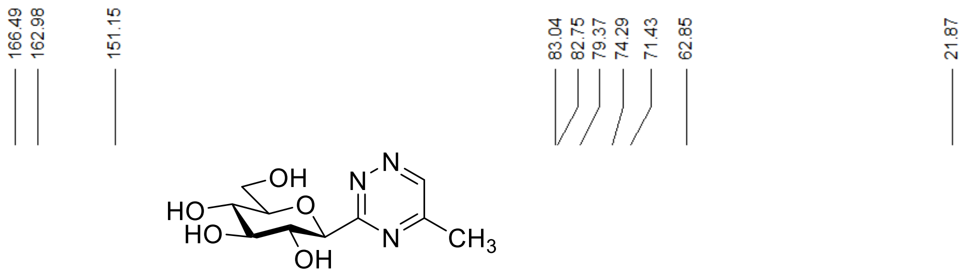


**3b** ($CD_3OD$, 90 MHz)

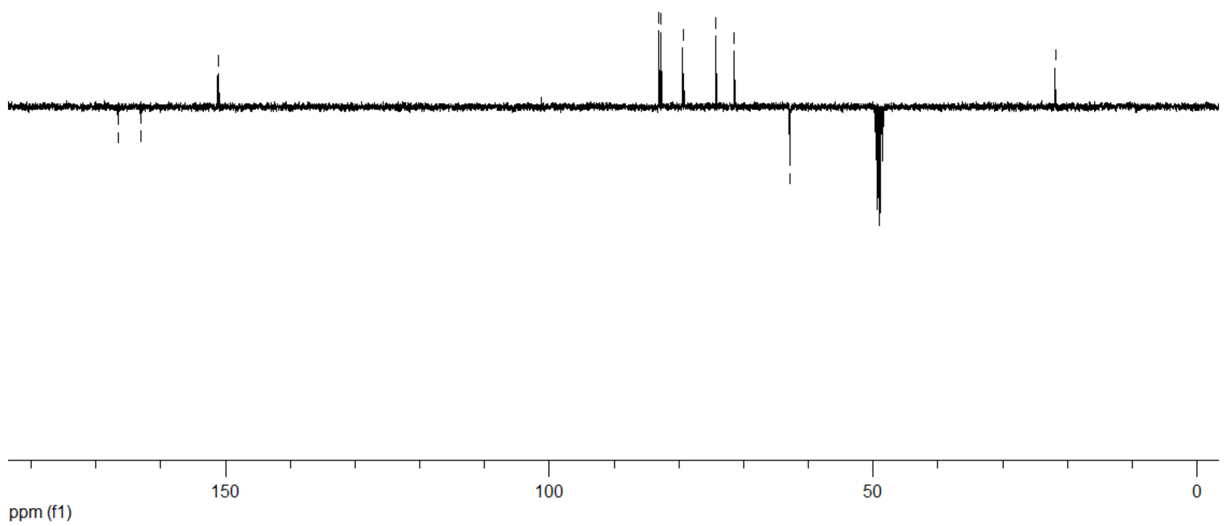

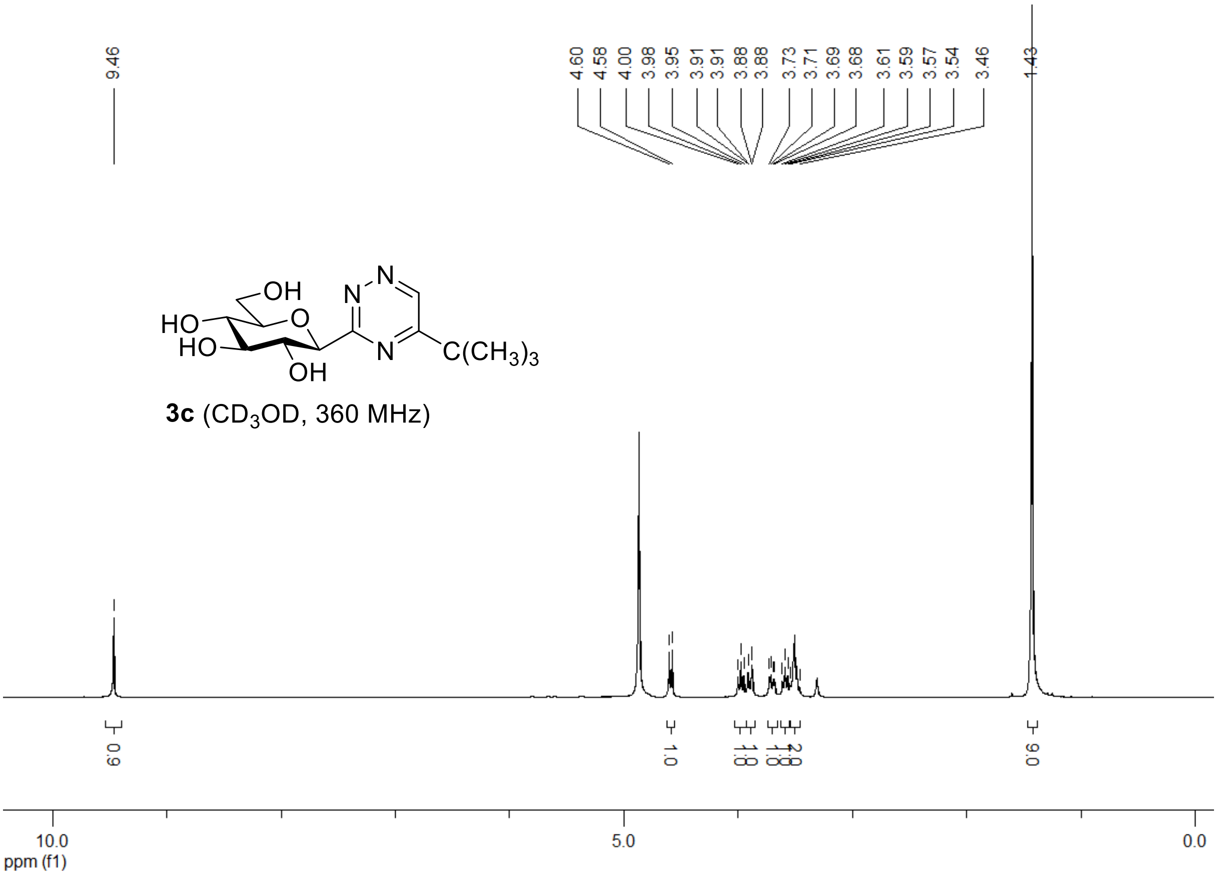
9.46
4.60 4.58 4.00 3.98 3.95 3.91 3.91 3.88 3.88 3.73 3.71 3.69 3.68 3.61 3.59 3.57 3.54 3.46
1.43
OH
HO
HO
OH
O
N
N
N
C(CH3)3
3c (CD3OD, 360 MHz)
0.9
1.0
1.0
1.0
1.0
2.8
9.0
10.0
5.0
0.0
ppm (f1)

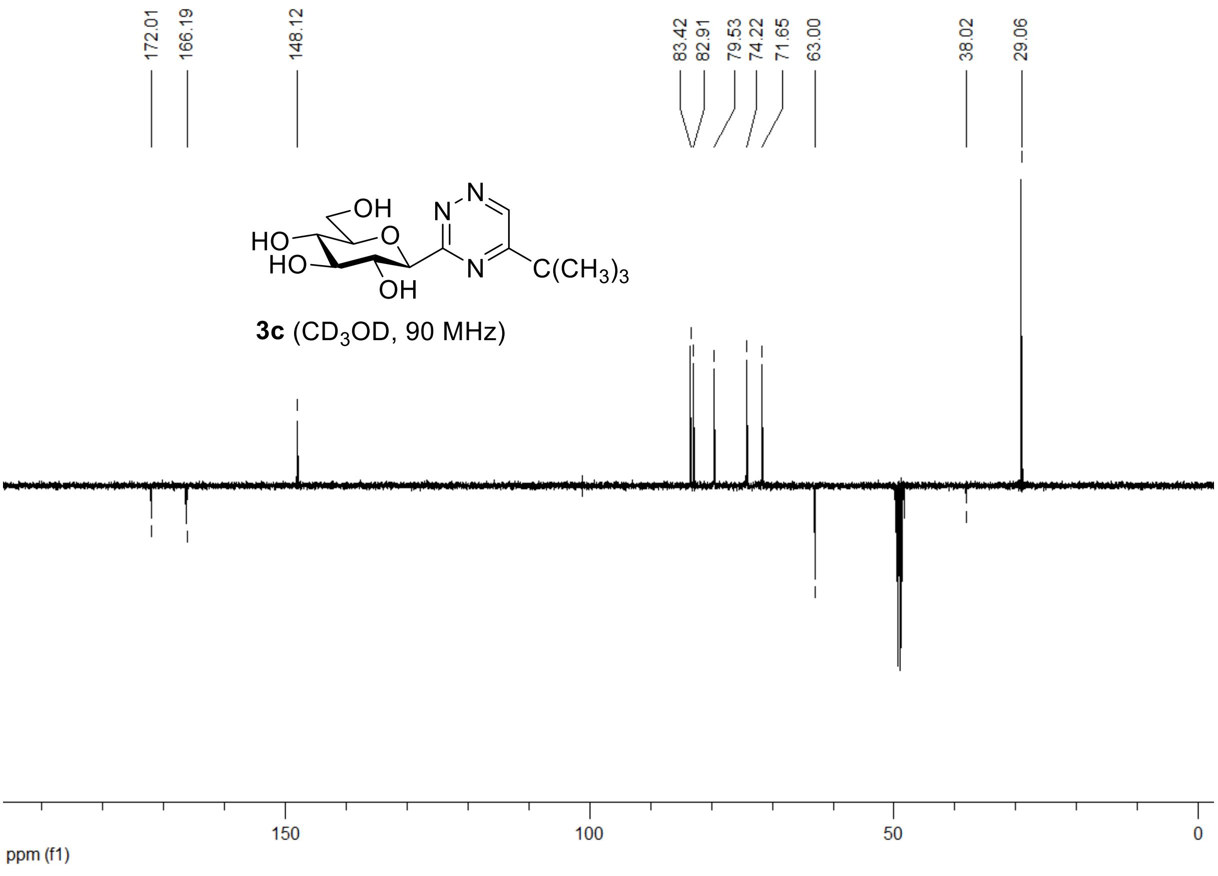
172.01 166.19 148.12
83.42 82.91 79.53 74.22 71.65 63.00
38.02 29.06
OH
HO
HO
OH
O
N
N
N
C(CH3)3
3c (CD3OD, 90 MHz)
150
100
50
0
ppm (f1)

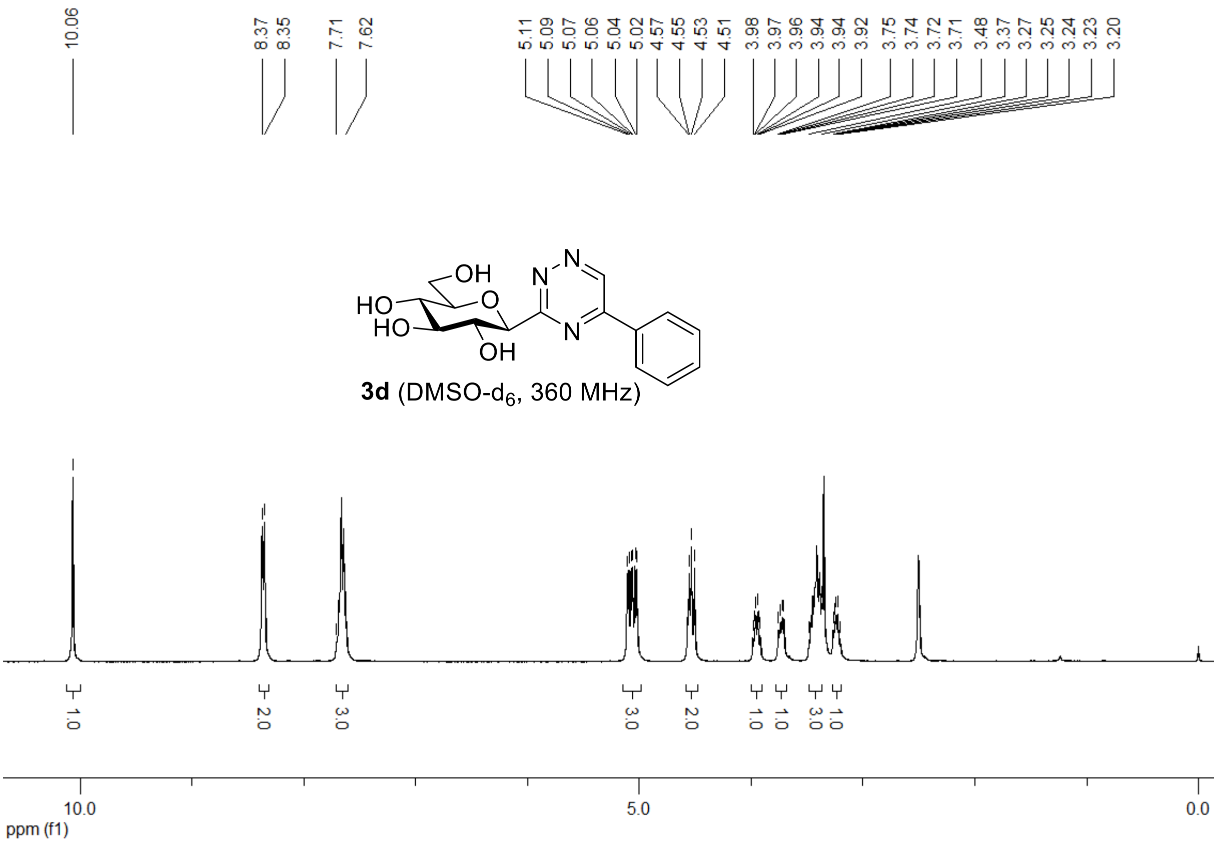


**3d** (DMSO-$d_6$, 360 MHz)

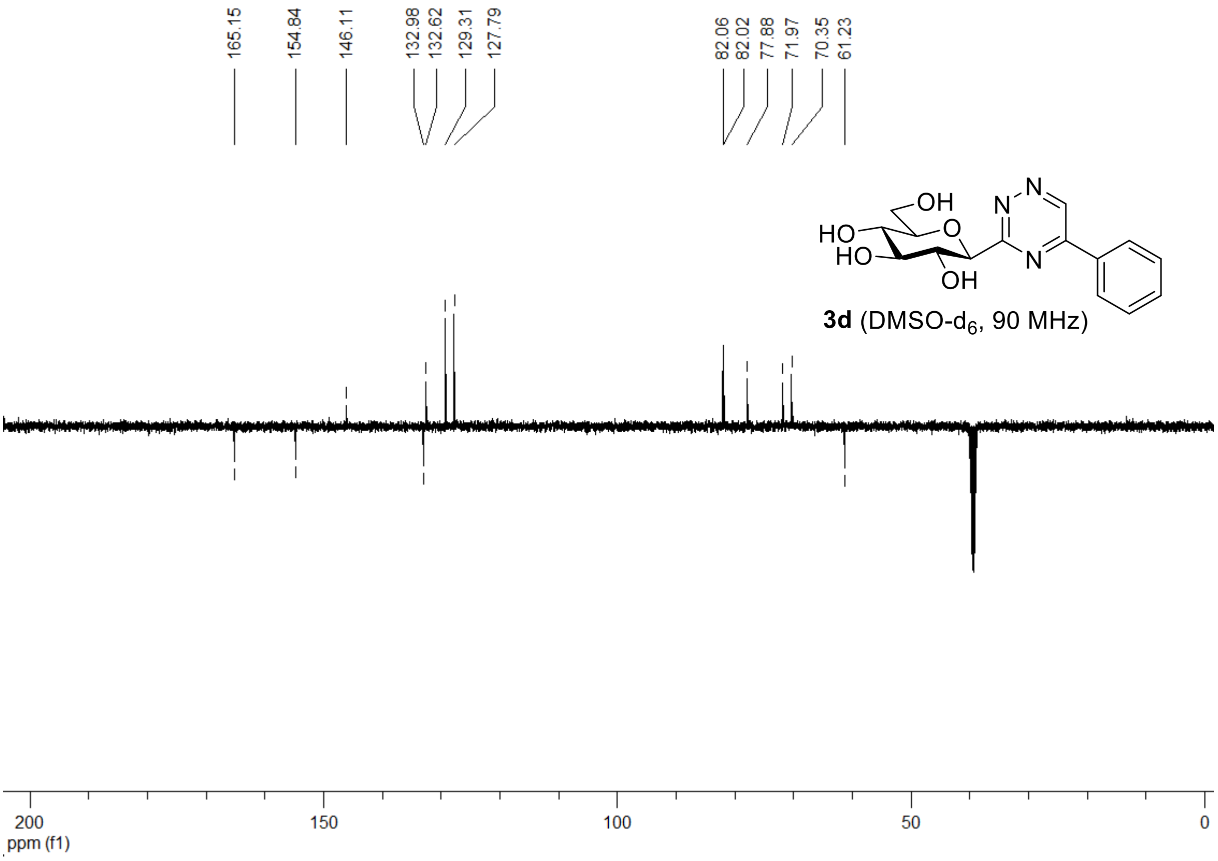


**3d** (DMSO-$d_6$, 90 MHz)

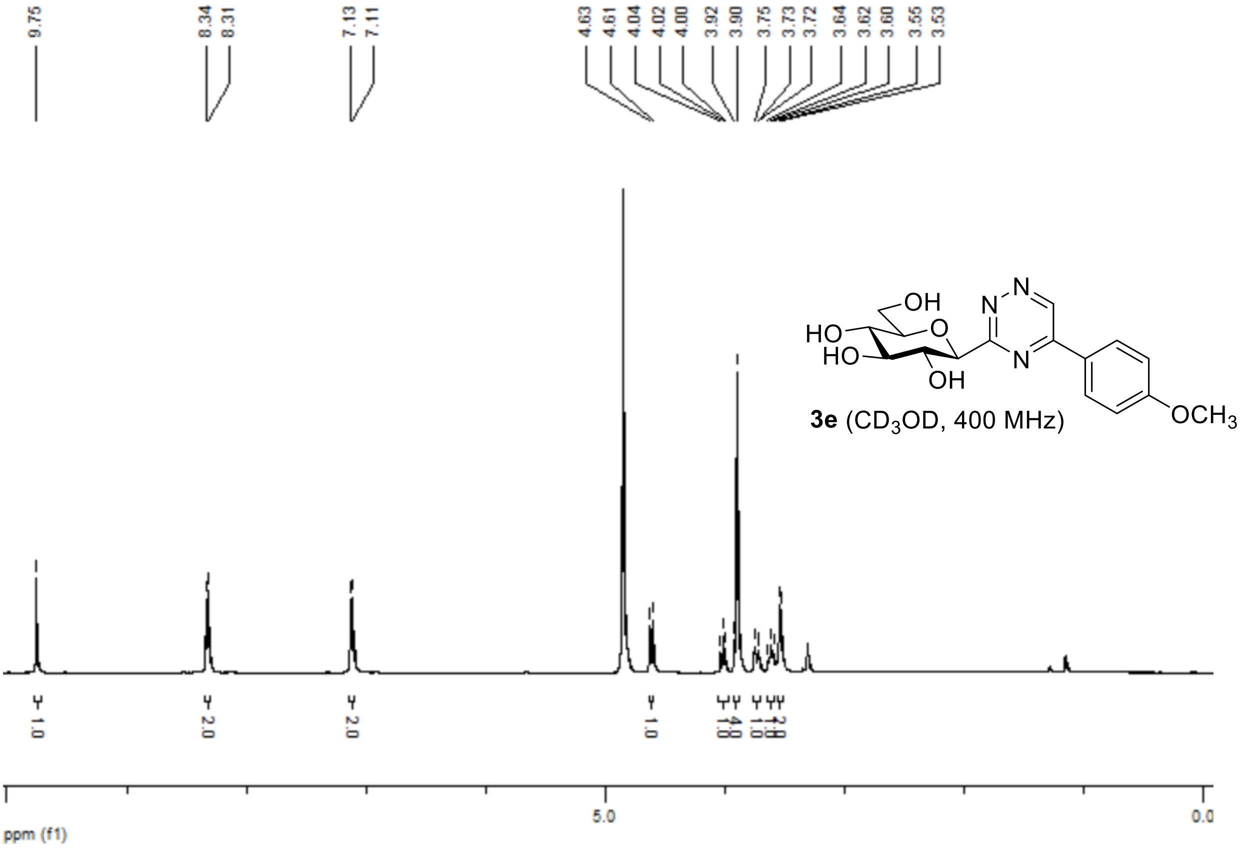
9.75
8.34
8.31
7.13
7.11
4.63
4.61
4.04
4.02
4.00
3.92
3.90
3.75
3.73
3.72
3.64
3.62
3.60
3.55
3.53
OH
HO
HO
O
OH
N
N
N
OCH3
3e (CD3OD, 400 MHz)
1.0
2.0
2.0
1.0
1.0
4.0
1.0
1.0
2.0
5.0
0.0
ppm (f1)

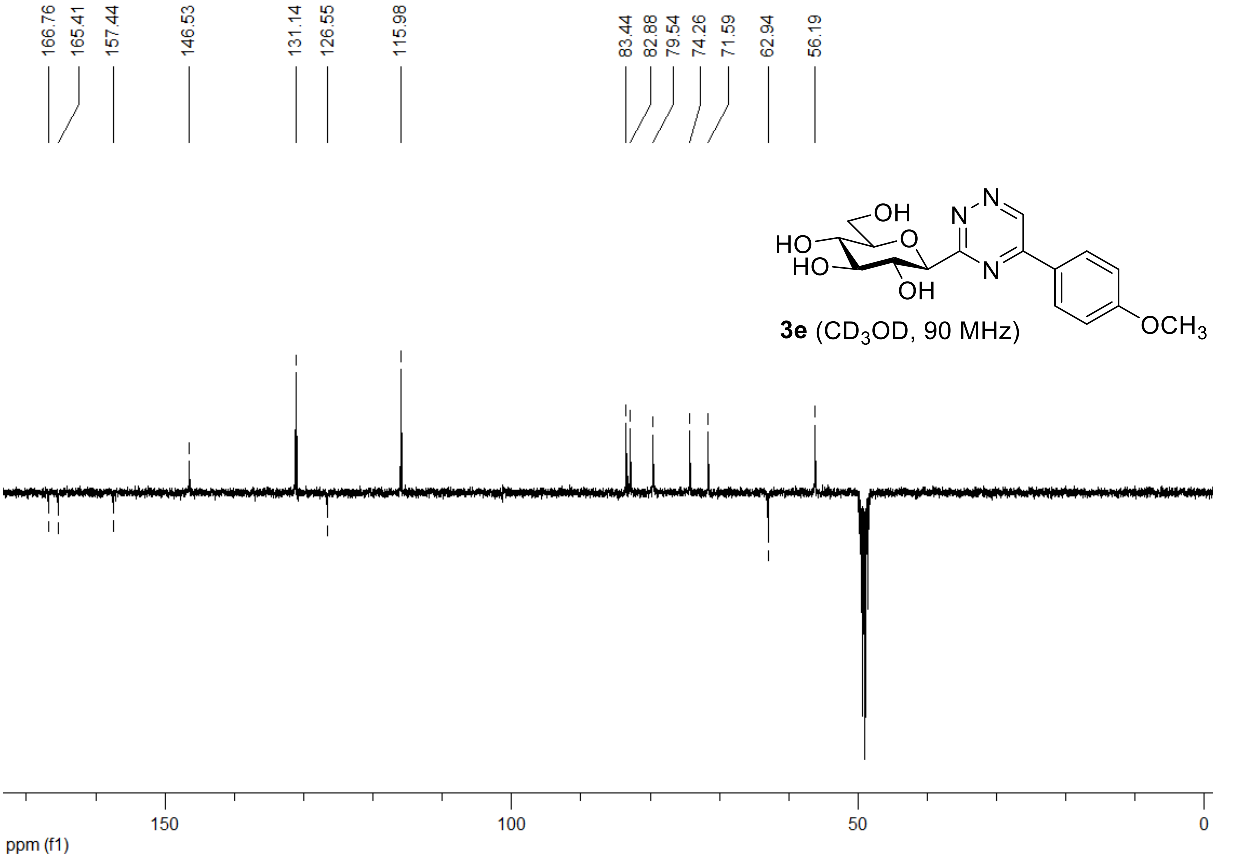
166.76
165.41
157.44
146.53
131.14
126.55
115.98
83.44
82.88
79.54
74.26
71.59
62.94
56.19
OH
HO
HO
O
OH
N
N
N
OCH3
3e (CD3OD, 90 MHz)
150
100
50
0
ppm (f1)

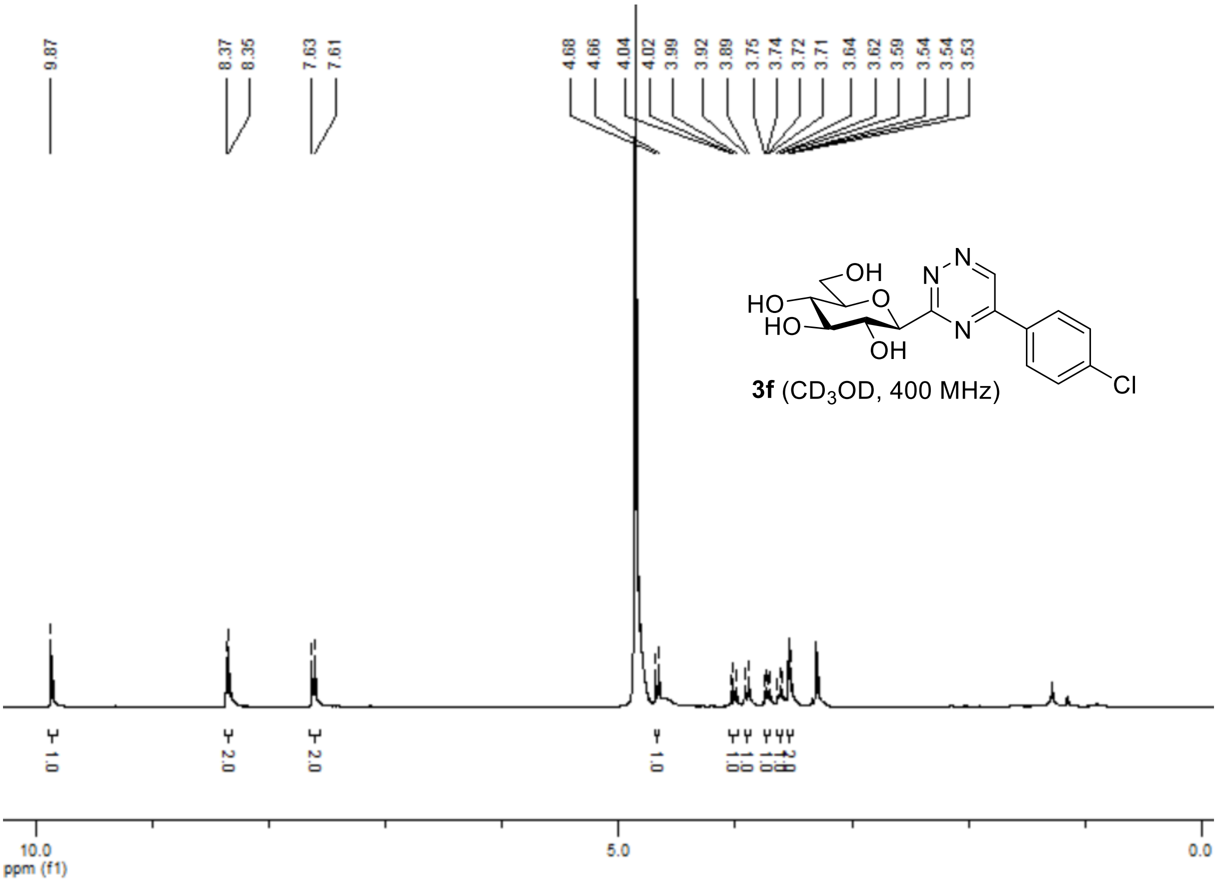
9.87
8.37
8.35
7.63
7.61
4.68
4.66
4.04
4.02
3.99
3.92
3.89
3.75
3.74
3.72
3.71
3.64
3.62
3.59
3.54
3.54
3.53
OH
N
N
HO
O
HO
N
OH
Cl
3f (CD3OD, 400 MHz)
1.0
2.0
2.0
1.0
10.0
5.0
0.0
ppm (f1)

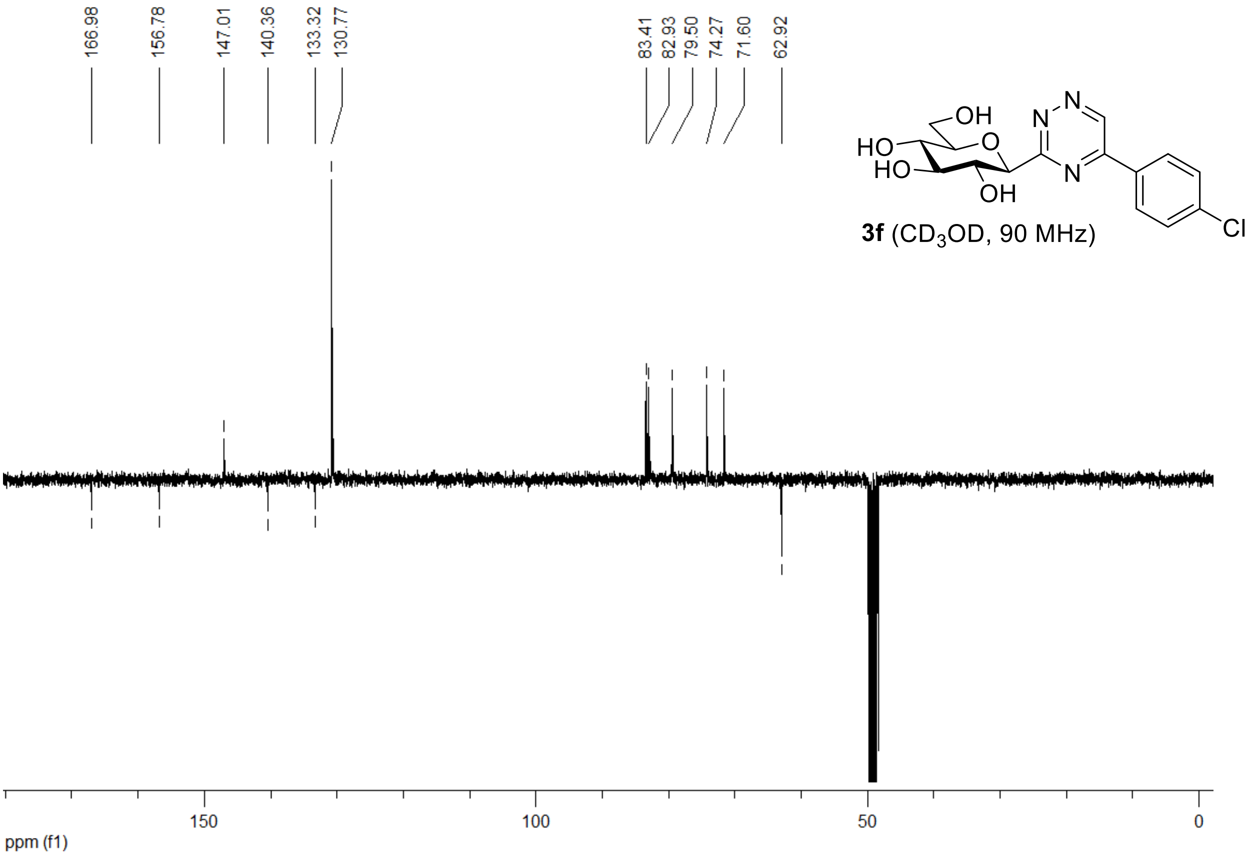
166.98
156.78
147.01
140.36
133.32
130.77
83.41
82.93
79.50
74.27
71.60
62.92
OH
N
N
HO
O
HO
N
OH
Cl
3f (CD3OD, 90 MHz)
150
100
50
0
ppm (f1)

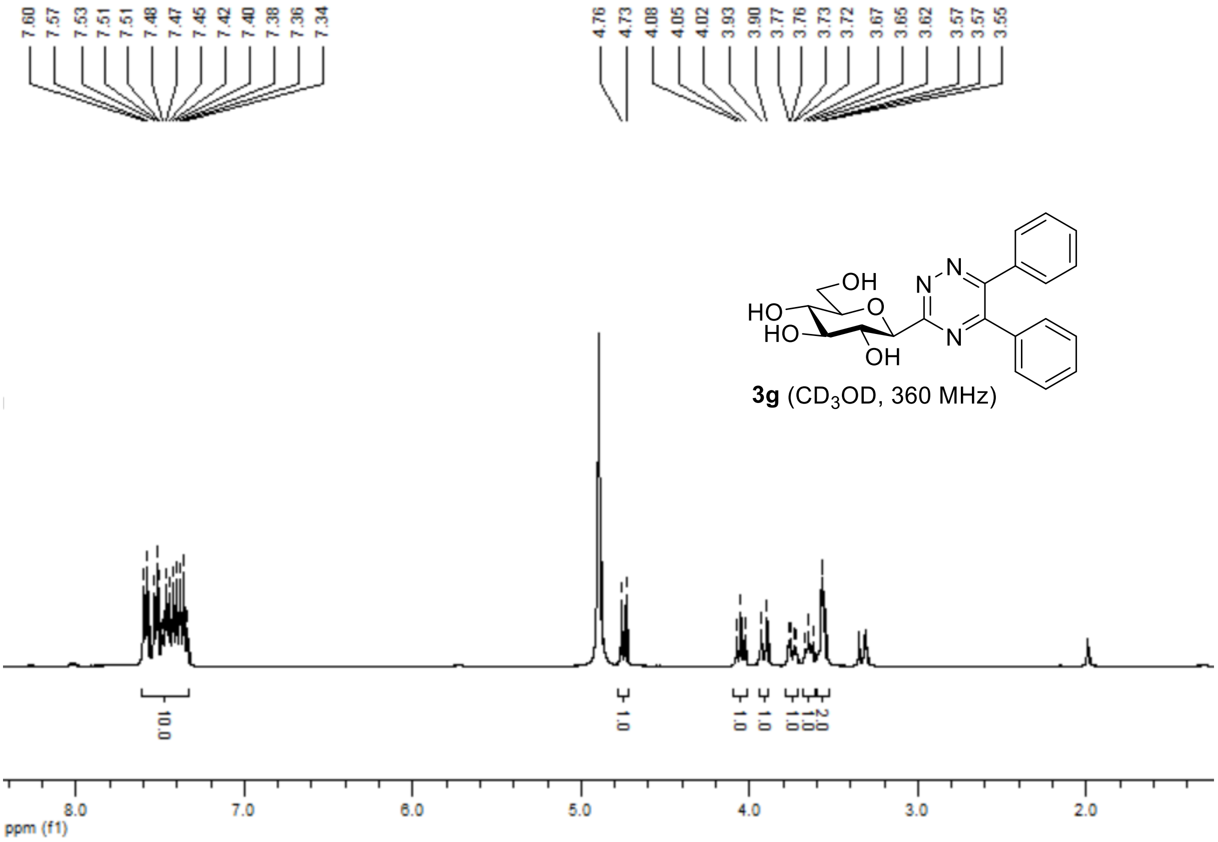
7.60 7.57 7.53 7.51 7.51 7.48 7.47 7.45 7.42 7.40 7.38 7.36 7.34
4.76 4.73 4.08 4.05 4.02 3.93 3.90 3.77 3.76 3.73 3.72 3.67 3.65 3.62 3.57 3.57 3.55
OH
HO
HO
O
OH
N
N
N
3g (CD$_3$OD, 360 MHz)
10.0
1.0
1.0 1.0 1.0 1.0 2.0
8.0 7.0 6.0 5.0 4.0 3.0 2.0
ppm (f1)

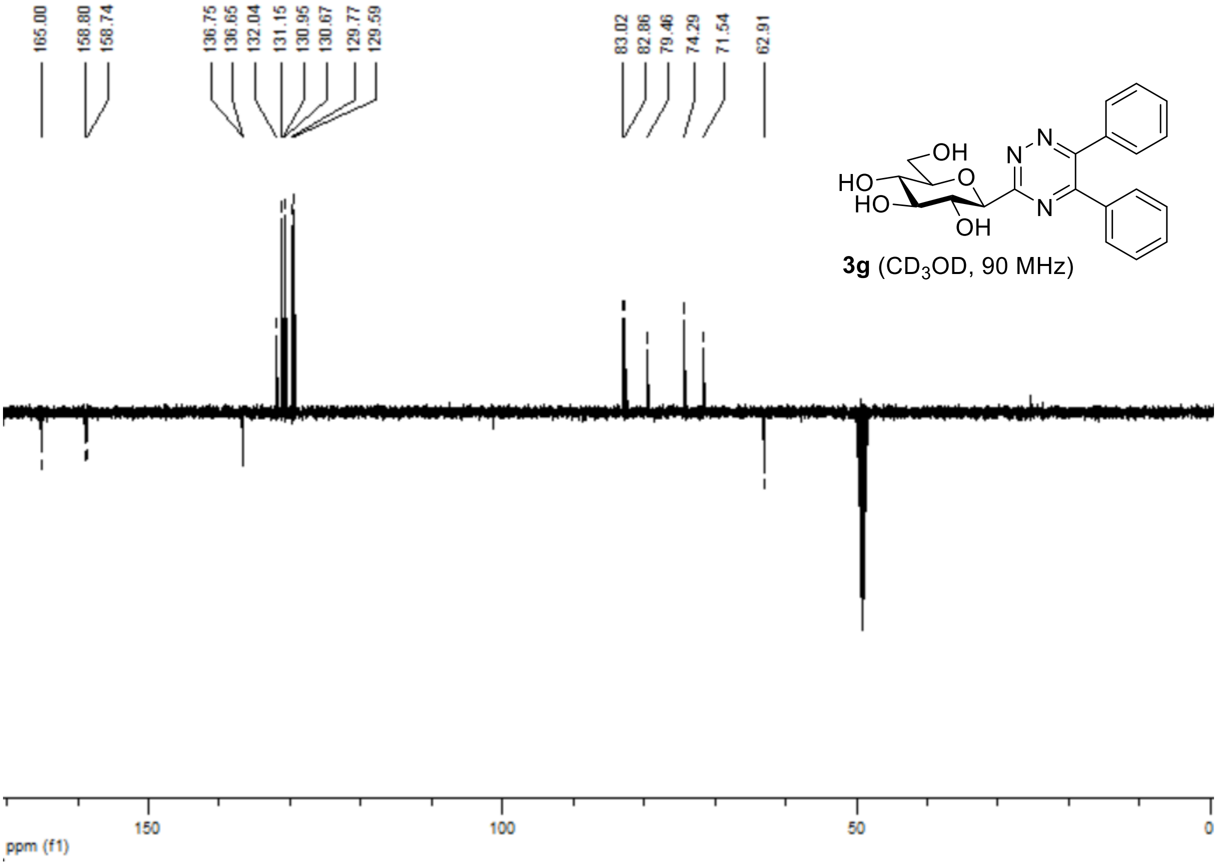
165.00 158.80 158.74
136.75 136.65 132.04 131.15 130.95 130.67 129.77 129.59
83.02 82.86 79.46 74.29 71.54 62.91
OH
HO
HO
O
OH
N
N
N
3g (CD$_3$OD, 90 MHz)
150 100 50 0
ppm (f1)

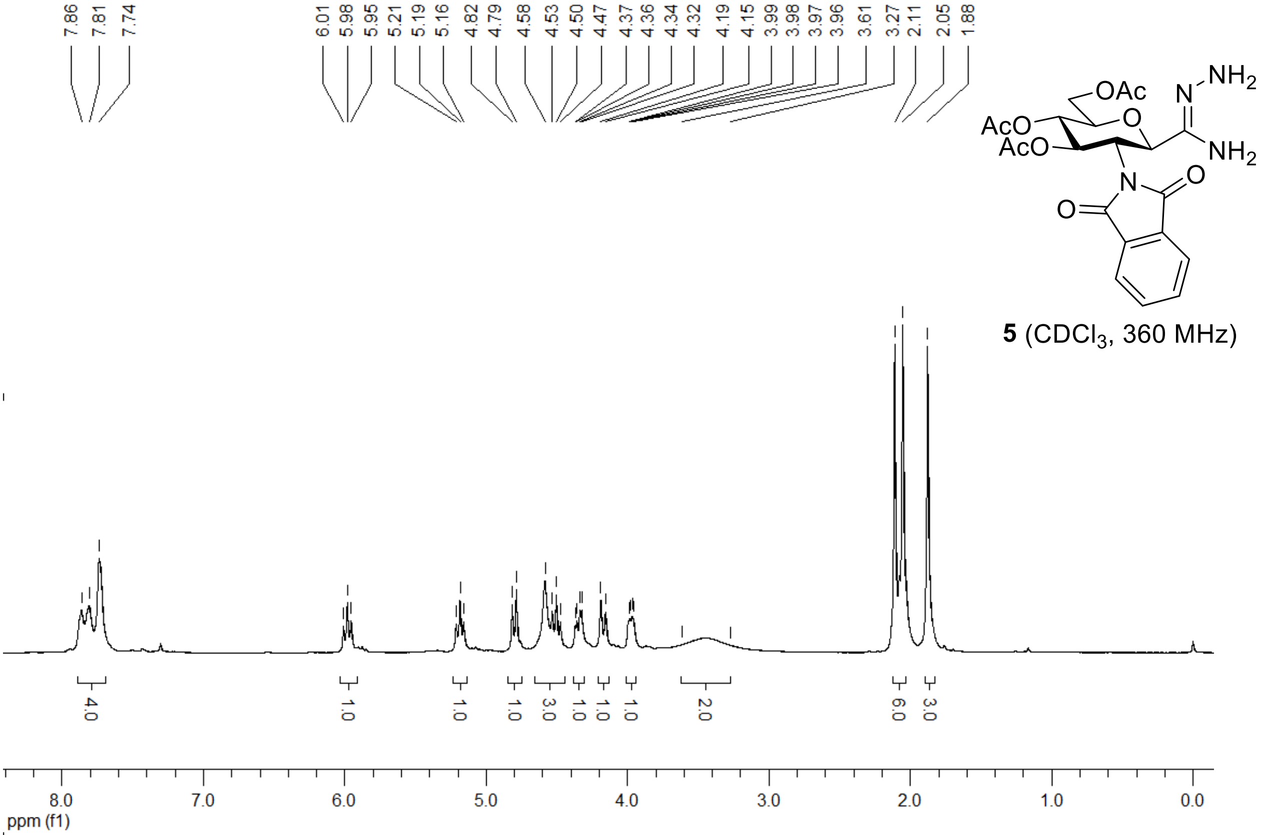


**5** ($CDCl_3$, 360 MHz)

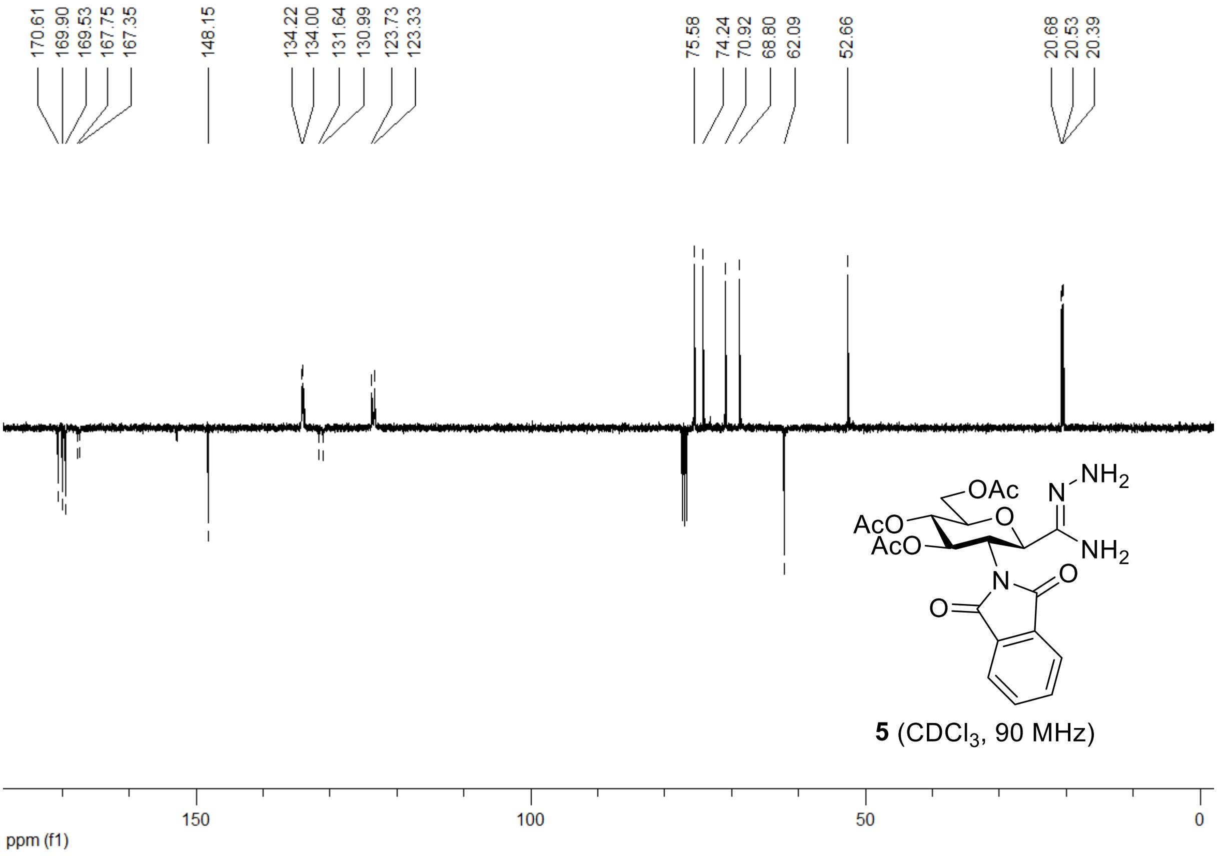


**5** ($CDCl_3$, 90 MHz)

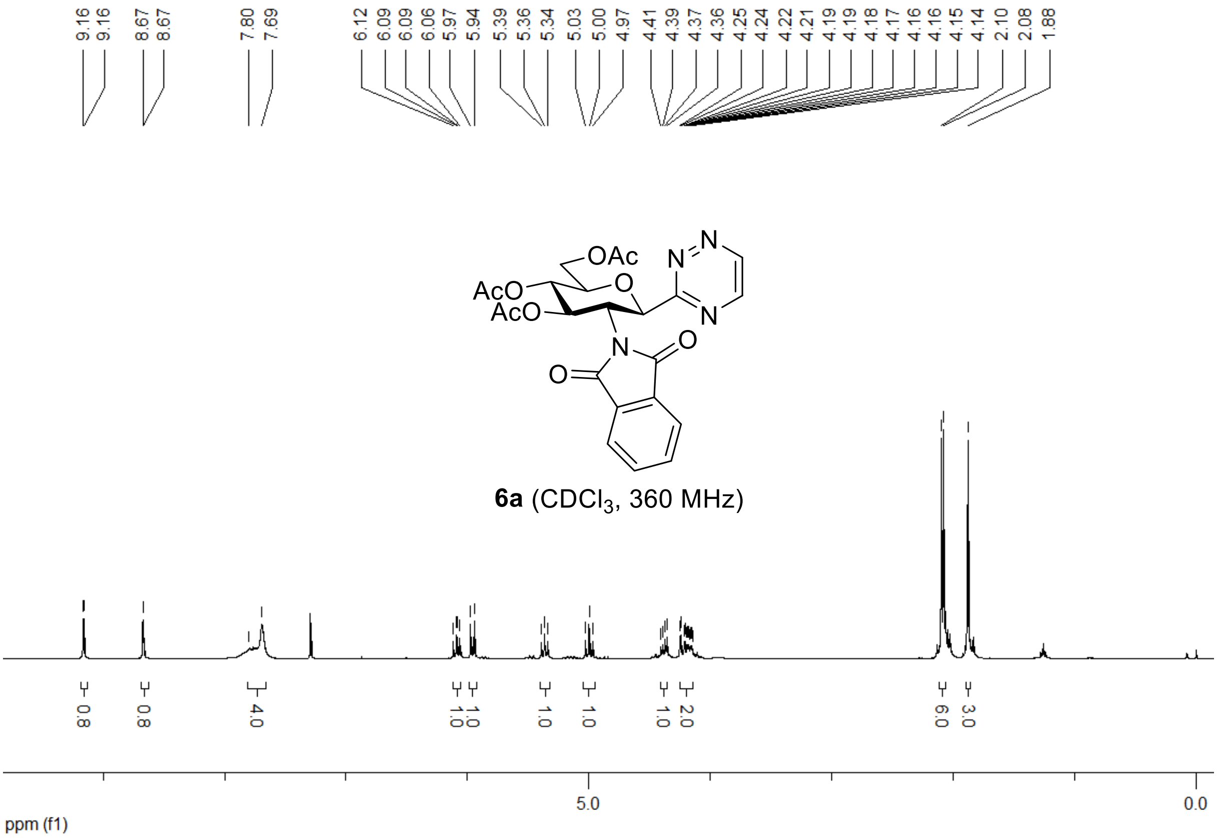


**6a** ($CDCl_3$, 360 MHz)

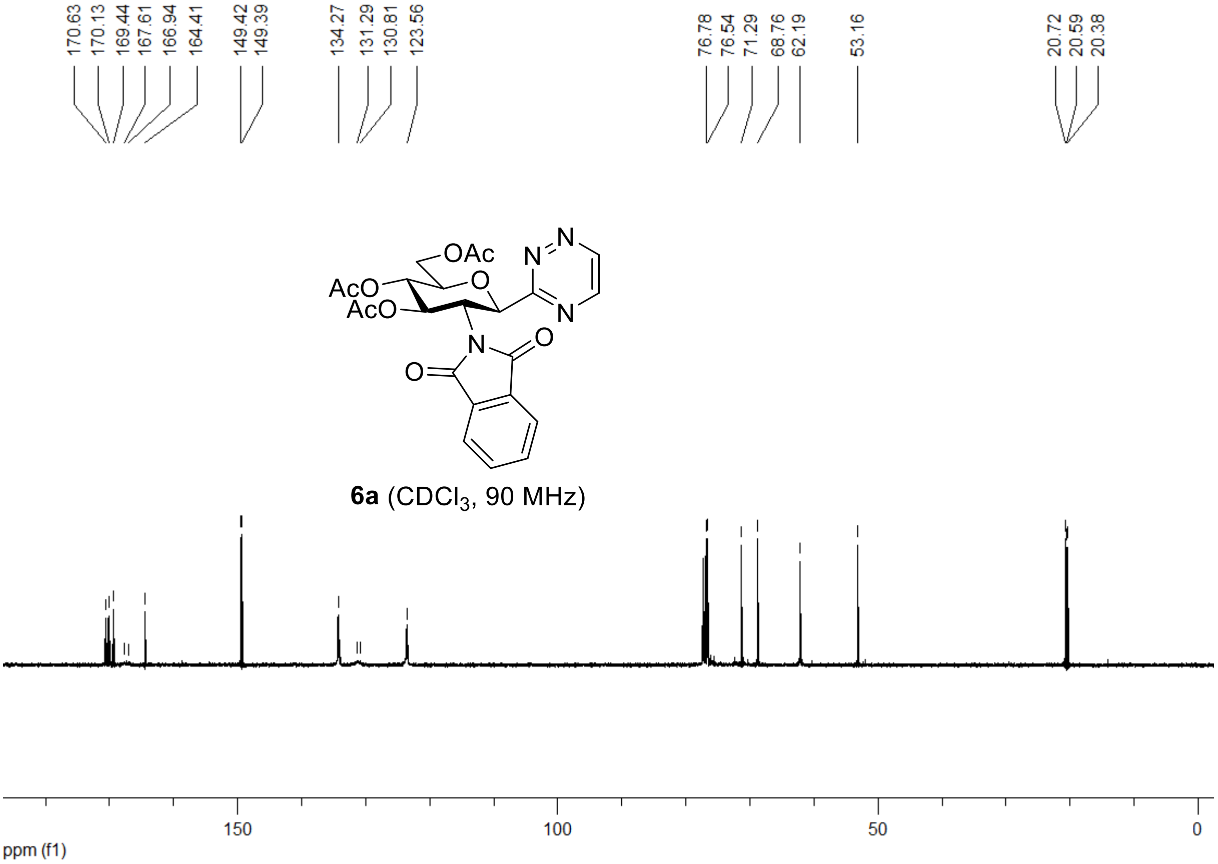


**6a** ($CDCl_3$, 90 MHz)

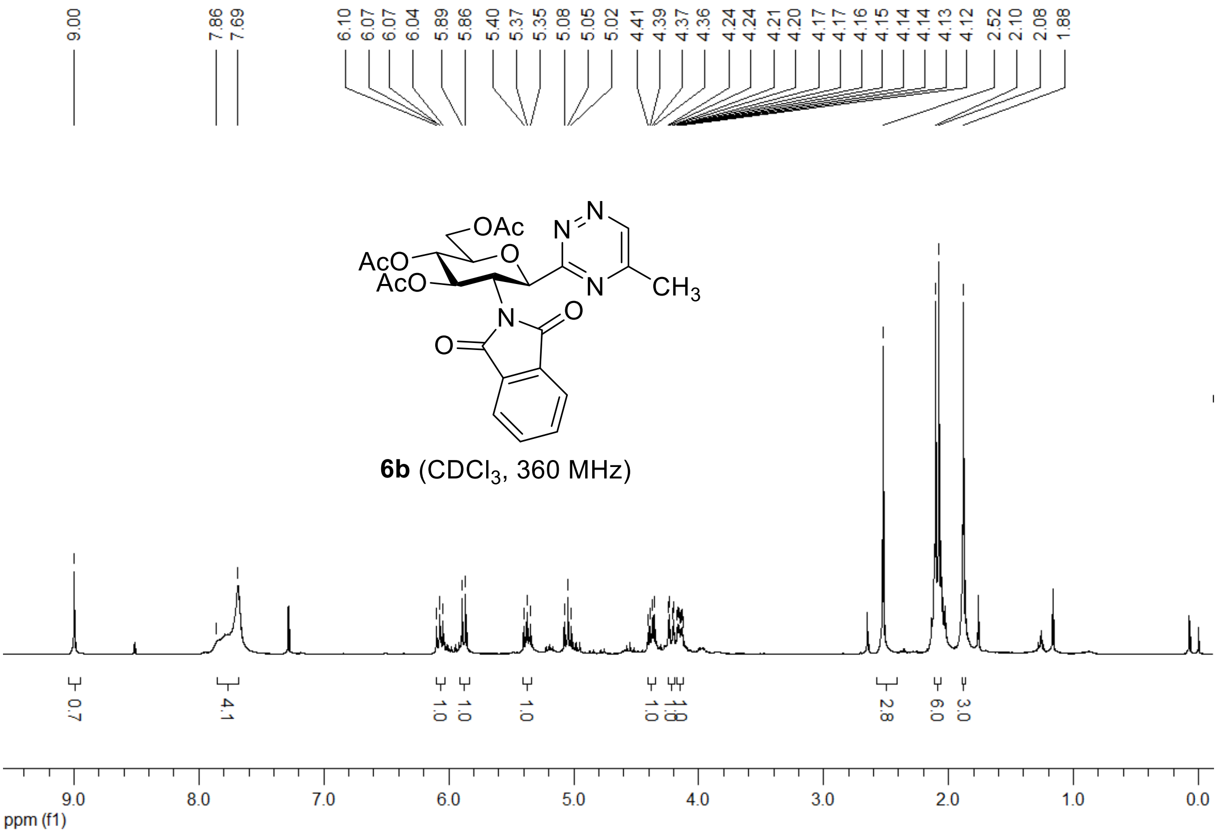


**6b** ($CDCl_3$, 360 MHz)

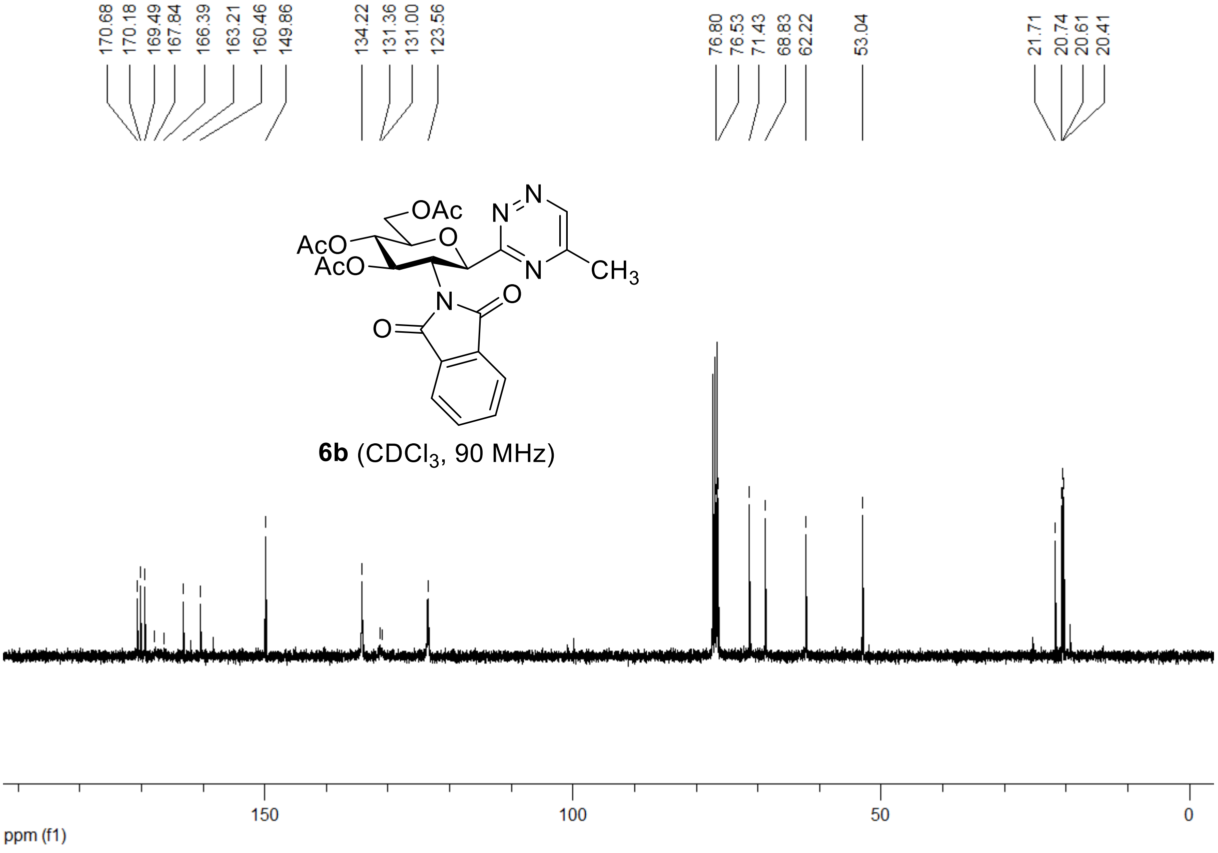


**6b** ($CDCl_3$, 90 MHz)

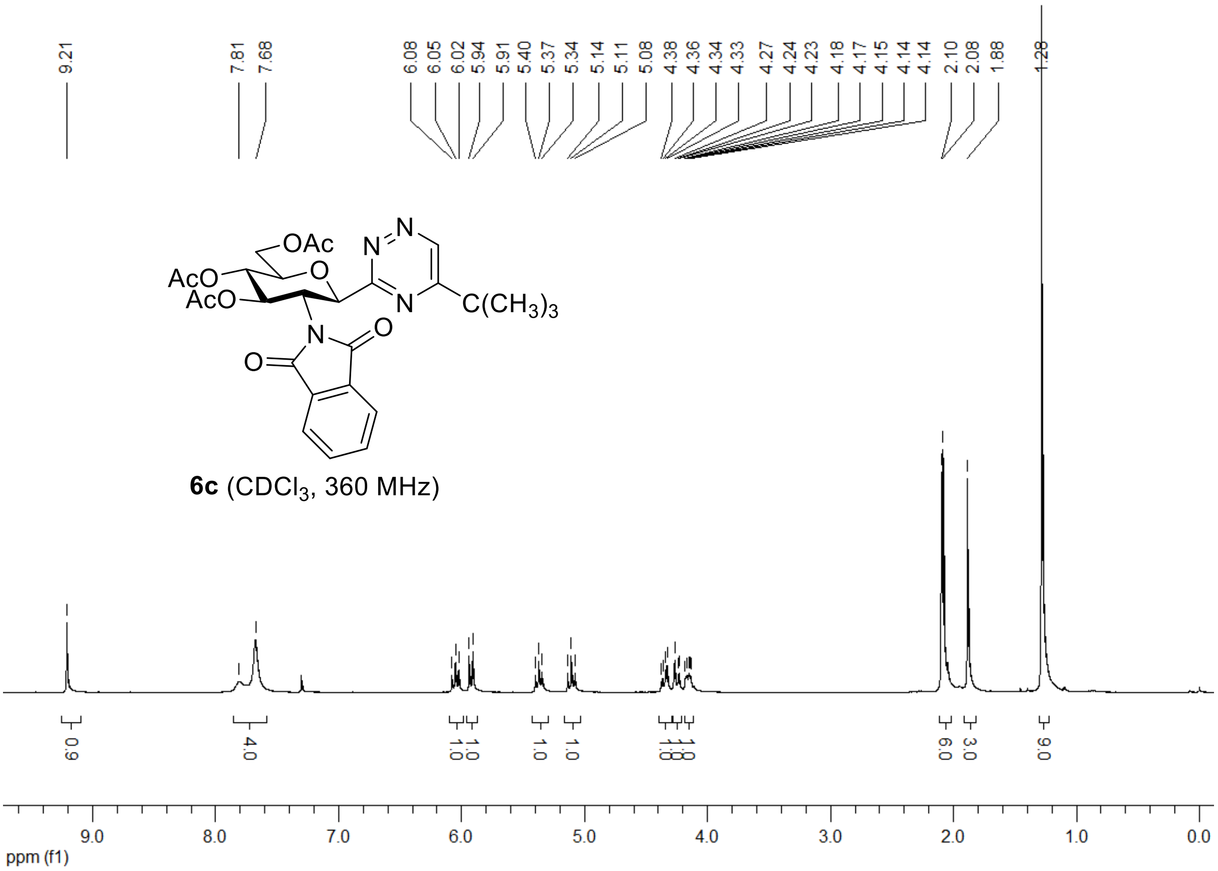
9.21
7.81
7.68
6.08
6.05
6.02
5.94
5.91
5.40
5.37
5.34
5.14
5.11
5.08
4.38
4.36
4.34
4.33
4.27
4.24
4.23
4.18
4.17
4.15
4.14
4.14
2.10
2.08
1.88
1.28
OAc
AcO
AcO
O
N
N
N
N
C(CH3)3
N
O
O
6c (CDCl3, 360 MHz)
0.9
4.0
1.0
1.0
1.0
1.0
1.0
1.0
6.0
3.0
9.0
9.0
8.0
7.0
6.0
5.0
4.0
3.0
2.0
1.0
0.0
ppm (f1)

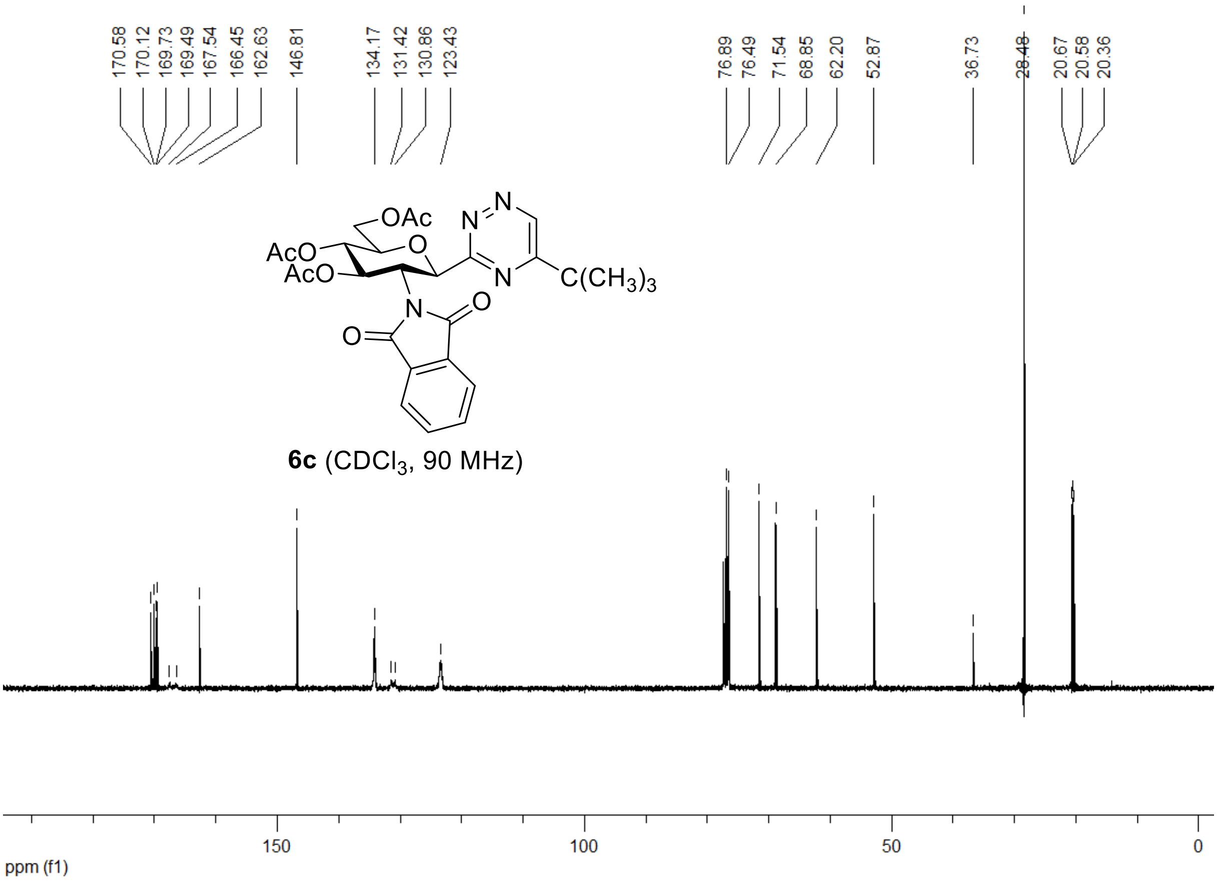
170.58
170.12
169.73
169.49
167.54
166.45
162.63
146.81
134.17
131.42
130.86
123.43
76.89
76.49
71.54
68.85
62.20
52.87
36.73
28.48
20.67
20.58
20.36
OAc
AcO
AcO
O
N
N
N
N
C(CH3)3
O
O
6c (CDCl3, 90 MHz)
150
100
50
0
ppm (f1)

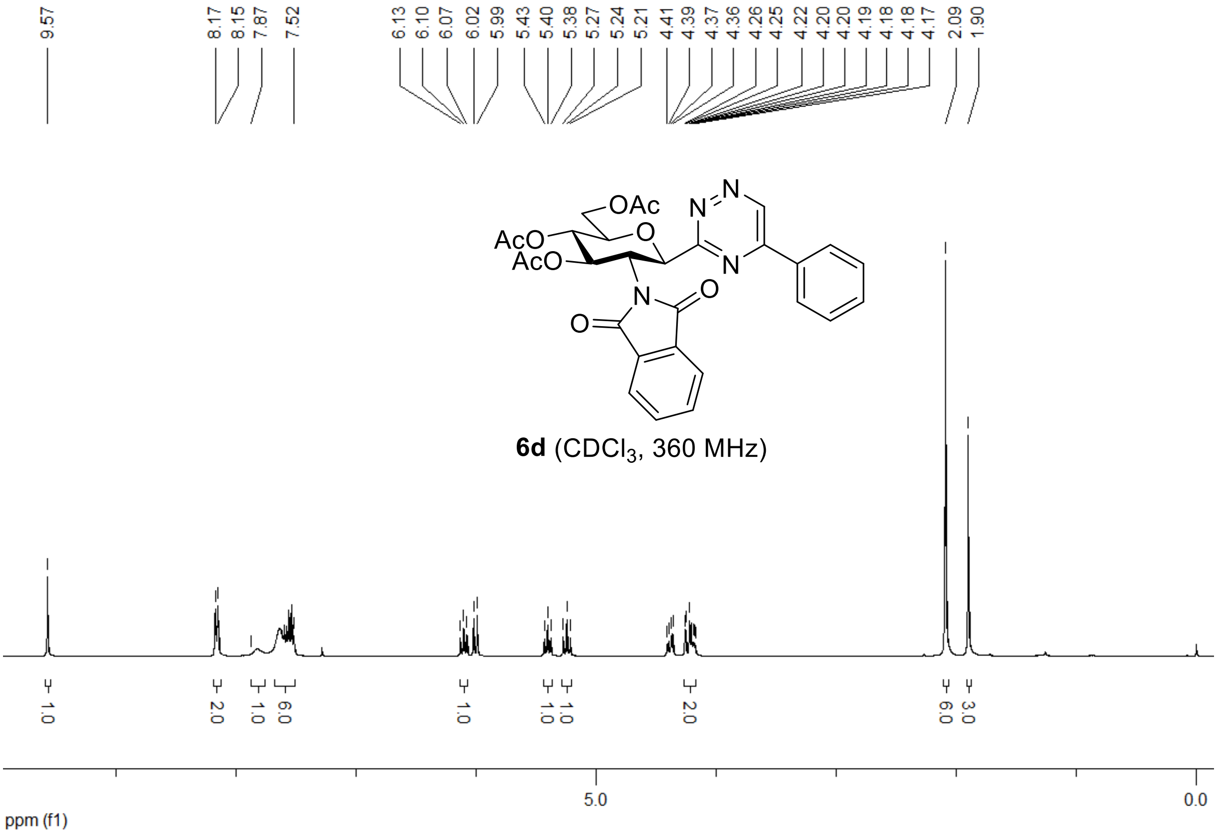
9.57
8.17
8.15
7.87
7.52
6.13
6.10
6.07
6.02
5.99
5.43
5.40
5.38
5.27
5.24
5.21
4.41
4.39
4.37
4.36
4.26
4.25
4.22
4.20
4.20
4.19
4.18
4.18
4.17
2.09
1.90
OAc
AcO
AcO
O
N
N
N
N
O
O
**6d** ($CDCl_3$, 360 MHz)
1.0
2.0
1.0
6.0
1.0
1.0
1.0
2.0
6.0
3.0
5.0
0.0
ppm (f1)

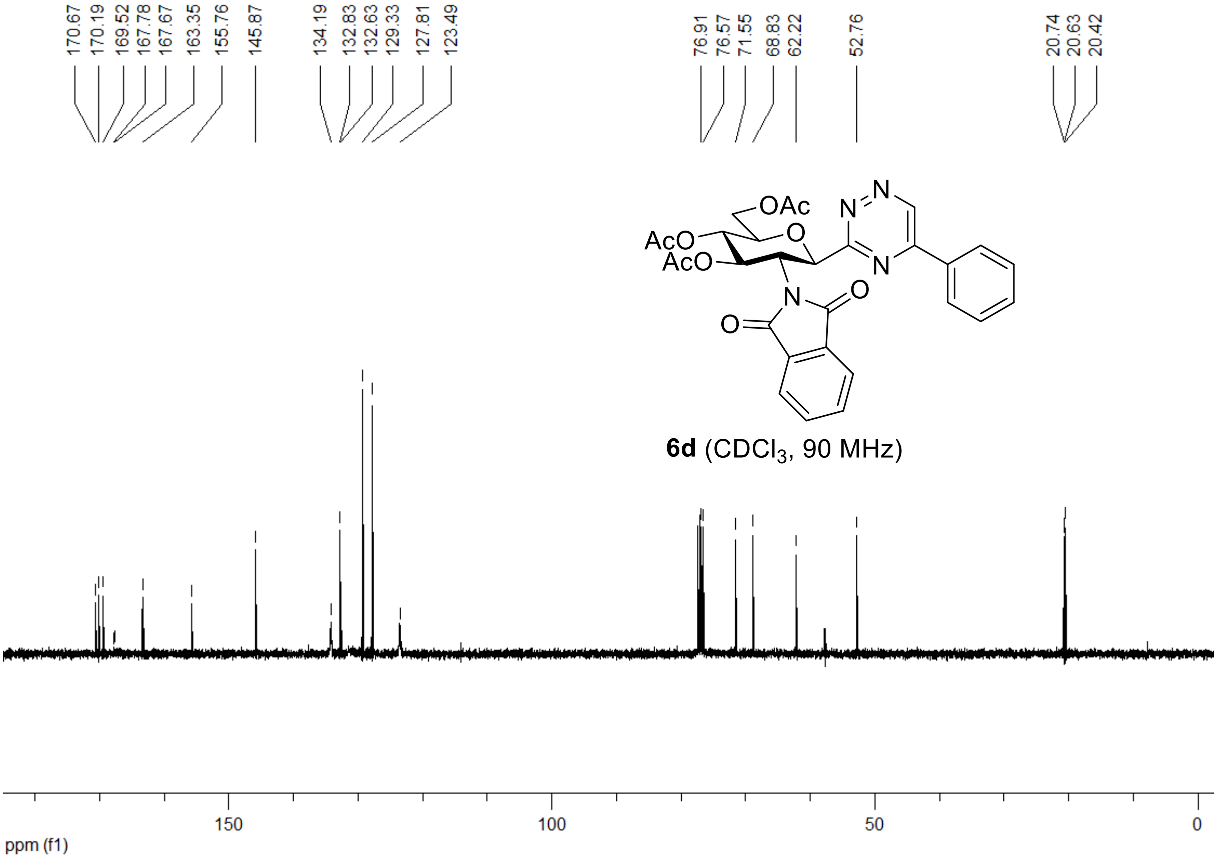
170.67
170.19
169.52
167.78
167.67
163.35
155.76
145.87
134.19
132.83
132.63
129.33
127.81
123.49
76.91
76.57
71.55
68.83
62.22
52.76
20.74
20.63
20.42
OAc
AcO
AcO
O
N
N
N
N
O
O
**6d** ($CDCl_3$, 90 MHz)
150
100
50
0
ppm (f1)

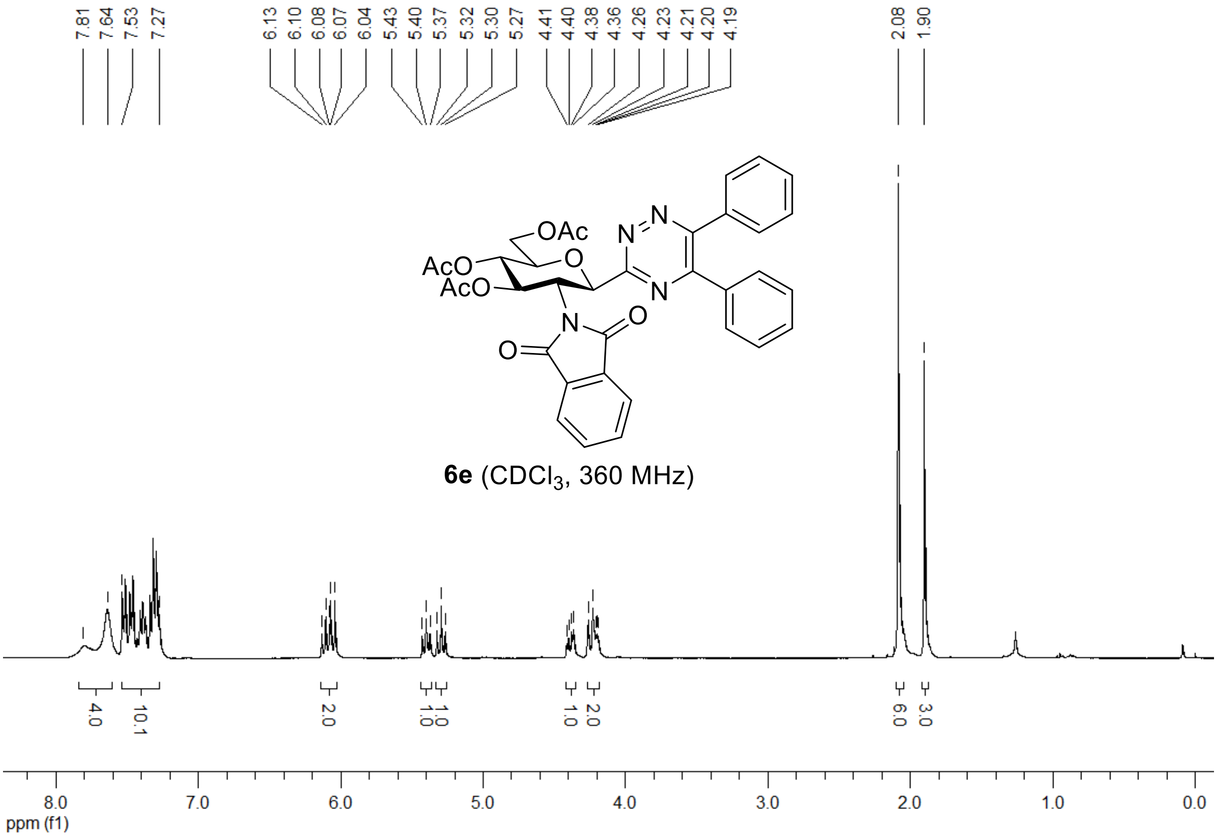


**6e** ($CDCl_3$, 360 MHz)

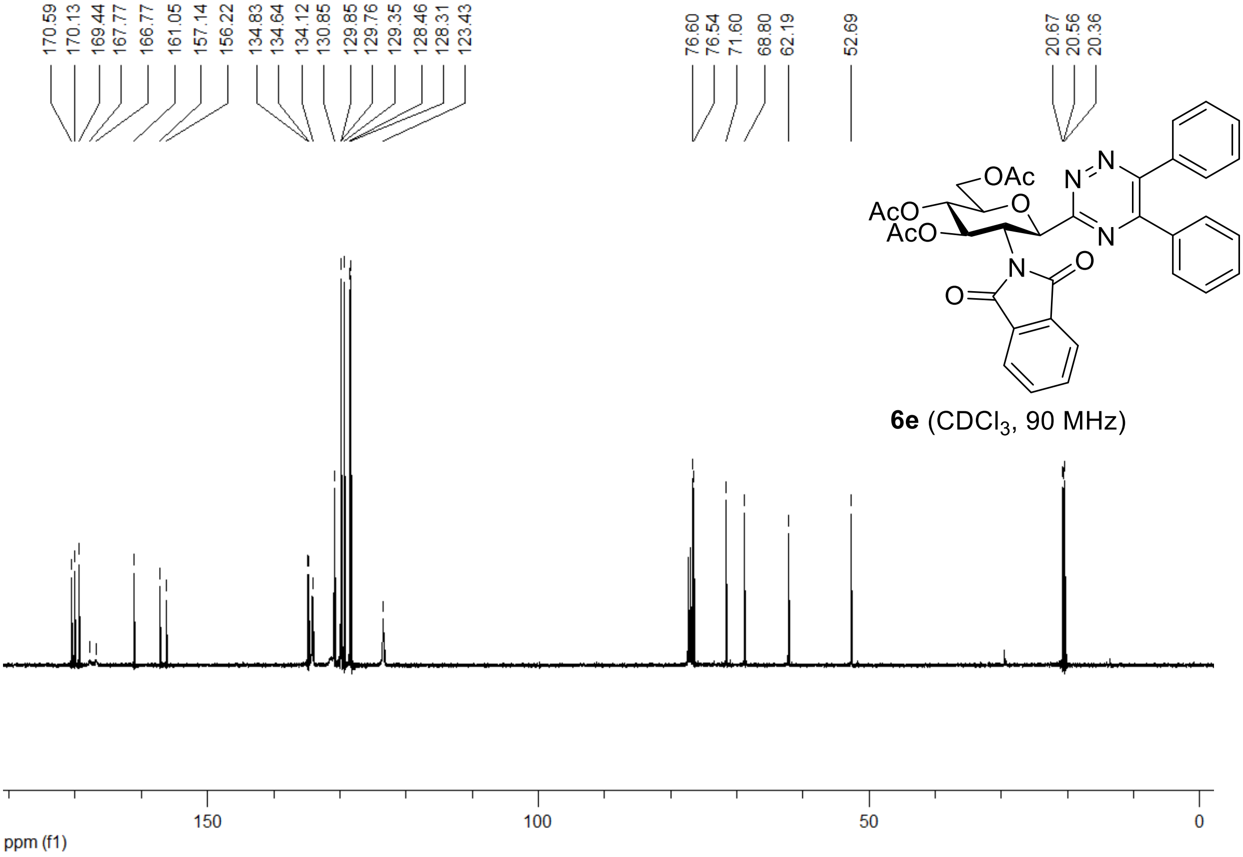


**6e** ($CDCl_3$, 90 MHz)

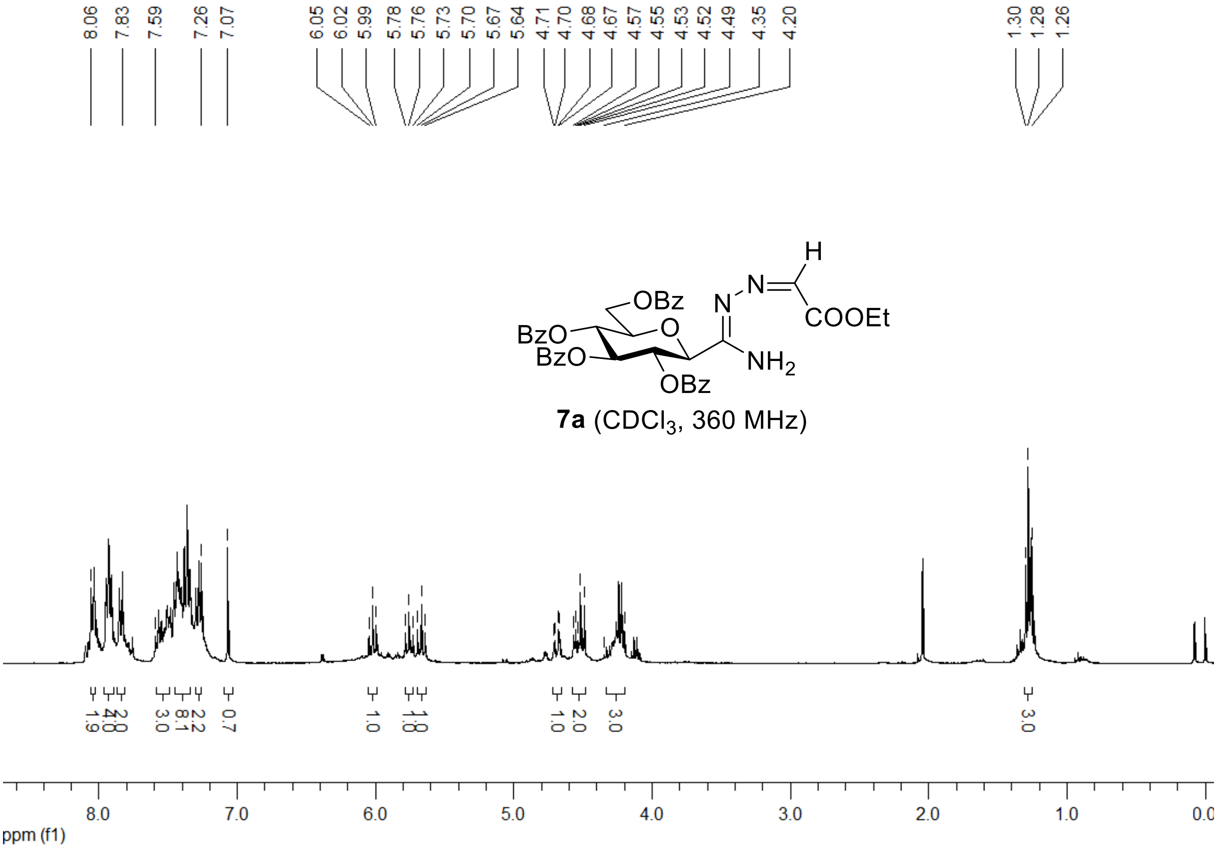
7a (CDCl3, 360 MHz)
ppm (f1)

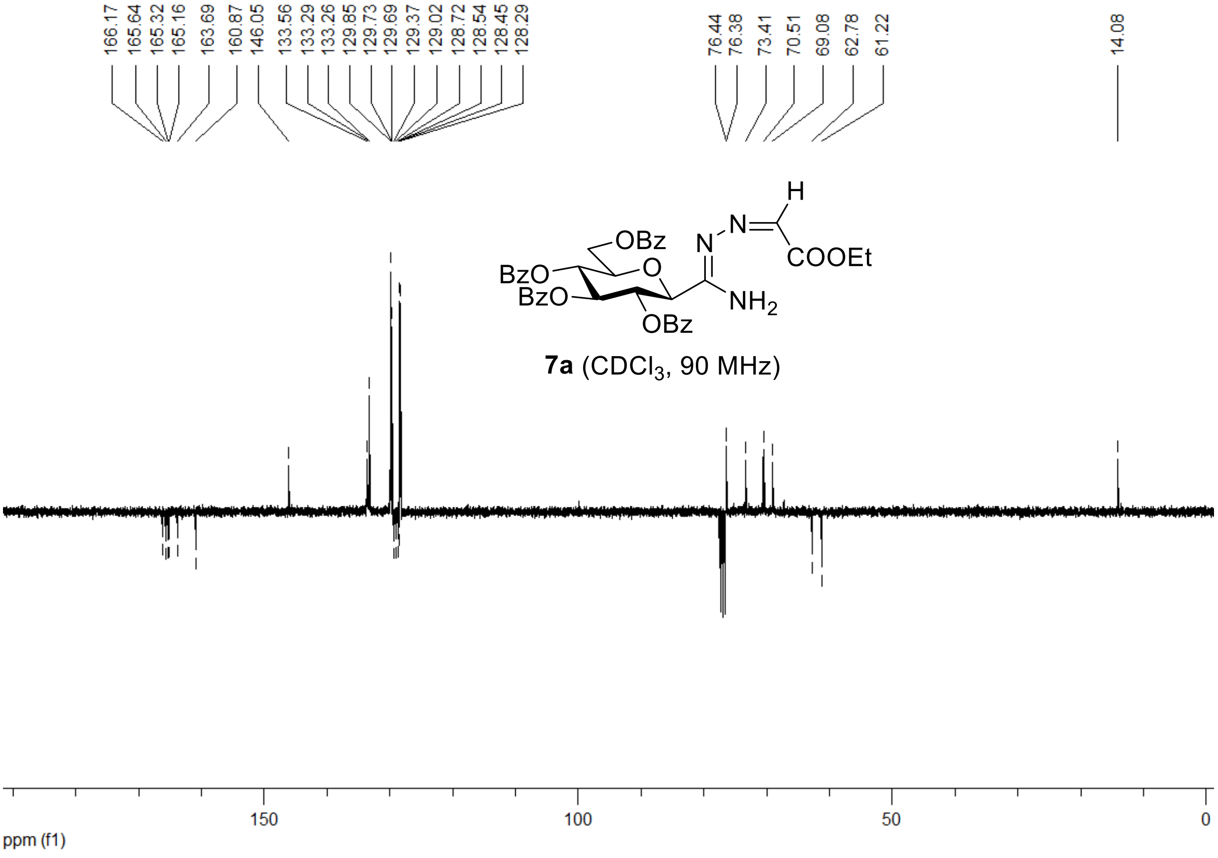
7a (CDCl3, 90 MHz)
ppm (f1)

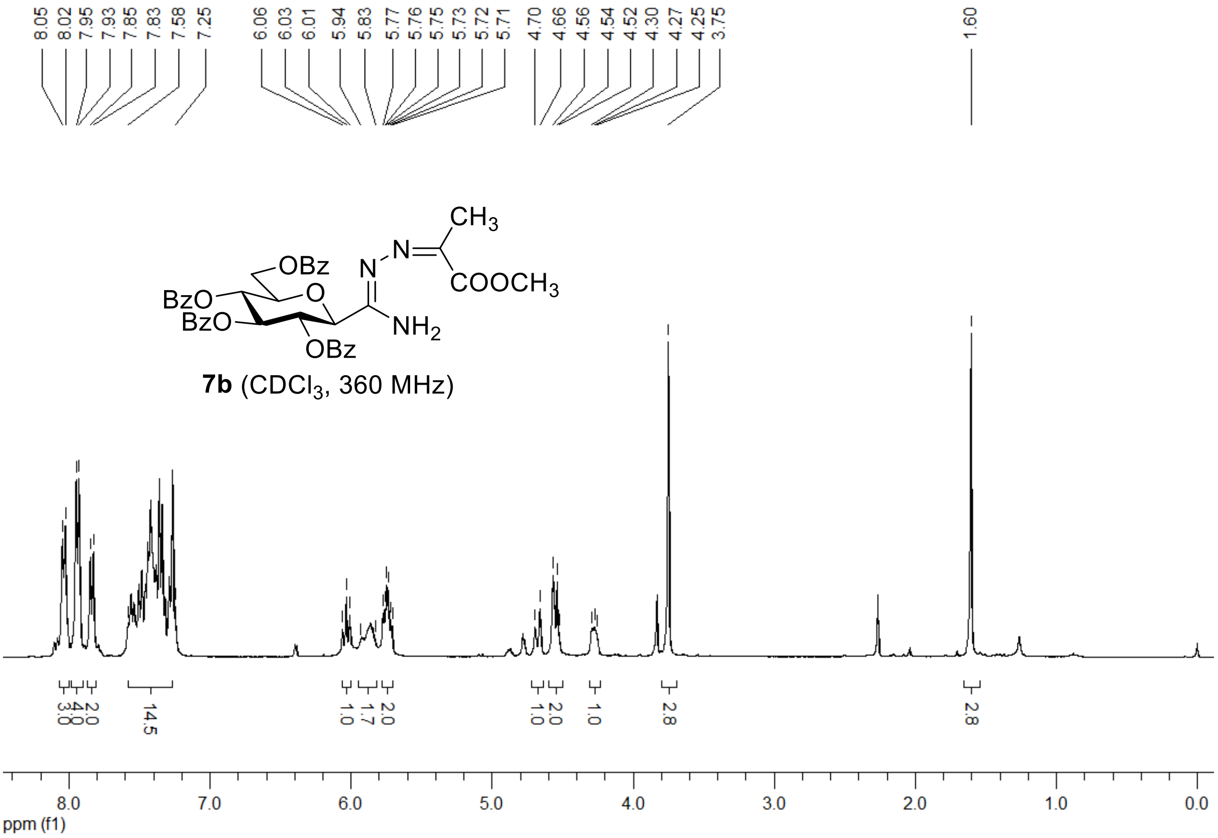
8.05 8.02 7.95 7.93 7.85 7.83 7.58 7.25 6.06 6.03 6.01 5.94 5.83 5.77 5.76 5.75 5.73 5.72 5.71 4.70 4.66 4.56 4.54 4.52 4.30 4.27 4.25 3.75 1.60
CH3
N
N
OBz
BzO
O
BzO
COOCH3
NH2
OBz
7b (CDCl3, 360 MHz)
3.0 4.0 2.0 14.5 1.0 1.7 2.0 1.0 2.0 1.0 2.8 2.8
8.0 7.0 6.0 5.0 4.0 3.0 2.0 1.0 0.0
ppm (f1)

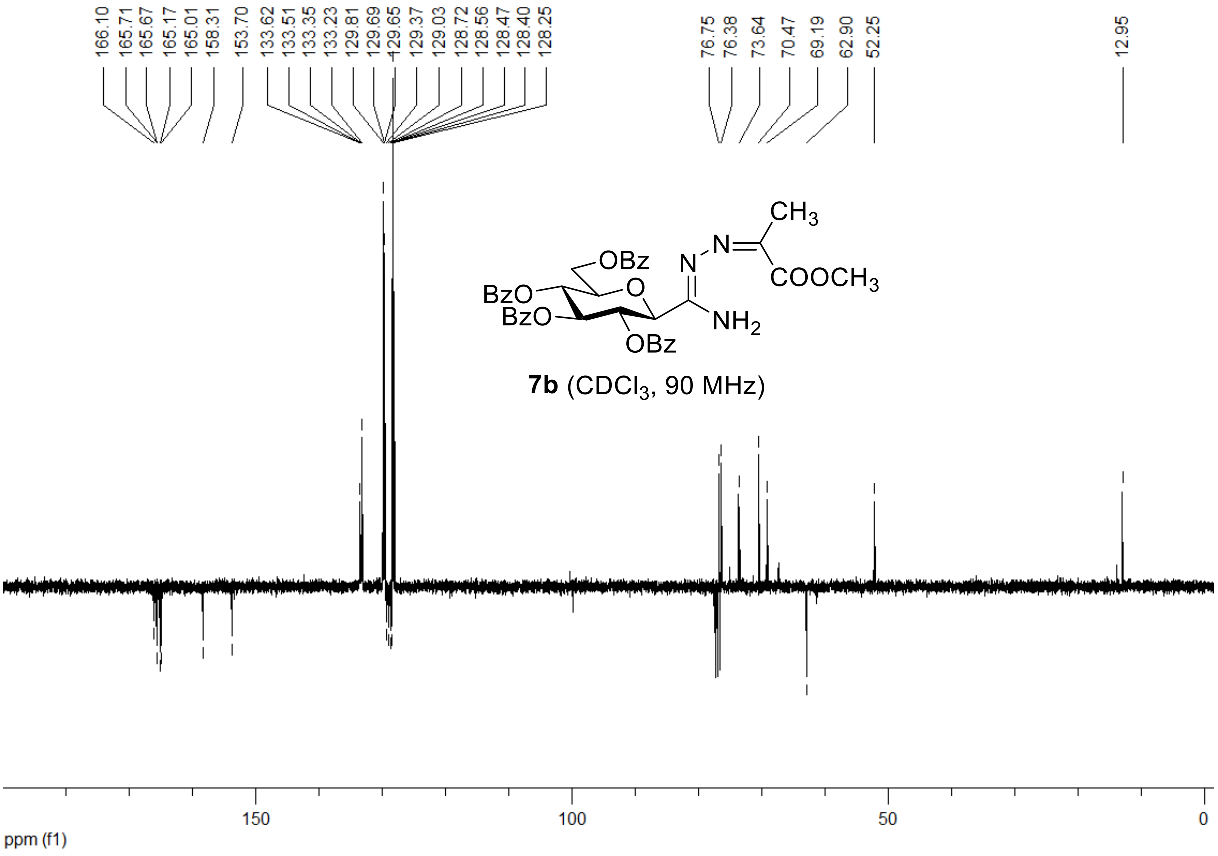
166.10 165.71 165.67 165.17 165.01 158.31 153.70 133.62 133.51 133.35 133.23 129.81 129.69 129.65 129.37 129.03 128.72 128.56 128.47 128.40 128.25 76.75 76.38 73.64 70.47 69.19 62.90 52.25 12.95
CH3
N
N
OBz
BzO
O
BzO
COOCH3
NH2
OBz
7b (CDCl3, 90 MHz)
150 100 50 0
ppm (f1)

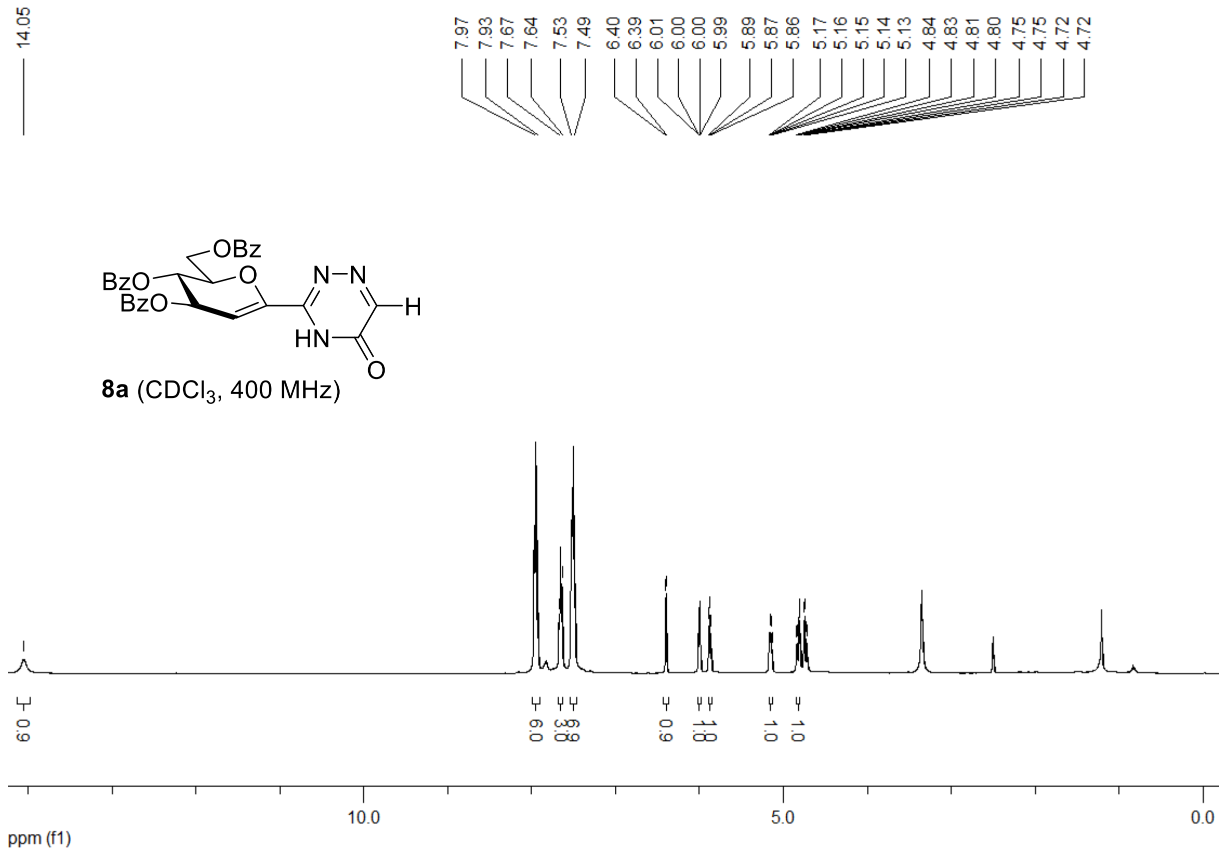
14.05
7.97 7.93 7.67 7.64 7.53 7.49 6.40 6.39 6.01 6.00 6.00 5.99 5.89 5.87 5.86 5.17 5.16 5.15 5.14 5.13 4.84 4.83 4.81 4.80 4.75 4.75 4.72 4.72
OBz
BzO
BzO
O
N–N
H
HN
O
8a (CDCl3, 400 MHz)
0.9
6.0
3.0
6.9
0.9
1.8
1.0
1.0
10.0
5.0
0.0
ppm (f1)

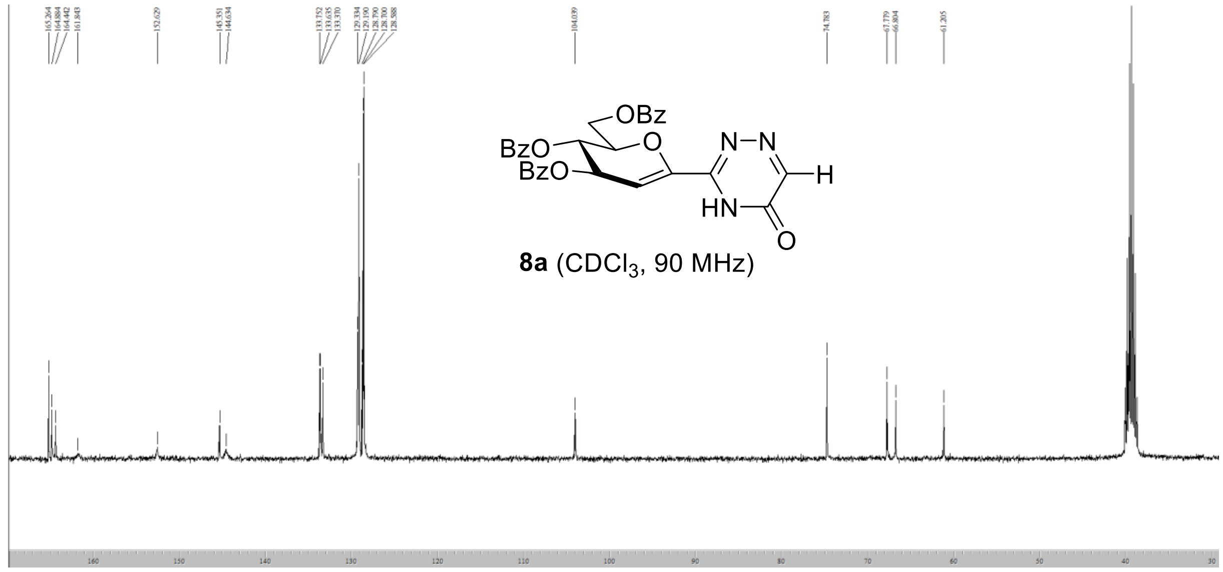
OBz
BzO
BzO
O
N–N
H
HN
O
8a (CDCl3, 90 MHz)

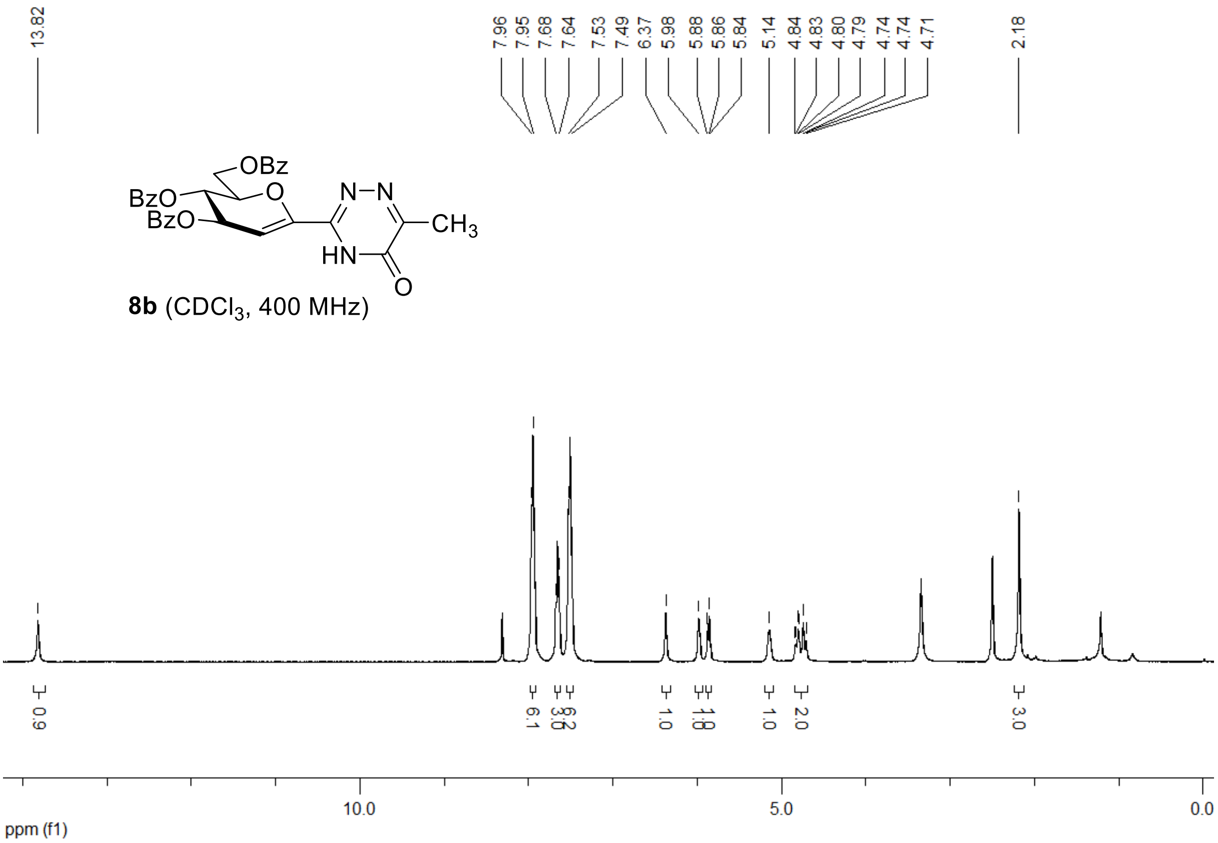
13.82
7.96 7.95 7.68 7.64 7.53 7.49 6.37 5.98 5.88 5.86 5.84 5.14 4.84 4.83 4.80 4.79 4.74 4.74 4.71
2.18
OBz
BzO
BzO
O
N−N
CH3
HN
O
8b (CDCl3, 400 MHz)
0.9 6.1 3.0 6.2 1.0 1.0 1.0 1.0 2.0 3.0
10.0
5.0
0.0
ppm (f1)

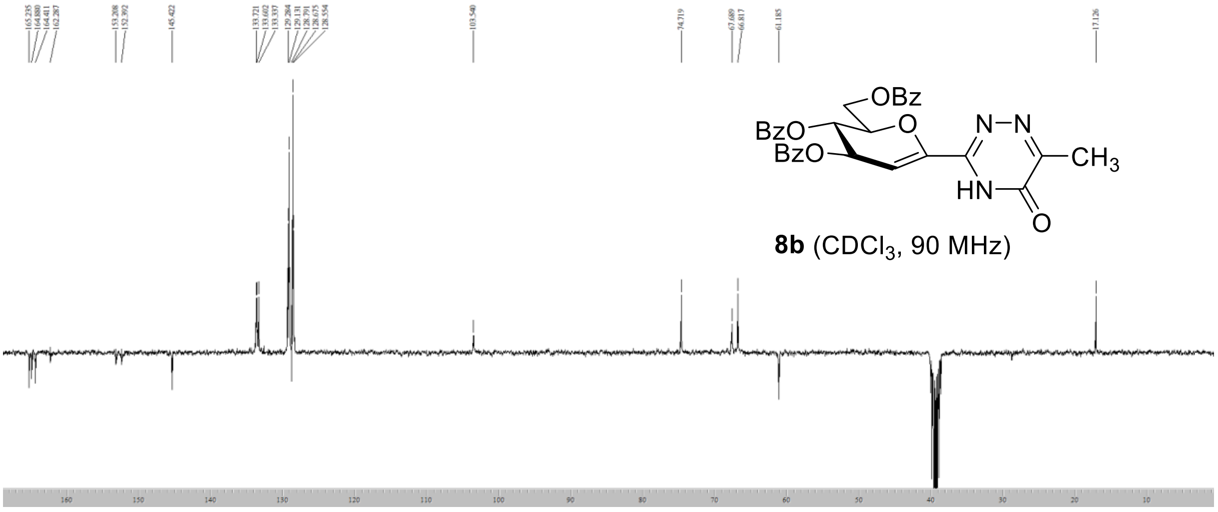
OBz
BzO
BzO
O
N−N
CH3
HN
O
8b (CDCl3, 90 MHz)

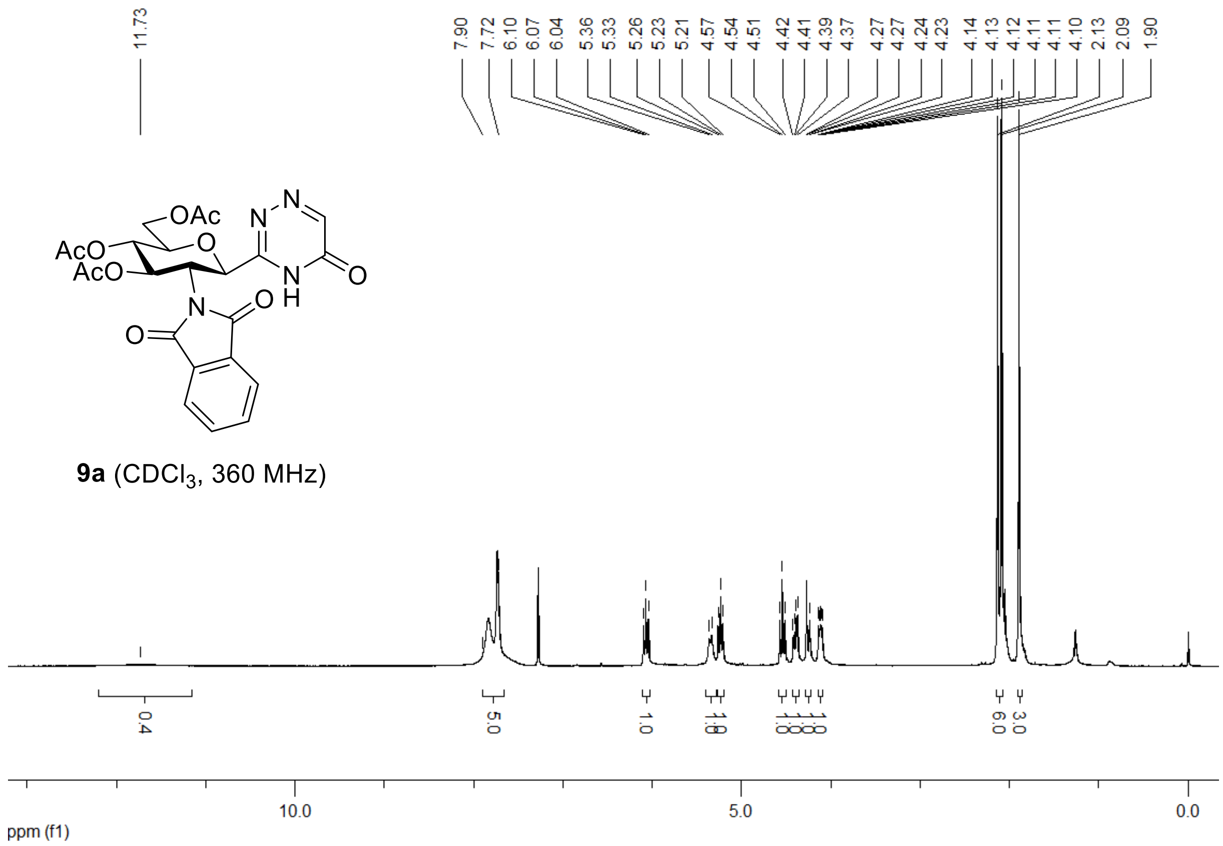


**9a** (CDCl$_3$, 360 MHz)

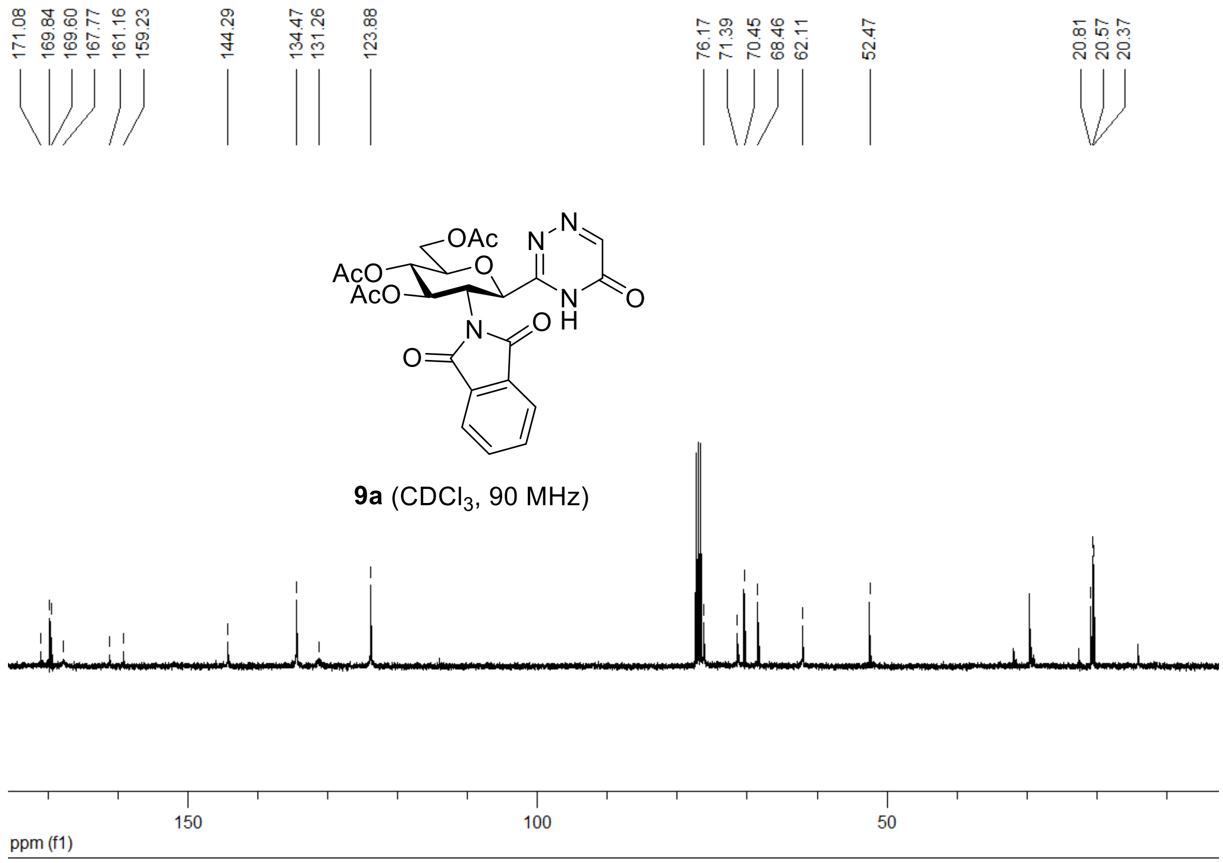


**9a** (CDCl$_3$, 90 MHz)

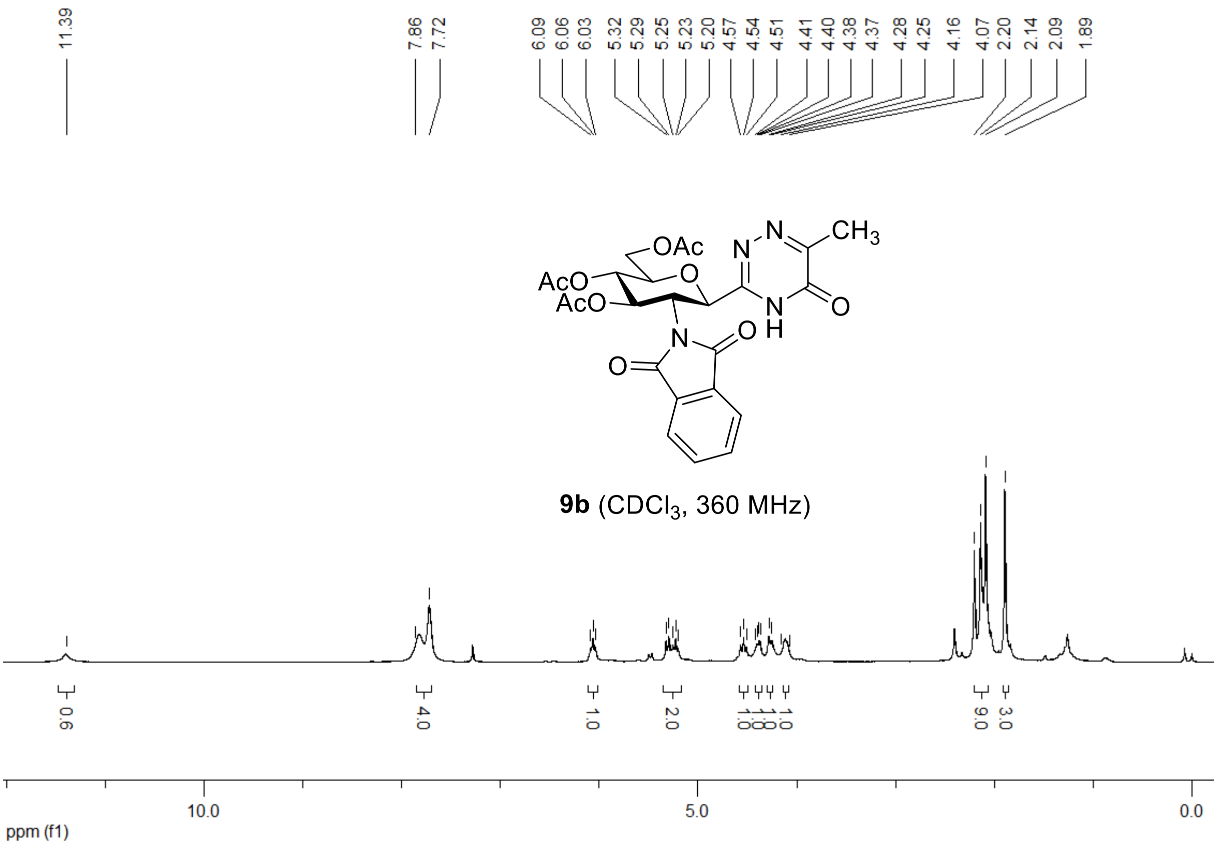

**9b** ($CDCl_3$, 360 MHz)

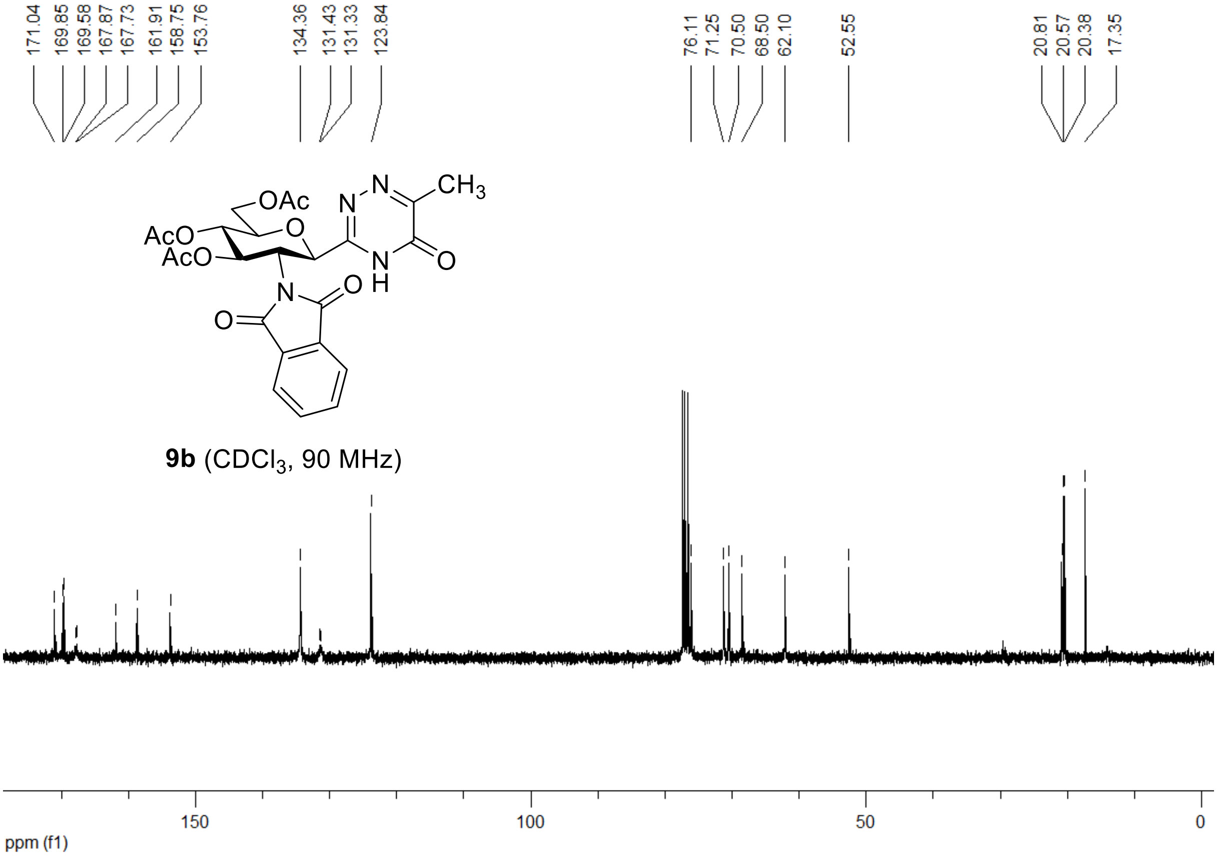

**9b** ($CDCl_3$, 90 MHz)

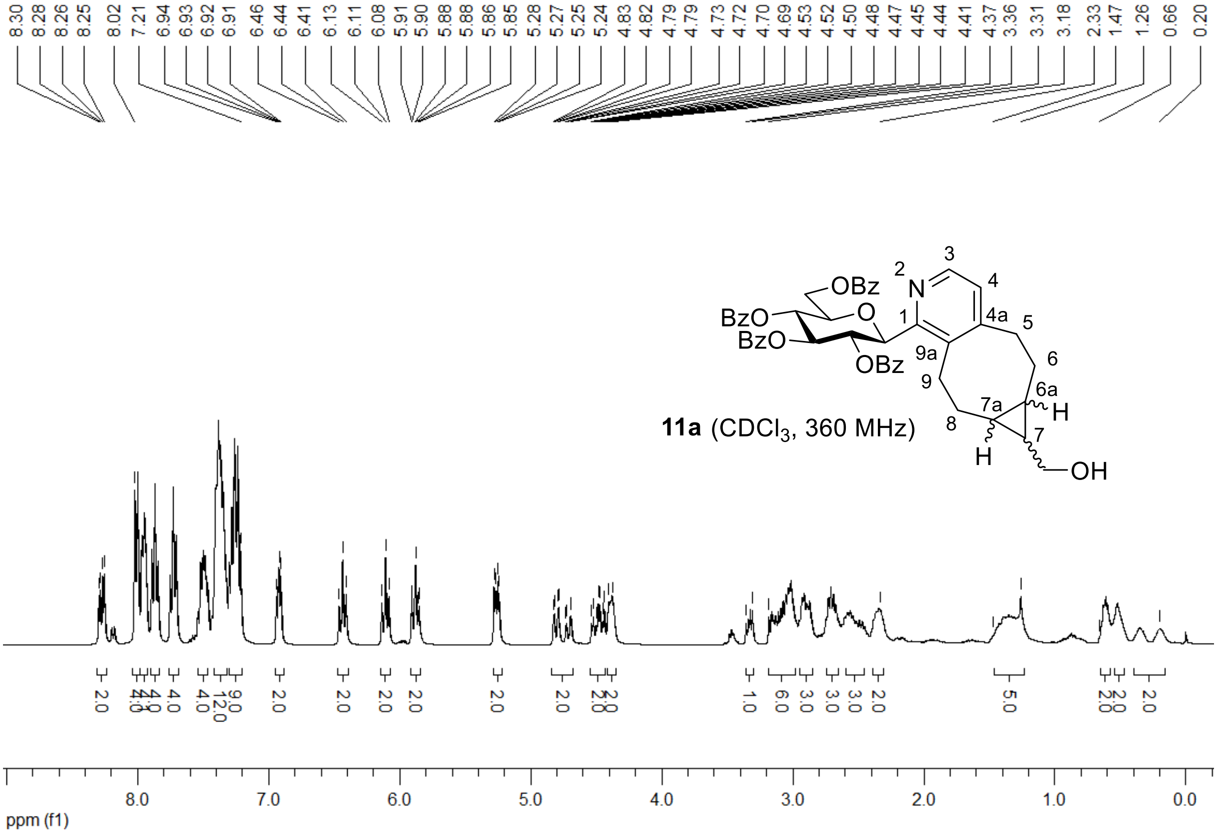
8.30 8.28 8.26 8.25 8.02 7.21 6.94 6.93 6.92 6.91 6.46 6.44 6.41 6.13 6.11 6.08 5.91 5.90 5.88 5.88 5.86 5.85 5.28 5.27 5.25 5.24 4.83 4.82 4.79 4.79 4.73 4.72 4.70 4.69 4.53 4.52 4.50 4.48 4.47 4.45 4.44 4.41 4.37 3.36 3.31 3.18 2.33 1.47 1.26 0.66 0.20
BzO
BzO
OBz
OBz
O
N
1 2 3 4 4a 5 6 6a 7 7a 8 9 9a
H
H
OH
11a (CDCl3, 360 MHz)
ppm (f1)
8.0 7.0 6.0 5.0 4.0 3.0 2.0 1.0 0.0

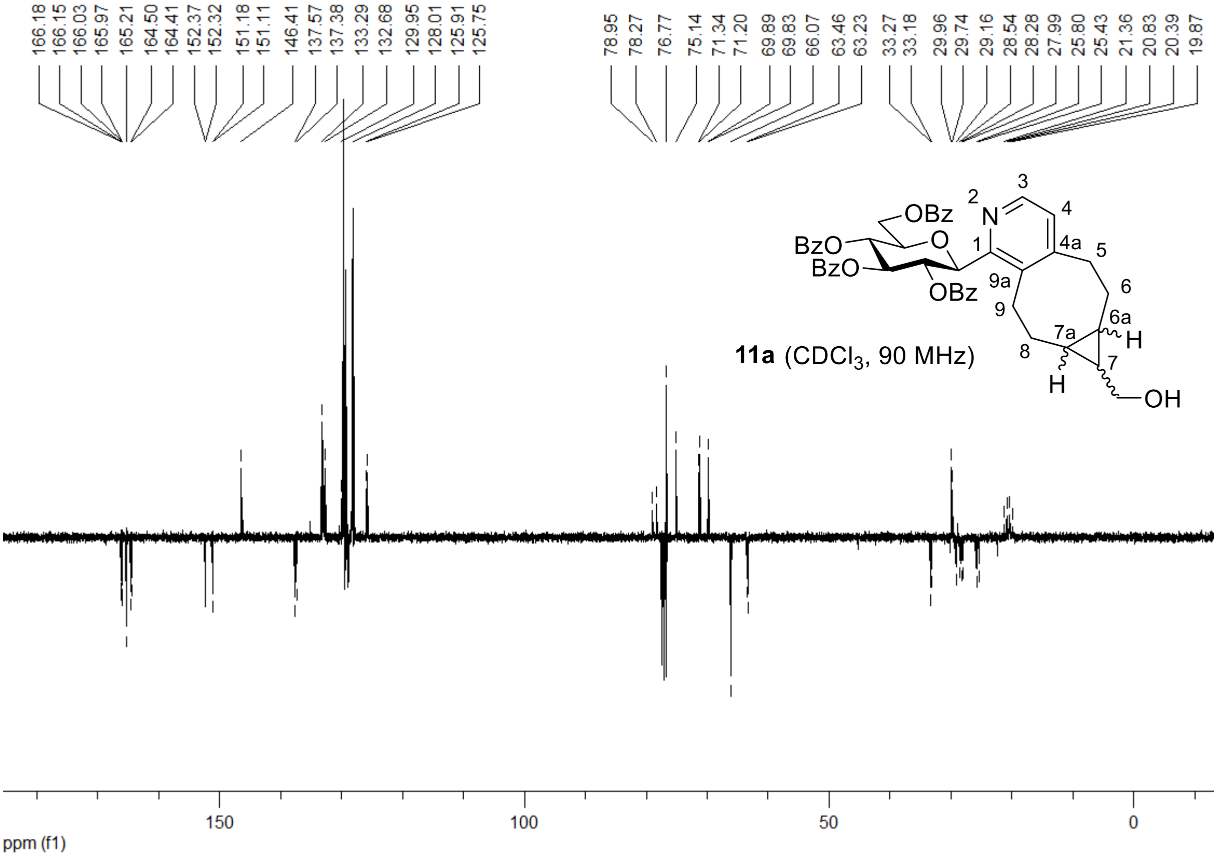
166.18 166.15 166.03 165.97 165.21 164.50 164.41 152.37 152.32 151.18 151.11 146.41 137.57 137.38 133.29 132.68 129.95 128.01 125.91 125.75
78.95 78.27 76.77 75.14 71.34 71.20 69.89 69.83 66.07 63.46 63.23 33.27 33.18 29.96 29.74 29.16 28.54 28.28 27.99 25.80 25.43 21.36 20.83 20.39 19.87
BzO
BzO
OBz
OBz
O
N
1 2 3 4 4a 5 6 6a 7 7a 8 9 9a
H
H
OH
11a (CDCl3, 90 MHz)
ppm (f1)
150 100 50 0

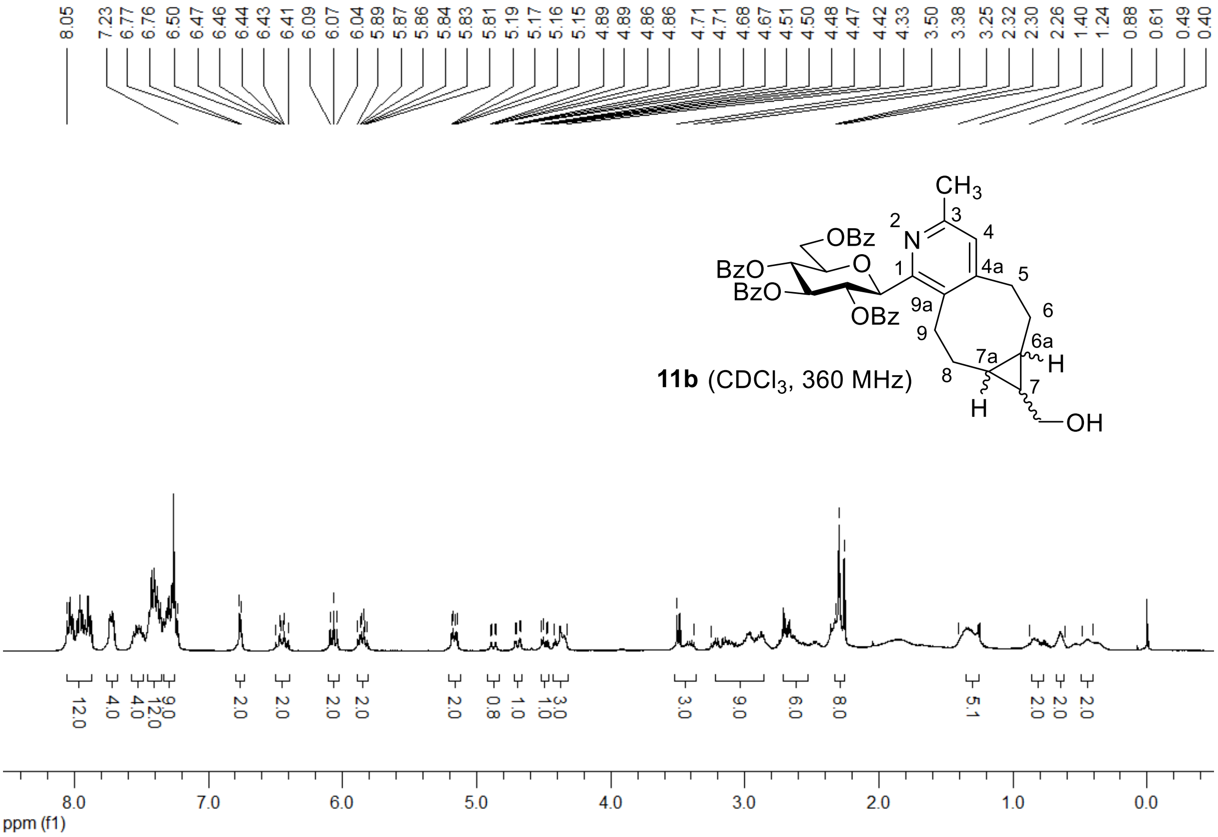
8.05
7.23
6.77
6.76
6.50
6.47
6.46
6.44
6.43
6.41
6.09
6.07
6.04
5.89
5.87
5.86
5.84
5.83
5.81
5.19
5.17
5.16
5.15
4.89
4.89
4.86
4.86
4.71
4.71
4.68
4.67
4.51
4.50
4.48
4.47
4.42
4.33
3.50
3.38
3.25
2.32
2.30
2.26
1.40
1.24
0.88
0.61
0.49
0.40
CH3
BzO
BzO
OBz
OBz
O
N
OH
H
H
11b (CDCl3, 360 MHz)
12.0
4.0
4.0
12.0
9.0
2.0
2.0
2.0
2.0
2.0
0.8
1.0
1.0
3.0
3.0
9.0
6.0
8.0
5.1
2.0
2.0
2.0
8.0
7.0
6.0
5.0
4.0
3.0
2.0
1.0
0.0
ppm (f1)

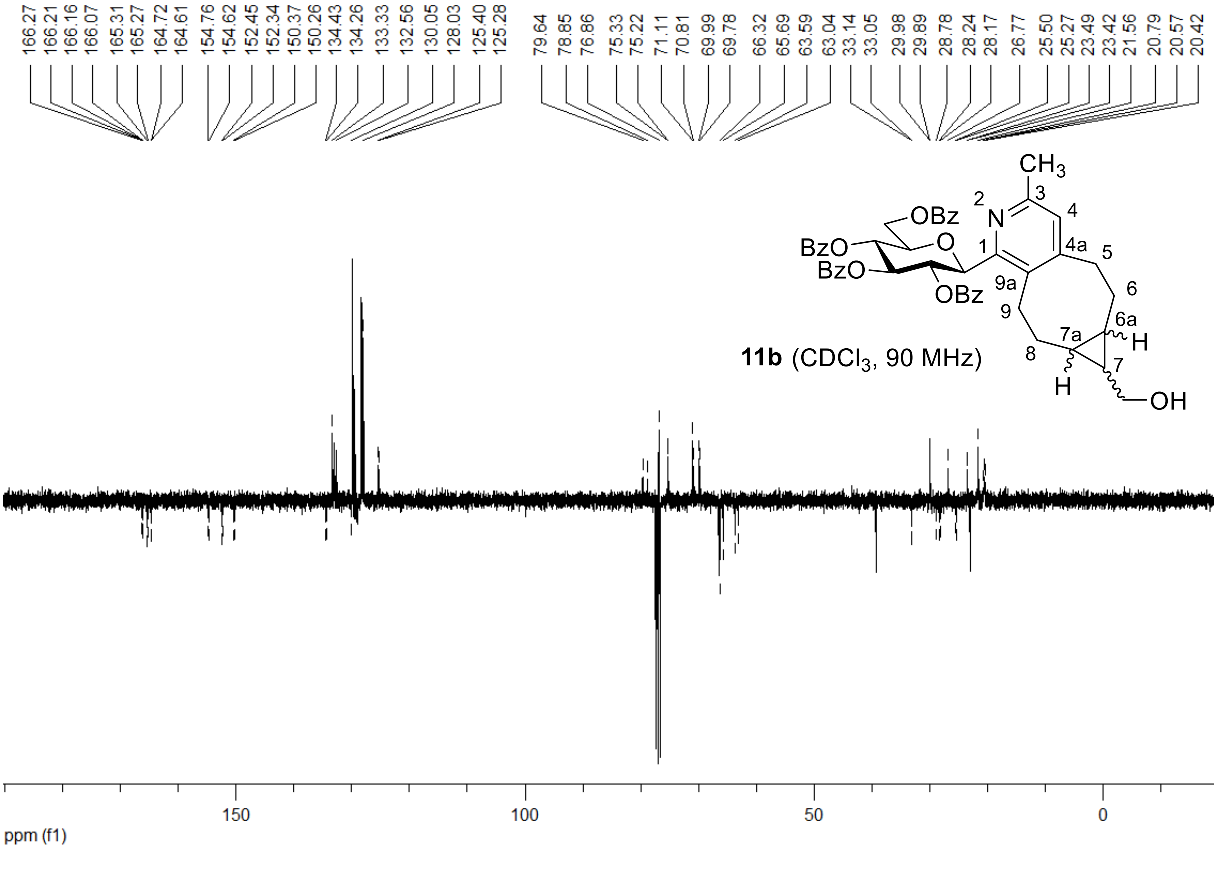
166.27
166.21
166.16
166.07
165.31
165.27
164.72
164.61
154.76
154.62
152.45
152.34
150.37
150.26
134.43
134.26
133.33
132.56
130.05
128.03
125.40
125.28
79.64
78.85
76.86
75.33
75.22
71.11
70.81
69.99
69.78
66.32
65.69
63.59
63.04
33.14
33.05
29.98
29.89
28.78
28.24
28.17
26.77
25.50
25.27
23.49
23.42
21.56
20.79
20.57
20.42
CH3
BzO
BzO
OBz
OBz
O
N
OH
H
H
11b (CDCl3, 90 MHz)
150
100
50
0
ppm (f1)

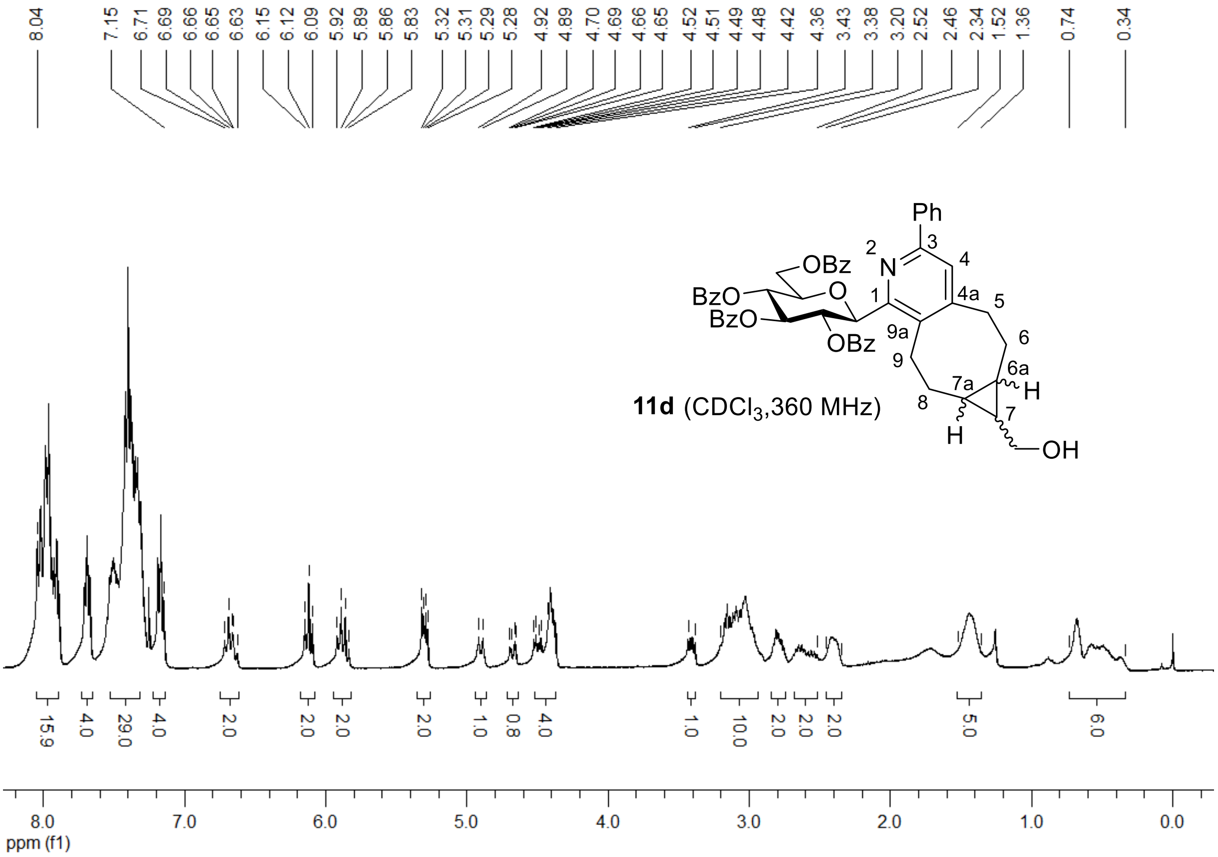

8.04
7.15
6.71
6.69
6.66
6.65
6.63
6.15
6.12
6.09
5.92
5.89
5.86
5.83
5.32
5.31
5.29
5.28
4.92
4.89
4.70
4.69
4.66
4.65
4.52
4.51
4.49
4.48
4.42
4.36
3.43
3.38
3.20
2.52
2.46
2.34
1.52
1.36
0.74
0.34
Ph
N
OBz
BzO
BzO
O
OBz
OH
H
H
11d (CDCl3,360 MHz)
15.9
4.0
29.0
4.0
2.0
2.0
2.0
2.0
1.0
0.8
4.0
1.0
10.0
2.0
2.0
2.0
5.0
6.0
ppm (f1)


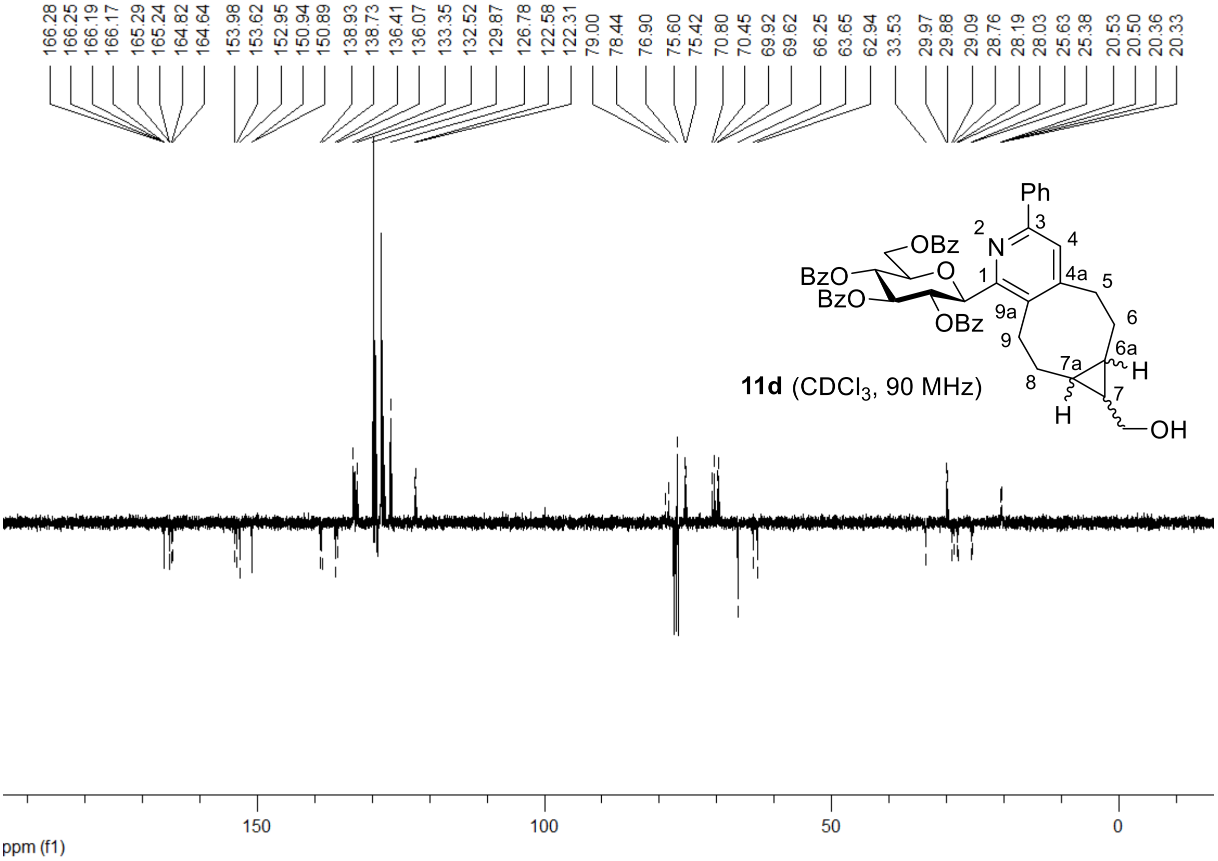

166.28
166.25
166.19
166.17
165.29
165.24
164.82
164.64
153.98
153.62
152.95
150.94
150.89
138.93
138.73
136.41
136.07
133.35
132.52
129.87
126.78
122.58
122.31
79.00
78.44
76.90
75.60
75.42
70.80
70.45
69.92
69.62
66.25
63.65
62.94
33.53
29.97
29.88
29.09
28.76
28.19
28.03
25.63
25.38
20.53
20.50
20.36
20.33
11d (CDCl3, 90 MHz)
ppm (f1)

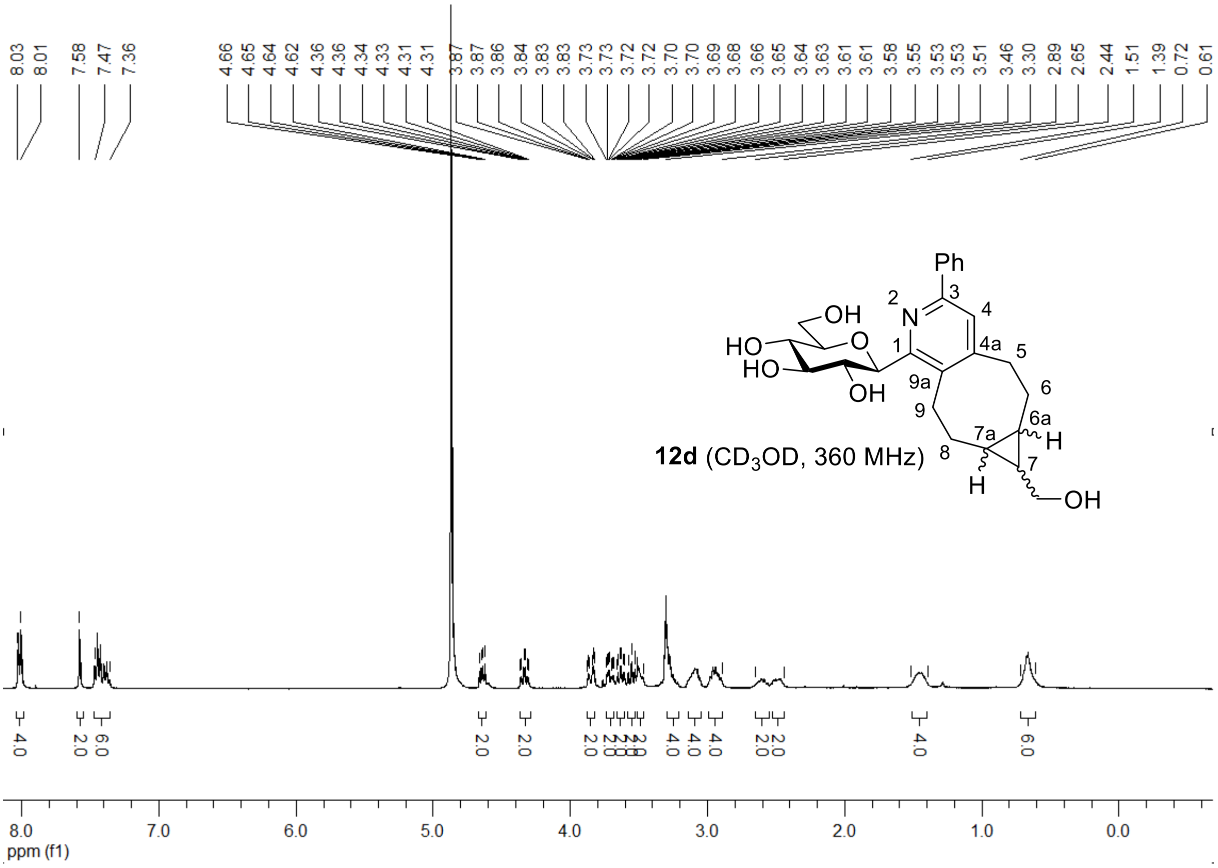
8.03 8.01 7.58 7.47 7.36 4.66 4.65 4.64 4.62 4.36 4.36 4.34 4.33 4.31 4.31 3.87 3.87 3.86 3.84 3.83 3.83 3.73 3.73 3.72 3.72 3.70 3.70 3.69 3.68 3.66 3.65 3.64 3.63 3.61 3.61 3.58 3.55 3.53 3.53 3.51 3.46 3.30 2.89 2.65 2.44 1.51 1.39 0.72 0.61
Ph
HO
HO
OH
OH
O
N
OH
H
H
12d (CD$_3$OD, 360 MHz)
4.0 2.0 6.0 2.0 2.0 2.0 2.0 2.0 2.0 2.0 4.0 4.0 4.0 2.0 2.0 4.0 6.0
8.0 7.0 6.0 5.0 4.0 3.0 2.0 1.0 0.0
ppm (f1)

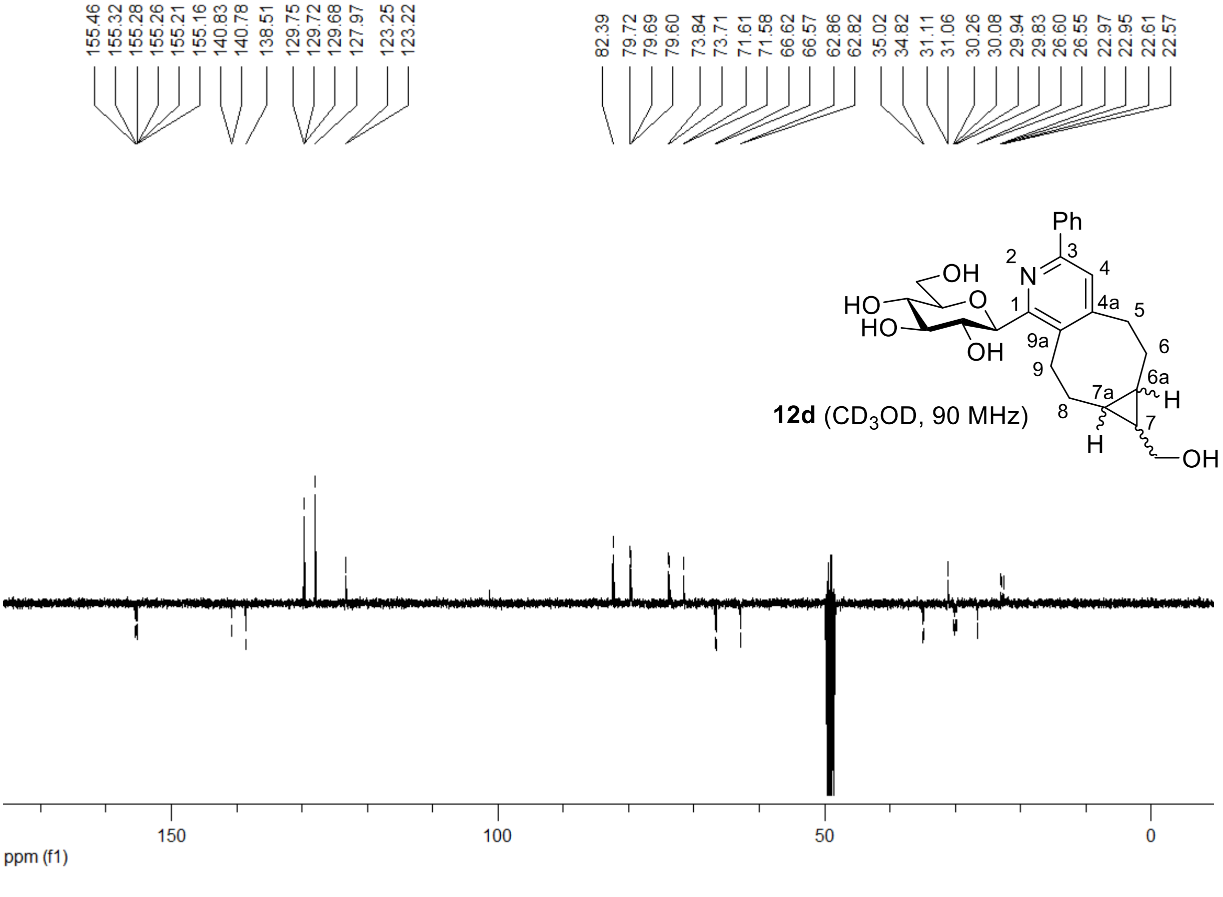
155.46 155.32 155.28 155.26 155.21 155.16 140.83 140.78 138.51 129.75 129.72 129.68 127.97 123.25 123.22 82.39 79.72 79.69 79.60 73.84 73.71 71.61 71.58 66.62 66.57 62.86 62.82 35.02 34.82 31.11 31.06 30.26 30.08 29.94 29.83 26.60 26.55 22.97 22.95 22.61 22.57
Ph
HO
HO
OH
OH
O
N
OH
H
H
12d (CD$_3$OD, 90 MHz)
150 100 50 0
ppm (f1)
